# A Comprehensive Review of Antimicrobial Peptides and Smart Biomaterials in Chronic Wound Therapy: Overcoming Biofilms, Resistance, and Translational Barriers

**DOI:** 10.3390/ijms27135955

**Published:** 2026-07-02

**Authors:** Laura Maghiar, Paula Bianca Maghiar, Ovidiu Pop, Anca Maria Mitran, Mihaela Mirela Muresan, Andreea-Adriana Neamțu, Dan Iliescu, Dan Brebu, Paul Andrei Tent, Florian Dorel Bodog, Valentin-Cristian Iovin, Cristina Dumitrescu, Andreea Maria Cristea, Alina Anton, Andrada Iftode, Florin Huț, Cristina-Adriana Dehelean, Alina Hegheș

**Affiliations:** 1 Department of Psycho-Neurosciences and Rehabilitation, Faculty of Medicine and Pharmacy, University of Oradea, Universității Str., No. 1, 410087 Oradea, Romania; laura.maghiar@uoradea.ro; 2 Department of Dermatovenerology, Clinical County Emergency Hospital Bihor, 410169 Oradea, Romania; 3 Doctoral School of Biomedical Sciences, University of Oradea, Universității Str., No. 1, 410087 Oradea, Romania; pao.badea@gmail.com (P.B.M.); drovipop@yahoo.com (O.P.); muresan.mihaelamirela@student.uoradea.ro (M.M.M.); 4 Department of Surgical Disciplines, Faculty of Medicine and Pharmacy, University of Oradea, Universității Str., No. 1, 410087 Oradea, Romania; 5 Department of Surgery, Pelican Hospital, Corneliu Coposu Str., No. 2, 410450 Oradea, Romania; 6 Department of Anatomic Pathology, Clinical County Emergency Hospital Bihor, 410169 Oradea, Romania; 7 Department of Morphological Disciplines, Faculty of Medicine and Pharmacy, University of Oradea, 410073 Oradea, Romania; 8 Doctoral School of University of Medicine and Pharmacy of Craiova, 200349 Craiova, Romania; anca.mitran@umfcv.ro; 9 Department of Pharmacy, Poiana Mare Psychiatry Hospital, Gării Str., No. 40, 207470 Poiana Mare, Romania; 10 Department of Toxicology, “Victor Babes” University of Medicine and Pharmacy, Eftimie Murgu Square, No. 2, 300041 Timisoara, Romania; cristina.grosu@umft.ro (C.D.); andreea.cristea@umft.ro (A.M.C.); dolghi.alina@umft.ro (A.A.); andradaiftode@umft.ro (A.I.); cadehelean@umft.ro (C.-A.D.); 11 Research Centre for Pharmaco-Toxicological Evaluation, “Victor Babes” University of Medicine and Pharmacy, Eftimie Murgu Square, No. 2, 300041 Timisoara, Romania; 12 Department of Pathology, Clinical County Emergency Hospital of Arad, Andrenyi Karoly Str., No. 2–4, 310037 Arad, Romania; 13 Department of Pathology, “Pius Brinzeu” Clinical County Emergency Hospital Timisoara, Liviu Rebreanu Boulevard, No. 156, 300723 Timisoara, Romania; 14 Department of Surgery I, Clinic of Surgical Semiotics & Thoracic Surgery, Center for Hepato-Biliary and Pancreatic Surgery, Faculty of Medicine, “Victor Babes” University of Medicine and Pharmacy, Eftimie Murgu Square, No. 2, 300041 Timisoara, Romania; florin.hut@umft.ro; 15 II Surgery Clinic, Pius Brinzeu” Clinical County Emergency Hospital Timisoara, Liviu Rebreanu Boulevard, No. 156, 300723 Timisoara, Romania; brebu.dan@umft.ro; 16 Department X of General Surgery, “Victor Babes” University of Medicine and Pharmacy, Eftimie Murgu Square, No. 2, 300041 Timisoara, Romania; 17 Department of Oral and Maxillo-Facial Surgery, Faculty of Medicine and Pharmacy, University of Oradea, Universității Str., No. 1, 410087 Oradea, Romania; tent_andrei@yahoo.com; 18 Plastic and Reconstructive Surgery Department, Clinical County Emergency Hospital Bihor, 410169 Oradea, Romania; 19 Doctoral School Department, “Victor Babes” University of Medicine and Pharmacy, 300041 Timisoara, Romania; 20 Department III Functional Sciences, Physiology Discipline, “Victor Babes” University of Medicine and Pharmacy, 300041 Timisoara, Romania; 21 Centre of Immuno-Physiology and Biotechnologies (CIFBIOTEH), Department of Functional Sciences, Physiology, “Victor Babes” University of Medicine and Pharmacy, 300041 Timisoara, Romania; 22 Department II, Pharmaceutical Technology, Faculty of Pharmacy, “Victor Babes” University of Medicine and Pharmacy, Eftimie Murgu Square, No. 2, 300041 Timisoara, Romania; heghes.alina@umft.ro; 23 Formulation and Technology of Drugs Research Center, Faculty of Pharmacy, “Victor Babes” University of Medicine and Pharmacy, Eftimie Murgu Square, No. 2, 300041 Timisoara, Romania

**Keywords:** chronic wound, antimicrobial peptides, biofilm, smart biomaterials, antimicrobial resistance, wound healing, antibiofilm therapy, regenerative medicine, peptide delivery systems, translational medicine

## Abstract

Chronic wounds represent a growing global healthcare burden driven by persistent inflammation, polymicrobial biofilm formation, impaired tissue regeneration, and increased antimicrobial resistance. This review examines the mechanistic interplay between chronic wound pathophysiology, biofilm persistence, and antimicrobial peptide (AMP)-based therapeutics, with particular emphasis on translational barriers and advanced biomaterial-enabled delivery strategies. Current evidence demonstrates that AMPs exert multifactorial activities extending beyond direct antimicrobial effects, including membrane disruption, quorum-sensing inhibition, extracellular polymeric substance (EPS) destabilization, immune modulation, angiogenic stimulation, and promotion of re-epithelialization. However, their clinical translation remains limited due to proteolytic degradation, poor stability, cytotoxicity, rapid clearance, and inadequate retention within the hostile chronic wound microenvironment. To address these limitations, emerging biomaterial platforms—including hydrogels, electrospun nanofibers, nanoparticles, self-assembling peptide systems, and stimuli-responsive smart dressings—have been developed to improve AMP stability, controlled release, biofilm penetration, and regenerative efficacy. This review further highlights current preclinical and clinical challenges, including the lack of standardized polymicrobial biofilm models and translationally relevant wound systems, while discussing future perspectives such as artificial intelligence-assisted peptide design and precision wound therapeutics. We argue that peptide discovery is no longer the principal bottleneck: the rate-limiting steps are now peptide stabilization, biofilm-targeted delivery, and dosing, and no current platform yet couples validated eradication of mature polymicrobial biofilms with validated tissue regeneration in a clinically representative model. Collectively, AMP-enabled smart biomaterials may support the transition from passive wound management toward responsive, biofilm-targeted regenerative therapy.

## 1. Introduction

Chronic wounds represent a major and increasingly underestimated global healthcare burden, affecting millions of patients worldwide and generating substantial socioeconomic costs through prolonged hospitalization, recurrent infections, disability, and limb amputation [1,2]. Among the most clinically significant chronic wounds are diabetic foot ulcers (DFUs), venous leg ulcers, pressure ulcers, and severe burn wounds, all of which are associated with impaired tissue regeneration and a high risk of infection-related complications [3]. The growing prevalence of diabetes, obesity, vascular disease, and population aging has further accelerated the incidence of chronic non-healing wounds, transforming them into a significant public health challenge [4]. Notably, diabetic foot ulcers are associated with alarming morbidity and mortality rates, with five-year mortality exceeding that of several common malignancies [5].

A central factor contributing to wound chronicity is persistent microbial colonization and biofilm formation. More than 80% of chronic wound infections involve biofilm-forming microorganisms, which substantially reduce antibiotic susceptibility and impair tissue regeneration [6]. Biofilms are highly organized microbial communities embedded within a self-produced extracellular polymeric substance (EPS) matrix that protects pathogens against host immune defenses, antimicrobial agents, and environmental stressors [7]. Within chronic wounds, biofilm formation perpetuates inflammation, promotes oxidative stress, disrupts angiogenesis, and impairs re-epithelialization, thereby creating a self-sustaining pathological microenvironment that prevents wound resolution [8]. Furthermore, polymicrobial biofilms frequently contain multidrug-resistant (MDR) pathogens such as methicillin-resistant *Staphylococcus aureus* (MRSA), *Pseudomonas aeruginosa*, *Enterococcus faecalis*, and *Acinetobacter baumannii*, which further complicate therapeutic management [9].

The rapid escalation of antimicrobial resistance (AMR) has significantly reduced the efficacy of conventional antibiotics in chronic wound care [10]. Standard antimicrobial therapies are often unable to eradicate mature biofilms because of poor penetration through the EPS matrix, altered bacterial metabolic states, quorum-sensing-mediated resistance, and the presence of persister cells [11]. In addition, repeated antibiotic exposure contributes to the emergence of resistant strains and may disrupt the physiological wound microbiota, thereby impairing tissue repair [12]. Consequently, there is an urgent need for alternative therapeutic strategies capable of simultaneously controlling infection, disrupting biofilms, and promoting tissue regeneration.

Antimicrobial peptides (AMPs) have emerged as highly promising candidates for chronic wound management because of their broad-spectrum antimicrobial activity, rapid bactericidal mechanisms, low propensity for resistance development, and multifunctional immunomodulatory properties [9,13]. Unlike conventional antibiotics that often target single intracellular pathways, AMPs exert diverse mechanisms of action, including membrane disruption, intracellular targeting, quorum-sensing interference, and biofilm destabilization. Importantly, many AMPs also exhibit pro-regenerative activities by modulating inflammation, stimulating angiogenesis, enhancing keratinocyte migration, and promoting fibroblast proliferation, thereby directly contributing to wound healing processes [14,15]. These multifunctional properties position AMPs as attractive therapeutic agents for the treatment of infected chronic wounds.

Despite their therapeutic potential, the clinical translation of AMPs remains limited by several major challenges. Free peptides are highly susceptible to enzymatic degradation within the protease-rich chronic wound environment, while additional limitations include poor stability, short half-life, potential cytotoxicity, salt sensitivity, and inadequate retention at the wound site [16,17]. Therefore, the major challenge is not AMP discovery anymore, but AMP stabilization, delivery, antibiofilm efficacy, and clinical translation. Two complementary routes have emerged to address this challenge. At the molecular level, chemical and structural engineering of the peptides themselves—including D-amino acid substitution, backbone cyclization and stapling, terminal modification and lipidation, and the design of protease-resistant peptidomimetics—can improve proteolytic stability while tuning antimicrobial activity and host–cell selectivity (Section 5.2) [17]. At the formulation level,, advanced biomaterial-based delivery systems have emerged as essential enabling technologies capable of protecting AMPs from degradation, improving controlled release, enhancing biofilm penetration, and optimizing local therapeutic efficacy [18]. Hydrogels, electrospun nanofibers, scaffolds, films, nanoparticles, and multifunctional smart dressings are increasingly being engineered to provide sustained AMP delivery while simultaneously supporting tissue regeneration and wound microenvironment modulation [19].

Unlike previous AMP-centered reviews focused primarily on peptide classification or biomaterial inventories, this review is built around a single argument: that peptide discovery is no longer the principal obstacle to AMP-based wound therapy, and that the decisive challenges are now peptide stabilization, biofilm-targeted delivery, dosing, and clinically relevant validation. To advance this argument rather than catalogue peptides or carriers exhaustively, we resolve representative AMP-enabled systems by their bacterial target, trigger mechanism, and reported quantitative antibiofilm and regenerative outcomes, and by their level of evidence—in vitro, preclinical in vivo, or clinical—in dedicated comparison tables; we distinguish validated artificial-intelligence applications in peptide discovery and optimization from still-emerging uses in peptide–carrier co-design; and we identify, as the field’s principal evidence gap, the absence of any platform that couples validated eradication of mature polymicrobial biofilms with validated tissue regeneration in a single, clinically representative model. Particular attention is given to the interaction between chronic wound pathophysiology, biofilm persistence, AMP therapeutic mechanisms, and the emerging generation of smart biomaterial platforms designed to overcome current clinical limitations. Additionally, this review discusses preclinical and clinical evidence, current translational bottlenecks, and future perspectives, including artificial intelligence-assisted AMP design and precision wound therapeutics.

The review is organised to progressively link the biology of chronic wounds with emerging AMP-enabled therapeutic approaches. Section 2 examines the biological processes involved in chronic wounds and microbial biofilm formation. Section 3 summarizes current clinical guidelines and unsolved translational limitations in biofilm-infected wounds. Section 4 discusses antimicrobial peptides as multifunctional antibiofilm and regenerative agents, focusing on structural classes, mechanisms of action and connections with biofilm biology. Section 5 describes the primary translational barriers that impede free-AMP therapy in the chronic wound setting. Section 6 discusses biomaterial-assisted AMP delivery strategies, including hydrogels, electrospun nanofibers, nanoparticles, and multifunctional smart dressings. Section 7 is dedicated to the pathology-responsive and stimuli-adaptive AMP biomaterials. Section 8 summarizes the current translational evidence from preclinical and clinical studies, while Section 9 explores future perspectives, including artificial intelligence-assisted peptide engineering, microbiome-informed treatments, and advanced validation models. Finally, Section 10 outlines the translational prospects of AMP-enabled regenerative wound therapeutics, concluding the review.

## 2. Pathophysiology of Chronic Wounds and Biofilm Formation Relevant to AMP-Based Therapy

Chronic wounds arise from a complex interplay between dysregulated host responses, microbial persistence, impaired tissue regeneration, and pathological wound microenvironments. Unlike acute wounds, which progress through tightly coordinated phases of healing, chronic wounds become trapped in a sustained inflammatory state characterized by excessive protease activity, oxidative stress, senescence, vascular dysfunction, and persistent microbial colonization [8,9,16]. Importantly, biofilm formation should not be viewed merely as a secondary consequence of chronicity, but rather as an active pathogenic driver that perpetuates inflammation, impairs immune clearance, and contributes to antimicrobial tolerance and resistance [6,7,11]. Increasing evidence suggests that wound chronicity and biofilm persistence form a self-reinforcing pathological cycle in which inflammation promotes microbial colonization, while biofilms further exacerbate tissue damage, ROS accumulation, hypoxia, and proteolytic degradation of endogenous repair mediators, including host antimicrobial peptides (AMPs) [7,9,12,13].

### 2.1. Normal Wound Healing and Endogenous Antimicrobial Defense

Because antimicrobial peptides operate at the interface of microbial control and tissue repair, the normal healing cascade—including the endogenous AMPs released by platelets and neutrophils during hemostasis and inflammation—defines both the regenerative endpoints and the natural peptide functions that exogenous AMP therapies aim to reinforce. Physiological wound healing is a dynamic and highly regulated process classically divided into four overlapping phases: hemostasis, inflammation, proliferation, and remodeling [8,9,20,21,22]. These stages involve coordinated interactions among platelets, immune cells, fibroblasts, endothelial cells, keratinocytes, extracellular matrix (ECM) components, cytokines, and growth factors [8,9,20].

**Hemostasis**

Immediately following tissue injury, vascular disruption triggers platelet activation and coagulation cascade initiation, resulting in fibrin clot formation [8,23,24]. Beyond providing mechanical hemostasis, the provisional fibrin matrix functions as a biologically active scaffold that concentrates cytokines and growth factors, including platelet-derived growth factor (PDGF), transforming growth factor-β (TGF-β), vascular endothelial growth factor (VEGF), and stromal-derived factor-1 (SDF-1/CXCL12), thereby orchestrating subsequent inflammatory and reparative events [8,24,25,26]. Platelets also contribute to innate immune defense through the release of antimicrobial mediators and chemotactic signals that recruit neutrophils and monocytes to the wound site [27,28].

**Inflammation**

The inflammatory phase is characterized by rapid recruitment of neutrophils followed by monocytes/macrophages [8,23,24]. Tissue injury releases pathogen-associated molecular patterns (PAMPs) and damage-associated molecular patterns (DAMPs), including HMGB1, ATP, hyaluronic acid fragments, and nucleic acids, which activate pattern-recognition receptors such as Toll-like receptors (TLRs) and nucleotide-binding oligomerization domain-like receptors (NLRs) [29,30]. Neutrophils provide early antimicrobial defense through phagocytosis, reactive oxygen species (ROS) generation, degranulation, and neutrophil extracellular trap (NET) formation [31,32]. Macrophages subsequently coordinate the transition from inflammation to tissue repair through phenotype switching from pro-inflammatory M1-like states toward reparative M2-like phenotypes [20,33]. This transition is essential for resolution of inflammation, angiogenesis, matrix deposition, and wound closure [20,33,34].

**Proliferation**

The proliferative phase involves re-epithelialization, angiogenesis, fibroblast activation, and granulation tissue formation [8,24,35]. Keratinocytes at wound margins migrate across the provisional matrix through dynamic integrin remodeling and matrix metalloproteinase (MMP)-mediated extracellular matrix (ECM) degradation [36,37,38]. Concurrently, fibroblasts proliferate and synthesize collagen-rich ECM components, while endothelial cells generate new vascular networks in response to hypoxia-induced VEGF signaling [24,39,40]. Granulation tissue formation restores metabolic support to the regenerating tissue and provides structural integrity for wound closure [24,41].

**Remodeling**

The remodeling phase may persist for months and involves progressive replacement of immature type III collagen with mechanically stronger type I collagen [8,24,42]. Myofibroblasts mediate wound contraction, while matrix metalloproteinases (MMPs) and tissue inhibitors of metalloproteinases (TIMPs) regulate extracellular matrix (ECM) turnover and scar maturation [38,43]. Successful remodeling requires balanced regulation of inflammation, angiogenesis, and matrix degradation; disturbances during this phase contribute to fibrosis, hypertrophic scarring, or chronic non-healing wounds [44,45].

**Figure 1:**
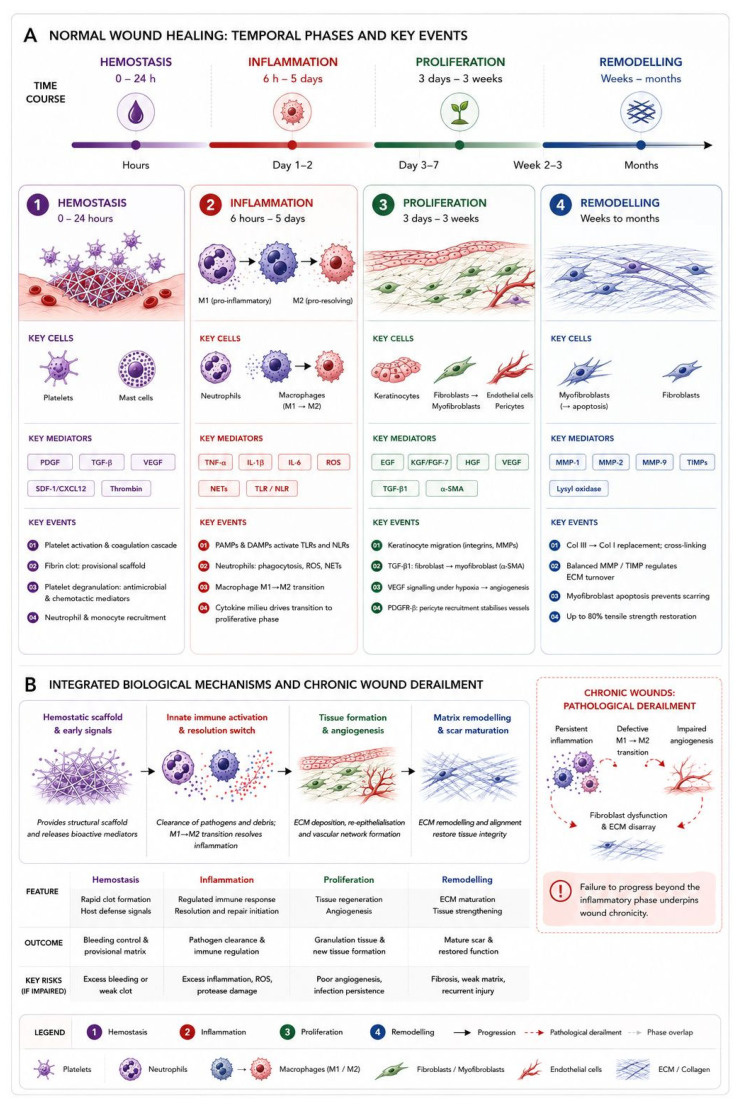
Physiological wound healing cascade and mechanisms underlying chronic wound formation. (**A**) Normal wound healing. The four overlapping phases—hemostasis (0–24 h), inflammation (6 h–5 days), proliferation (3 days–3 weeks), and remodelling (weeks–months)—with key cells, mediators, and events, from fibrin clot to M1→M2 switch, angiogenesis, and collagen III→I remodelling. (**B**) Mechanisms and chronic derailment. Sequential repair steps with a phase-by-phase table of features, outcomes, and risks; the right panel shows how persistent inflammation and defective M1→M2 transition derail healing into chronic wounds. Abbreviations: α-SMA, alpha smooth muscle actin; DAMPs, damage-associated molecular patterns; EMC, extracellular matrix; EGF, epidermal growth factor; KGF, keratinocyte growth factor; MMP, matrix metalloproteinase; NETs, neutrophil extracellular traps; NLR, NOD-like receptor; PAMPs, pathogen-associated molecular patterns; PDGF, platelet-derived growth factor; ROS, reactive oxygen species; SDF-1, stromal cell-derived factor-1; TGF-β; transforming growth factor-β; TIMP, tissue inhibitor of metalloproteinases; TLR, Toll-like receptor; VEGF, vascular endothelial growth factor.

Figure 1A illustrates the four temporally overlapping phases of physiological cutaneous wound healing: hemostasis, inflammation, proliferation, and remodeling. Following tissue injury the hemostatic phase is initiated with platelet activation and fibrin clot formation, which creates a provisional extracellular matrix scaffold rich in bioactive mediators such as platelet-derived growth factor (PDGF), transforming growth factor-β (TGF-β), vascular endothelial growth factor (VEGF), and stromal cell-derived factor-1 (SDF-1/CXCL12). During the inflammatory phase, neutrophils and macrophages orchestrate pathogen clearance, reactive oxygen species (ROS) production, and cytokine-mediated immune activation through Toll-like receptor (TLR) and NOD-like receptor (NLR) signaling pathways. The switch in macrophages from a pro-inflammatory M1 phenotype to a pro-resolving M2 phenotype constitutes a critical switch that allows for progression toward tissue repair. The proliferative phase is characterized by keratinocyte migration, fibroblast activation and differentiation into α-smooth muscle actin (α-SMA) expressing myofibroblasts, extracellular matrix deposition, angiogenesis, and reepithelialization. These processes are under the influence of growth factors, such as epidermal growth factor (EGF), keratinocyte growth factor/fibroblast growth factor-7 (KGF/FGF-7), hepatocyte growth factor (HGF), VEGF and TGF-β1. During the remodeling phase, extracellular matrix maturation is achieved through collagen III replacement by collagen I, matrix metalloproteinase (MMP)-mediated matrix turnover, tissue inhibitor of metalloproteinase (TIMP) regulation, collagen cross-linking and apoptosis of myofibroblasts, which ultimately restores tissue tensile strength and structural integrity.

Figure 1B, corresponding to the lower panel, highlights integrated biological mechanisms that govern successful tissue repair, whereas the chronic wound inset summarizes the principal pathological derailments associated with non-healing wounds, including persistent inflammation, impaired M1-to-M2 macrophage transition, fibroblast dysfunction, extracellular matrix disorganization and defective angiogenesis. Collectively, these alterations arrest healing within the inflammatory phase and constitute a central hallmark of chronic wound pathophysiology.

### 2.2. Transition from Acute to Chronic Wound

The pathological shifts that convert an acute wound into a chronic one—excess protease activity, hypoxia, and oxidative imbalance—are also the conditions that inactivate endogenous and applied AMPs, and therefore define the microenvironment that any peptide-based therapy must be engineered to withstand. Chronic wounds develop when tightly regulated healing processes become dysregulated and fail to progress beyond the inflammatory stage [8,23,24]. Persistent inflammation is considered the central pathological hallmark of wound chronicity and is closely associated with excessive protease activity, senescent cell accumulation, vascular dysfunction, microbial persistence, and oxidative imbalance [16,29,46].

**Persistent Inflammation**

Unlike acute wounds, chronic wounds exhibit prolonged infiltration of neutrophils and pro-inflammatory macrophages accompanied by sustained expression of TNF-α, IL-1β, IL-6, and other inflammatory mediators [23,29,47]. Excessive neutrophil activation results in continuous ROS production and protease release, leading to collateral tissue destruction and extracellular matrix degradation [31,32,48]. Importantly, unresolved inflammation promotes biofilm persistence by damaging local tissue architecture and impairing effective immune clearance [6,7,49].

**Protease-Rich Environment**

Chronic wounds are characterized by elevated levels of matrix metalloproteinases (MMPs), neutrophil elastase, cathepsins, and bacterial proteases [16,43,50]. Excessive proteolytic activity degrades extracellular matrix proteins, growth factors, cytokines, and endogenous antimicrobial peptides, thereby impairing tissue regeneration and innate antimicrobial defense [16,43,50]. Protease-mediated degradation of AMPs represents a particularly important translational barrier for peptide-based therapeutics [17,19].

**Hypoxia**

Microvascular dysfunction, edema, impaired perfusion, and elevated metabolic demands create profound hypoxia within chronic wounds [5,40,51]. Although transient hypoxia promotes physiological angiogenesis during acute healing, prolonged hypoxia disrupts fibroblast activity, collagen deposition, keratinocyte migration, and immune cell function [40,51,52]. Hypoxic microenvironments additionally favor biofilm persistence and reduce antibiotic susceptibility by inducing slow-growing bacterial phenotypes [7,53].

**ROS Imbalance**

Controlled reactive oxygen species (ROS) production is necessary for antimicrobial defense and intracellular signaling during normal healing [23,54]. However, chronic wounds exhibit excessive and sustained ROS accumulation derived from activated neutrophils, dysfunctional mitochondria, ischemia–reperfusion injury, and microbial metabolism [29,54,55]. Excessive oxidative stress damages DNA, proteins, lipids, and stem-cell populations while further amplifying inflammation and senescence pathways [54,55,56]. Biofilms themselves contribute to oxidative imbalance by stimulating persistent neutrophil recruitment and inflammatory activation [6,7,49].

**Impaired Angiogenesis**

Chronic wounds demonstrate defective angiogenic responses despite elevated VEGF expression [40,54]. Persistent inflammation, proteolytic degradation of angiogenic mediators, endothelial dysfunction, and hyperglycemia impair neovascularization and tissue oxygenation [4,5,40]. Reduced vascularization compromises nutrient delivery, immune surveillance, and tissue regeneration, thereby favoring microbial colonization and chronic infection [6,12,49].

**Cellular Senescence**

Senescent fibroblasts, keratinocytes, endothelial cells, and immune cells accumulate within chronic wounds and contribute to healing failure through the senescence-associated secretory phenotype (SASP) [57,58]. Senescent cells secrete pro-inflammatory cytokines, matrix metalloproteinases, and ROS while exhibiting impaired proliferative and regenerative capacity [57,58,59]. Importantly, senescence promotes chronic microbial colonization and biofilm persistence through sustained inflammatory signaling and defective immune responses [12,57,58].

**Figure 2:**
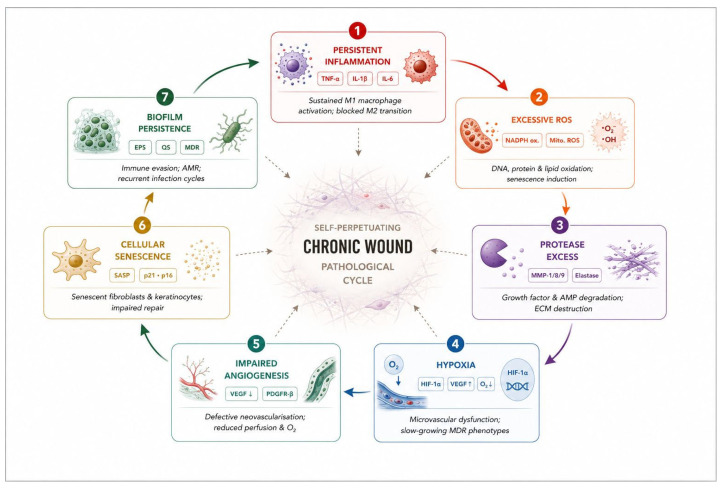
Pathological progression from acute to chronic wound cycle. The graphic illustrates the interconnected pathogenic pathways allowing progression from normal wound healing to chronic non-healing states. Chronic inflammation is induced and maintained through the long-lasting activation of pro-inflammatory M1 macrophages and defective switch to pro-resolving M2 phenotypes, resulting in persistent secretion of tumour necrosis factor-α (TNF-α), interleukin-1β (IL-1β) and interleukin-6 (IL-6). Excessive generation of reactive oxygen species (ROS), originating from NADPH oxidase activity and mitochondrial dysfunction, causes oxidative damage to lipids, proteins and DNA, thus promoting cellular dysfunction and senescence.

Figure 2 depicts protease activity, mainly mediated by matrix metalloproteinases (MMPs) and neutrophil elastase, whose increases induce degradation of the extracellular matrix (ECM) and deactivation of growth factors and antimicrobial peptides. Concurrently, microvascular dysfunction and tissue hypoxia impede oxygen transport and metabolic equilibrium, despite the activation of hypoxia-inducible factor-1α (HIF-1α)-dependent pathways. Limited angiogenesis limits neovascularization and tissue perfusion, which reduces the capacity to generate granulation tissue effectively and re-epithelize. Cellular senescence of fibroblasts and keratinocytes, associated with senescence-associated secretory phenotype (SASP) signaling and synthesis of cyclin-dependent kinase inhibitors p16 and p21, hinders tissue regeneration and perpetuates inflammatory signaling. The inherent durability of microbial biofilms allows immune evasion, recurrent cycles of infection, antibiotic resistance and persistent activation of inflammatory pathways through protection by EPS and microbial communication by quorum sensing (QS). These pathogenic stimuli establish a self-perpetuating biological network, in which each process amplifies adjacent mechanisms, thus perpetuating inflammation, extracellular matrix degradation, impaired vascularization and insufficient tissue repair. The circular arrangement of the image highlights the continuous reciprocal interactions that hinder the progression of physiological wound healing and sustain chronic wound disease. Abbreviations: AMR (antimicrobial resistance), AMP (antimicrobial peptide), ECM (extracellular matrix), EPS, HIF-1α (hypoxia-inducible factor-1α), IL (interleukin), MDR (multidrug resistant), MMP (matrix metalloproteinase), NADPH (nicotinamide adenine dinucleotide phosphate), QS (quorum sensing), ROS (reactive oxygen species), SASP (senescence-associated secretory phenotype), TNF-α (tumor necrosis factor-α), VEGF (vascular endothelial growth factor).

### 2.3. Biofilm Biology in Chronic Wounds

Biofilm architecture is the principal obstacle to AMP efficacy in chronic wounds, because the EPS matrix, quorum-sensing networks, and persister populations described below each represent a distinct barrier that AMP-enabled therapeutics are explicitly designed to overcome. Biofilms are highly structured polymicrobial microbial communities enclosed within a self-produced EPS matrix composed primarily of polysaccharides, proteins, extracellular DNA (eDNA), lipids, metabolites, and host-derived components [60,61,62,63]. Within chronic wounds, biofilms function as dynamic pathogenic ecosystems that profoundly alter host–pathogen interactions, immune responses, microbial metabolism, and antimicrobial susceptibility [6,7,12,47]. Unlike planktonic bacteria, biofilm-associated microorganisms exist within spatially heterogeneous microenvironments characterized by steep oxygen, nutrient, pH, and redox gradients that promote metabolic diversification, stress adaptation, and phenotypic tolerance [60,61,64]. These characteristics enable chronic wound biofilms to persist despite prolonged immune activation, antimicrobial exposure, and repeated debridement procedures.

**Biofilm Formation Stages**

Biofilm development generally proceeds through sequential stages, including reversible attachment, irreversible adhesion, microcolony formation, maturation, and dispersal [64,65]. Initial attachment is mediated by bacterial adhesins, pili, flagella, fimbriae, surface hydrophobic interactions, and host-derived extracellular matrix proteins exposed within the wound bed [64,65]. Damaged tissue surfaces enriched in fibrin, collagen, and fibronectin provide highly favorable substrates for microbial colonization. Following adhesion, bacterial proliferation and intercellular signaling promote microcolony formation and progressive EPS accumulation [66,67,68].

During maturation, microorganisms generate highly organized three-dimensional architectures containing nutrient gradients, water channels, anaerobic niches, and metabolically distinct subpopulations [60,61,64]. These mature biofilms create physical and biochemical diffusion barriers that impair penetration of antibiotics, antibodies, complement proteins, reactive oxygen species (ROS), and antimicrobial peptides [7,60,61]. Biofilm dispersal subsequently releases planktonic or biofilm-derived cells capable of colonizing adjacent tissue regions, seeding secondary biofilms, and initiating recurrent infection [47,64]. Importantly, dispersed biofilm cells may exhibit enhanced virulence and stress tolerance compared with conventional planktonic populations.

**EPS Matrix**

The EPS matrix represents one of the principal determinants of biofilm resilience and chronic persistence [60,61]. Beyond serving as a passive structural scaffold, EPS functions as a highly dynamic protective microenvironment that regulates nutrient retention, intercellular communication, osmotic balance, and stress adaptation [60]. EPS physically restricts antibiotic penetration, sequesters antimicrobial molecules, buffers environmental stress, neutralizes ROS, and protects bacteria from phagocytosis and oxidative killing [7,60,61].

Extracellular DNA (eDNA) constitutes a particularly important EPS component because it contributes to structural integrity, biofilm adhesion, cation sequestration, immune modulation, and horizontal gene transfer [66]. In chronic wound biofilms, eDNA additionally increases matrix viscosity and enhances tolerance to cationic antimicrobial peptides through electrostatic interactions [66]. Importantly, host-derived molecules such as fibrin, collagen fragments, plasma proteins, and necrotic cellular debris may become integrated into the EPS network, further strengthening biofilm stability and immune evasion [47].

**Quorum Sensing**

Quorum sensing (QS) enables bacterial populations to coordinate collective behavior through diffusible signaling molecules, including acyl-homoserine lactones, autoinducing peptides, and Pseudomonas quinolone signal molecules [67,68]. QS regulates biofilm maturation, EPS synthesis, virulence factor production, metabolic adaptation, stress responses, antimicrobial tolerance, and dispersal dynamics [67]. Within chronic wounds, QS signaling additionally modulates interspecies competition and cooperative metabolic interactions among polymicrobial communities.

Chronic wound pathogens such as *Pseudomonas aeruginosa* rely heavily on QS-mediated regulatory networks to coordinate biofilm maintenance, toxin production, iron acquisition, immune evasion, and persistence under hostile wound microenvironmental conditions [68,69]. Importantly, QS pathways have emerged as promising therapeutic targets, as disruption of bacterial communication may attenuate virulence without exerting the strong selective pressures typically associated with bactericidal antibiotics.

**Persister Cells**

Biofilms contain metabolically dormant persister cells capable of surviving otherwise lethal antimicrobial concentrations [70,71]. Unlike genetically resistant bacteria, persister cells exhibit transient phenotypic tolerance associated with reduced metabolic activity, stringent-response activation, toxin–antitoxin systems, ATP depletion, oxidative stress adaptation, and altered membrane physiology [70]. Because many conventional antibiotics preferentially target actively dividing cells, dormant persister populations remain largely unaffected during antimicrobial therapy.

Within chronic wounds, persister cells contribute substantially to recurrent infection, prolonged inflammation, and therapeutic failure [70,71]. Following antibiotic withdrawal, surviving persister cells can repopulate the wound and regenerate mature biofilms, thereby perpetuating chronic infection cycles. Increasing evidence additionally suggests that biofilm microenvironments facilitate the transition between persister phenotypes and genetically resistant bacterial populations.

**Polymicrobial Biofilms**

Chronic wound biofilms are frequently polymicrobial and involve complex interactions among bacteria, fungi, host cells, and wound-derived biochemical components [6,12,47]. These multispecies communities exhibit extensive cooperative and competitive interactions that enhance metabolic adaptability, nutrient utilization, antimicrobial tolerance, virulence, and immune evasion [72,73]. Spatial organization within polymicrobial biofilms allows microorganisms to occupy metabolically specialized niches while benefiting from shared protective mechanisms.

For example, *Pseudomonas aeruginosa* and *Staphylococcus aureus* frequently coexist within chronic wounds, where interspecies interactions modulate virulence expression, respiratory metabolism, toxin production, and antimicrobial susceptibility [72]. *P. aeruginosa* may induce small-colony variants of *S. aureus*, thereby promoting enhanced intracellular persistence and antibiotic tolerance [73]. Fungal organisms such as *Candida albicans* may further contribute to biofilm stability and inflammatory dysregulation within chronic wound environments.

**Host Immune Evasion**

Biofilms impair immune clearance through multiple mechanisms, including inhibition of phagocytosis, complement evasion, suppression of neutrophil killing, macrophage polarization dysregulation, and induction of dysfunctional chronic inflammation [7,47,60]. EPS-mediated shielding limits immune-cell access to bacterial aggregates, while QS-regulated virulence factors interfere with innate immune signaling and leukocyte function [67,68].

Persistent neutrophil recruitment around biofilms contributes to “frustrated phagocytosis,” a phenomenon in which immune cells release ROS, proteases, neutrophil extracellular traps (NETs), and inflammatory mediators without successfully eradicating the microbial community [29,31,32]. This process amplifies collateral tissue destruction, extracellular matrix degradation, oxidative stress, and wound chronicity while simultaneously favoring continued biofilm persistence. Biofilms may additionally skew macrophage polarization toward dysfunctional inflammatory phenotypes associated with impaired resolution and defective tissue repair.

**Resistance Amplification**

Biofilms promote antimicrobial resistance and tolerance through multiple complementary mechanisms, including restricted drug penetration, horizontal gene transfer, metabolic heterogeneity, efflux-pump activation, stress-response signaling, persister-cell enrichment, and selective pressure-driven adaptation [7,11,51,70]. Importantly, biofilm-associated microorganisms may exhibit antimicrobial tolerance levels up to 100–800-fold greater than planktonic counterparts [7,51].

The chronic wound microenvironment further intensifies resistance development by exposing microorganisms to prolonged subtherapeutic antimicrobial concentrations, oxidative stress, hypoxia, and inflammatory mediators [47,51]. Enhanced horizontal gene transfer within densely packed microbial communities facilitates dissemination of resistance determinants through plasmids, transposons, bacteriophages, and extracellular DNA [66]. Collectively, these mechanisms transform chronic wound biofilms into highly adaptive reservoirs of antimicrobial tolerance and multidrug resistance that remain exceptionally difficult to eradicate using conventional therapies.

**Figure 3:**
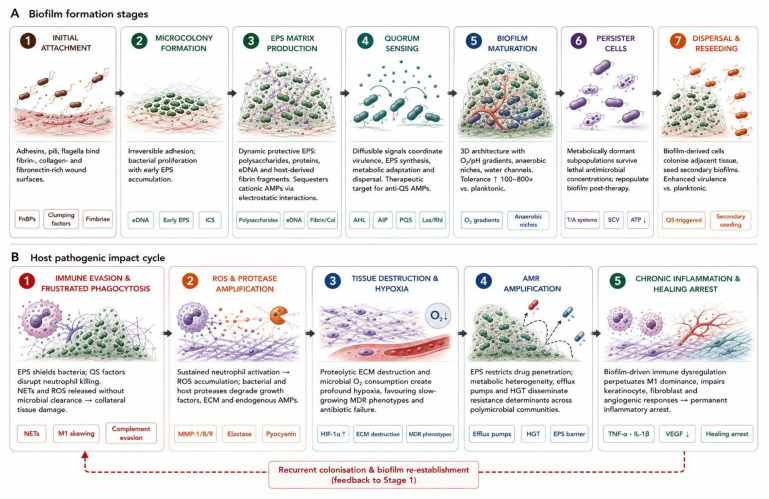
Biofilm-mediated chronic wound pathogenic cycle. (**A**) Biofilm formation stages. The seven stages of biofilm development: initial attachment of bacteria to wound surfaces, microcolony formation, EPS matrix production, quorum sensing, biofilm maturation (3D architecture with O_2_ gradients and 100–800× antibiotic tolerance), persister cell formation, and dispersal/reseeding that colonizes adjacent tissue. (**B**) Host pathogenic impact cycle. How biofilms damage the host across five steps: immune evasion and frustrated phagocytosis, ROS and protease amplification causing collateral tissue damage, tissue destruction and hypoxia, AMR amplification via restricted drug penetration and gene transfer, and chronic inflammation with healing arrest—feeding back to recurrent colonization and biofilm re-establishment. Abbreviations: AHL, acyl-homoserine lactone; AIP, autoinducing peptide; AMP, antimicrobial peptide; AMR, antimicrobial resistance; Col, collagen; eDNA, extracellular DNA; ECM, extracellular matrix; EPS, extracellular polymeric substance; FnBPs, fibronectin binding proteins; HGT, horizontal gene transfer; HIF-1α, hypoxia-inducible factor-1α; ICS, intercellular signalling; MDR, multidrug resistant; MMP, matrix metalloproteinase; NETs, neutrophil extracellular traps; PQS, Pseudomonas quinolone signal; QS, quorum sensing; ROS, reactive oxygen species; SCV, small colony variant; T/A, toxin-antitoxin; VEGF, vascular endothelial growth factor.

Figure 3 illustrates the sequential stages of biofilm development in chronic wounds and the associated host pathogenic feedback cycle responsible for wound chronicity. Panel A depicts the seven major stages of biofilm formation. Initial microbial attachment to fibrin-, collagen- and fibronectin-rich wound surfaces is mediated by bacterial adhesins, pili, fimbriae and fibronectin-binding proteins, and is accompanied by early EPS accumulation. EPS matrix production progresses to generate a highly dynamic protective microenvironment of polysaccharides, proteins, extracellular DNA (eDNA) and host-derived matrix components that together provide protection of bacteria from immune and antimicrobial attack. Quorum sensing (QS)-mediated signaling then coordinates virulence factor production, EPS synthesis, metabolic adaptation and biofilm dispersal. Maturation of the biofilm results in a complex three-dimensional architecture, with oxygen and pH gradients, anaerobic niches, water channels and pronounced antimicrobial tolerance. Survival is further enhanced by the formation of persister cells, metabolically dormant bacterial subpopulations capable of withstanding lethal antimicrobial exposure. Finally, dispersal and reseeding release biofilm-derived bacteria capable of colonizing adjacent tissue and establishing secondary biofilms with enhanced virulence.

Panel B summarizes the host pathogenic impact cycle driven by established biofilms. Biofilm-associated immune evasion and frustrated phagocytosis impair effective neutrophil-mediated clearance while promoting excessive release of neutrophil extracellular traps (NETs) and reactive oxygen species (ROS), thereby inducing collateral tissue injury.

Sustained ROS accumulation and protease amplification result in degradation of extracellular matrix (ECM) components, growth factors, and endogenous antimicrobial peptides, perpetuating tissue destruction and inflammatory signaling. Simultaneous microbial oxygen consumption and proteolytic ECM degradation lead to severe tissue hypoxia, favoring the growth of slow-multidrug resistant (MDR) phenotypes and decreasing antimicrobial efficacy. Biofilm architecture also plays a role in antimicrobial resistance (AMR), limiting drug penetration, metabolic heterogeneity, efflux pump activity and horizontal gene transfer (HGT).

These mechanisms sustain chronic inflammation; keep M1 macrophage dominance; impair keratinocyte, fibroblast and angiogenic responses; and ultimately lead to irreversible healing arrest. The lower feedback loop emphasizes repeated microbial colonization and reestablishment of biofilm, thus perpetuating the chronic wound condition.

### 2.4. Biofilm-Forming Pathogens and Their Translational Relevance in Chronic Wounds

The dominant chronic-wound pathogens differ in the persistence and resistance traits that determine which peptide chemistry and delivery system is most appropriate, as detailed for each organism below and summarized in Table 1. Chronic wounds harbor highly diverse and dynamic polymicrobial communities whose composition varies according to wound type, tissue oxygenation, host immune status, anatomical location, prior antimicrobial exposure, and local wound microenvironmental conditions [74,75,76]. Diabetic foot ulcers commonly contain mixed aerobic–anaerobic populations enriched in *Staphylococcus aureus*, *Pseudomonas aeruginosa*, *Enterococcus faecalis*, and anaerobic bacteria, whereas burn wounds and healthcare-associated wounds are frequently dominated by multidrug-resistant Gram-negative organisms, including *Acinetobacter baumannii* and *P. aeruginosa* [74,76,77]. Importantly, microbial pathogenicity in chronic wounds is determined less by planktonic bacterial burden and more by biofilm architecture, interspecies communication, metabolic heterogeneity, virulence regulation, and antimicrobial tolerance [7,47,60,61,64].

Within mature chronic wound biofilms, microorganisms undergo profound phenotypic adaptation characterized by quorum-sensing activation, oxidative-stress resistance, metabolic reprogramming, persister-cell formation, and enhanced immune evasion [67,68,69,70,71]. These adaptations perpetuate chronic inflammation, extracellular matrix degradation, impaired angiogenesis, and recurrent infection despite aggressive antimicrobial therapy [47,75]. Importantly, the pathogenicity of chronic wound microorganisms is closely linked to their biofilm-forming capacity, metabolic adaptability, immune-evasion mechanisms, and resistance phenotype rather than mere microbial presence.

**Methicillin-resistant *Staphylococcus aureus* (MRSA)**

Methicillin-resistant *Staphylococcus aureus* (MRSA) remains one of the most clinically important chronic wound pathogens because of its multidrug resistance, strong biofilm-forming capacity, and extensive repertoire of immune-evasion factors [78,79]. MRSA efficiently colonizes damaged tissue surfaces through adhesive surface proteins, including fibronectin-binding proteins and clumping factors, which facilitate attachment to fibrin-, collagen-, and fibronectin-rich wound beds [79,80]. During biofilm maturation, MRSA produces extracellular matrices enriched in polysaccharides, proteins, and extracellular DNA that impair antibiotic penetration, alter immune-cell function, and promote persistence [60,66,81].

Importantly, MRSA biofilms contain metabolically heterogeneous subpopulations, including small-colony variants and intracellular persisters, that contribute to prolonged survival, immune escape, and recurrent infection [81,82]. MRSA additionally secretes cytotoxins, hemolysins, proteases, and immune-modulating molecules capable of disrupting neutrophil function and amplifying inflammatory tissue damage [79,81]. From a translational perspective, MRSA represents a highly relevant target for antimicrobial peptides because many AMPs exert rapid membrane-disruptive activity capable of bypassing classical β-lactam resistance mechanisms mediated by the *mecA* gene and altered penicillin-binding proteins [13,14,15].

***Pseudomonas aeruginosa***

*Pseudomonas aeruginosa* is strongly associated with chronic wounds, diabetic ulcers, and burn infections because of its remarkable metabolic adaptability, intrinsic antimicrobial resistance, and highly coordinated quorum-sensing systems [68,83,84]. This pathogen forms structurally complex biofilms characterized by abundant EPS production, water-channel organization, anaerobic niches, and profound metabolic heterogeneity [60,61,64]. *P. aeruginosa* utilizes interconnected Las, Rhl, and Pseudomonas quinolone signaling pathways to regulate biofilm maturation, virulence-factor secretion, iron acquisition, stress adaptation, and dispersal dynamics [68,69,83].

A major contributor to *P. aeruginosa*-mediated tissue injury is the production of phenazines such as pyocyanin, which induce oxidative stress, mitochondrial dysfunction, neutrophil dysregulation, epithelial injury, and inflammatory persistence [83,85]. Within hypoxic chronic wound environments, *P. aeruginosa* can transition toward anaerobic respiration and slow-growth phenotypes associated with increased antimicrobial tolerance [49,83]. Furthermore, *P. aeruginosa* frequently dominates polymicrobial wound biofilms and modulates the behavior of coexisting organisms, including *S. aureus*, promoting small-colony variant formation and enhanced antibiotic tolerance [72,73].

Importantly, *P. aeruginosa* actively remodels the wound microenvironment by amplifying ROS accumulation, inflammatory signaling, protease activation, vascular dysfunction, and tissue hypoxia, thereby perpetuating wound chronicity [47,52,83]. These characteristics make anti-quorum-sensing peptides, ROS-responsive biomaterials, and EPS-disruptive AMP delivery systems particularly attractive therapeutic strategies for chronic *P. aeruginosa* wound infections.

***Enterococcus faecalis***

*Enterococcus faecalis* is increasingly recognized as an important opportunistic pathogen in chronic wound infections, particularly within polymicrobial diabetic foot ulcers and healthcare-associated wounds [76,86]. Its persistence is facilitated by strong biofilm-forming capacity, intrinsic tolerance to environmental stress, secretion of gelatinase and cytolysin virulence factors, and efficient horizontal gene transfer mechanisms [86,87]. Within polymicrobial communities, *E. faecalis* may cooperate metabolically with coexisting microorganisms, thereby enhancing collective biofilm stability and antimicrobial tolerance [86,87].

Of particular concern is the emergence of vancomycin-resistant enterococci (VRE), which substantially complicate chronic wound management because of limited therapeutic options and high transmissibility of resistance determinants [87]. Biofilm-associated *E. faecalis* additionally exhibits increased tolerance to oxidative stress and host immune defenses, contributing to persistent inflammatory activation and impaired tissue regeneration [86,87]. These characteristics support the development of combination therapeutic strategies integrating AMPs, antibiofilm biomaterials, and localized delivery systems capable of overcoming multidrug tolerance within polymicrobial wound environments.

***Acinetobacter baumannii***

*Acinetobacter baumannii* has emerged as a major pathogen in burn wounds, combat-related injuries, and intensive-care-associated wound infections because of its exceptional environmental resilience and multidrug-resistant phenotype [77,88,89]. This organism demonstrates remarkable desiccation tolerance and prolonged survival on abiotic hospital surfaces, facilitating nosocomial transmission and recurrent wound contamination [88,89]. In addition, *A. baumannii* forms persistent biofilms on both biological tissues and medical devices, contributing to chronic colonization and poor therapeutic response [88,90].

Clinically important strains frequently exhibit multidrug-resistant or extensively drug-resistant phenotypes associated with efflux pumps, carbapenemases, membrane modifications, and biofilm-mediated tolerance mechanisms [88,89,90]. *A. baumannii* infections are particularly problematic in military trauma, burn units, and intensive-care settings, where severe tissue injury, ischemia, and immune dysregulation create permissive conditions for persistent colonization [77,88]. The increasing prevalence of MDR/XDR *A. baumannii* further underscores the urgent need for alternative therapeutics, including AMP-loaded nanomaterials, membrane-active peptides, and multifunctional antibiofilm dressings capable of localized high-concentration delivery within infected wound beds.

## 3. Current Clinical Management of Biofilm-Infected Chronic Wounds and Unmet Translational Needs

### 3.1. Diagnosis and Clinical Suspicion of Biofilm-Associated Infection

Reliable recognition of biofilm-associated infection is the clinical entry point for AMP-based intervention, since the indirect signs below are what currently trigger escalation toward antibiofilm therapy. Biofilm-associated infection remains difficult to diagnose in routine clinical practice because mature biofilms are not directly visible during bedside examination and frequently evade conventional microbiological detection methods [91,92]. Consequently, current diagnostic approaches rely predominantly on indirect clinical indicators, including delayed healing, recurrent inflammation, excessive exudate, friable granulation tissue, malodor, recurrent slough formation after debridement, and poor response to antimicrobial therapy [91,92,93]. Chronic wounds are now recognized as dynamic polymicrobial ecosystems in which microbial interactions, biofilm maturation, and host immune dysregulation contribute substantially to wound chronicity [74,75,76].

The International Wound Infection Institute (IWII) consensus framework emphasizes evaluation along a wound infection continuum ranging from contamination and colonization to local, spreading, and systemic infection [91]. Similarly, the 2023 International Working Group on the Diabetic Foot (IWGDF) and Infectious Diseases Society of America (IDSA) guideline recommends diagnosing diabetic foot infection clinically rather than solely microbiologically and supports severity-based classification to guide treatment selection [92]. However, reliable bedside biofilm diagnostics remain limited, representing a major unmet clinical need.

### 3.2. Wound Bed Preparation and Debridement

Debridement is the step that makes effective AMP delivery possible, because physically disrupting the EPS matrix exposes biofilm-embedded organisms to subsequently applied peptides. Debridement remains the cornerstone of chronic wound biofilm management because physical disruption of the EPS matrix is necessary before antimicrobial therapies can achieve meaningful efficacy [93,94,95]. Surgical, sharp, mechanical, ultrasonic, hydrosurgical, enzymatic, and autolytic debridement techniques are routinely employed to remove necrotic tissue, reduce microbial burden, and destabilize mature biofilms [93,95].

Importantly, biofilms can rapidly reform following incomplete removal, often within 24–72 h, necessitating repeated or maintenance debridement strategies [65,93]. The Wound Healing Society (WHS) guidelines specifically emphasize that chronic wound biofilms contribute to marked antimicrobial tolerance and impaired healing unless the biofilm matrix is physically disrupted [93]. Debridement is therefore considered an essential preparatory step rather than a standalone therapy.

### 3.3. Topical Antiseptics and Antimicrobial Dressings

Conventional topical antiseptics define the efficacy-versus-toxicity benchmark that AMP-enabled dressings aim to improve upon by combining antimicrobial action with greater selectivity for host tissue. Topical antiseptics and antimicrobial dressings are widely used to reduce local bioburden and suppress biofilm re-establishment after debridement [94,95,96]. Commonly used agents include silver-containing dressings, iodophors, chlorhexidine, octenidine, polyhexamethylene biguanide/polyhexanide (PHMB), hypochlorous acid, and honey-based formulations [94,96].

Silver-containing dressings remain among the most frequently utilized antibiofilm wound therapies because of their broad-spectrum antimicrobial activity and ability to disrupt bacterial membranes and metabolic pathways [96]. However, concerns remain regarding cytotoxicity toward keratinocytes and fibroblasts, particularly with prolonged exposure [94]. Likewise, iodine-based formulations demonstrate strong antimicrobial efficacy but may impair tissue regeneration at higher concentrations [96].

Recent international consensus recommendations emphasize balancing antimicrobial efficacy with preservation of tissue viability and wound-healing capacity [94]. Nevertheless, current topical therapies often exhibit incomplete biofilm penetration, short retention time, reduced activity in protease-rich wound environments, and limited regenerative potential.

### 3.4. Systemic Antibiotics

The limited efficacy of systemic antibiotics against metabolically adapted biofilm-associated organisms is a central rationale for localized, AMP-based wound therapy. Systemic antibiotics remain essential for clinically infected chronic wounds presenting with cellulitis, abscess formation, spreading infection, osteomyelitis, or systemic inflammatory manifestations [5,92]. However, contemporary guidelines strongly discourage systemic antibiotic use for non-infected chronic wounds or simple colonization because unnecessary exposure promotes antimicrobial resistance and microbiome disruption [10,12,92].

### 3.5. Adjunctive and Advanced Therapies

Several adjunctive therapies target the same microenvironmental deficits—hypoxia, exudate, and biofilm burden—that multifunctional AMP-enabled biomaterials are increasingly designed to address in parallel. They have emerged to improve chronic wound healing and reduce biofilm persistence. Negative pressure wound therapy (NPWT), particularly NPWT with instillation, may reduce exudate accumulation, stimulate granulation tissue formation, improve perfusion, and mechanically disrupt biofilm architecture [93,97]. Additional approaches include ultrasound-assisted debridement, hydrosurgical systems, oxygen-based therapies, hyperbaric oxygen therapy, photodynamic therapy, shockwave therapy, and antibiofilm cleansing agents [95,97].

Oxygen-based therapies are particularly relevant because chronic wounds frequently exhibit severe tissue hypoxia that impairs angiogenesis, fibroblast activity, keratinocyte migration, and immune-cell function [49,50]. Sustained oxygenation systems have demonstrated promising regenerative and anti-inflammatory effects in experimental diabetic wound models [50]. Nevertheless, clinical evidence supporting many advanced wound-care interventions remains heterogeneous, and standardized treatment protocols are still lacking [95,97].

### 3.6. Unmet Needs and Rationale for AMP-Enabled Biomaterials

Despite major advances in wound care, current clinical management of biofilm-infected chronic wounds remains suboptimal. Mature biofilms rapidly reform after disruption, systemic antibiotics demonstrate poor efficacy against metabolically adapted biofilm-associated microorganisms, and many topical antiseptics exhibit limited selectivity or regenerative support [47,51,94]. In addition, chronic wounds represent highly hostile microenvironments characterized by excessive protease activity, persistent inflammation, oxidative stress, hypoxia, senescence, vascular dysfunction, and polymicrobial microbial persistence [16,46,47,48,49,50,51,52,53,54,55,56,57,58,59].

These limitations have generated increasing interest in antimicrobial peptides (AMPs) as multifunctional therapeutic agents capable of simultaneously targeting microbial infection and impaired tissue regeneration [9,13,14,15]. Unlike conventional antibiotics, AMPs frequently combine membrane-disruptive antimicrobial activity with antibiofilm effects, quorum-sensing interference, immune modulation, angiogenic stimulation, and wound-healing support [13,14,15].

However, free AMPs remain vulnerable to enzymatic degradation, rapid clearance, salt sensitivity, and protease-rich wound environments [16,17]. Consequently, advanced biomaterial-based delivery systems, including hydrogels, electrospun nanofibers, nanoparticles, scaffolds, and multifunctional smart dressings, are increasingly being developed to improve peptide stability, controlled release, biofilm penetration, and local therapeutic efficacy [18,19]. These multifunctional AMP-enabled biomaterials may therefore represent a promising next-generation strategy capable of simultaneously addressing infection control, biofilm disruption, immune dysregulation, and impaired tissue regeneration.

## 4. Antimicrobial Peptides as Multifunctional Antibiofilm and Regenerative Agents

Antimicrobial peptides (AMPs) are evolutionarily conserved effector molecules of innate immunity that exhibit broad-spectrum antimicrobial activity together with immunomodulatory, antibiofilm, and regenerative functions [9,13,14,15]. In contrast to conventional antibiotics that primarily target discrete intracellular pathways, AMPs exert rapid multimodal effects involving membrane destabilization, quorum-sensing interference, biofilm disruption, immune regulation, and wound-healing promotion [17,18,19]. These multifunctional properties are particularly attractive for chronic wound therapy, where persistent biofilms coexist with dysregulated inflammation, oxidative stress, hypoxia, vascular dysfunction, and impaired tissue regeneration [46,47,59].

### 4.1. Structural and Physicochemical Features of AMPs

Most AMPs are short peptides composed of approximately 10–50 amino acids and are characterized by cationicity, amphipathicity, and structural diversity [13,14,15]. Positive net charge, typically resulting from lysine- and arginine-rich sequences, facilitates electrostatic interaction with negatively charged bacterial membranes, extracellular DNA, lipopolysaccharides, and biofilm matrix components [98,99,100,101]. This electrostatic selectivity partly explains why AMPs preferentially target microbial membranes while exhibiting lower affinity for cholesterol-rich mammalian cell membranes [101].

Amphipathicity represents another defining physicochemical feature of AMPs. Spatial segregation of hydrophobic and hydrophilic domains enables insertion into phospholipid bilayers and destabilization of membrane architecture [100,102]. Hydrophobicity additionally influences membrane penetration, peptide aggregation, biofilm interaction, and cytotoxicity, making careful optimization essential for translational applications [17,102].

Structurally, AMPs are commonly classified into α-helical, β-sheet, cyclic, and extended peptides [14,15,100]. α-Helical peptides such as LL-37 and magainins are among the best-characterized AMP families because of their potent membrane-disruptive activity and broad-spectrum antimicrobial effects [100,103]. β-Sheet peptides, including defensins, contain disulfide bonds that enhance conformational stability and protease resistance [104]. Cyclic peptides demonstrate increased structural rigidity, prolonged biological half-life, and improved stability in protease-rich chronic wound environments [17,105]. In recent years, synthetic ultrashort peptides have emerged as attractive therapeutic candidates because of their lower manufacturing costs, tunable physicochemical properties, reduced immunogenicity, and easier scalability for clinical translation [106].

Importantly, AMP structure directly influences antimicrobial potency, antibiofilm efficacy, protease susceptibility, host–cell interactions, immunomodulatory activity, and compatibility with biomaterial delivery systems [17,18,19]. Consequently, rational peptide engineering increasingly focuses on balancing antimicrobial efficacy with regenerative functionality and biocompatibility.

### 4.2. Mechanisms of Antimicrobial Action

Unlike conventional antibiotics that frequently target specific metabolic pathways, AMPs exert rapid and multifactorial antimicrobial activity [100,101]. Membrane disruption represents the most extensively characterized mechanism and is initiated by electrostatic attraction between cationic peptides and negatively charged bacterial membranes [100]. Following membrane binding, peptides destabilize lipid organization, increase membrane permeability, and induce leakage of intracellular contents, ultimately resulting in microbial death [101,102].

Several mechanistic models have been proposed to explain AMP-mediated membrane disruption. In the barrel-stave model, peptides insert perpendicularly into lipid bilayers and form transmembrane pore-like structures [100,107]. In the toroidal pore model, peptides induce membrane curvature, generating pores lined by both phospholipids and peptides [107]. The carpet model involves peptide accumulation on the membrane surface followed by detergent-like membrane disintegration without discrete pore formation [100,107]. Importantly, many AMPs likely operate through multiple overlapping mechanisms depending on peptide concentration, membrane composition, and environmental conditions [102].

Beyond membrane disruption, numerous AMPs exert intracellular antimicrobial effects following membrane translocation [108]. Intracellular targets include DNA, RNA, ribosomes, ATP-generating pathways, chaperone proteins, and metabolic enzymes [108,109]. Certain peptides inhibit nucleic acid synthesis, interfere with protein translation, or disrupt intracellular homeostasis independently of direct membrane lysis [109].

Several AMPs additionally induce oxidative and metabolic stress within microbial cells. AMP exposure may stimulate excessive reactive oxygen species (ROS) generation, membrane lipid oxidation, mitochondrial dysfunction in fungi, and metabolic collapse, thereby contributing to bacterial killing and biofilm destabilization [110,111]. These mechanisms may be particularly relevant in metabolically heterogeneous chronic wound biofilms that exhibit limited susceptibility to conventional antibiotics [51,70].

Importantly, AMPs also demonstrate anti-virulence activity independent of direct bactericidal effects [67,68,69]. Numerous peptides interfere with quorum sensing, toxin secretion, bacterial motility, virulence factor production, and extracellular matrix synthesis, thereby attenuating pathogenicity while potentially reducing selective pressure for resistance development [67,68].

### 4.3. AMP Interactions with Biofilm Biology

AMP interactions with biofilms extend beyond simple bacterial killing and involve complex mechanisms targeting biofilm formation, maturation, persistence, and host–pathogen interactions [13,17,18]. Because chronic wound biofilms represent highly organized and metabolically heterogeneous ecosystems, effective therapeutic strategies must overcome extracellular matrix barriers, dormant bacterial populations, quorum-sensing networks, and dysfunctional inflammatory responses simultaneously [47,59,65].

A recurring source of imprecision in the antibiofilm literature, and one that this review seeks to avoid, is the interchangeable use of the terms “antimicrobial” and “antibiofilm”. The two describe mechanistically distinct activities and are quantified by different metrics. Conventional antimicrobial potency is expressed as the minimum inhibitory concentration (MIC) or minimum bactericidal concentration (MBC), both determined against free-floating planktonic cells. These values systematically underestimate the concentration required to act on biofilm-embedded organisms, which may tolerate antimicrobials at levels up to several hundred-fold higher than their planktonic counterparts [7,51]. Biofilm-directed efficacy is instead captured by the minimum biofilm inhibitory concentration (MBIC) and, more stringently, the minimum biofilm eradication concentration (MBEC), with the latter quantifying the concentration needed to eliminate pre-formed mature biofilms [112]. Throughout this review, antimicrobial activity therefore refers to direct killing of planktonic or dispersed cells, whereas antibiofilm activity denotes the additional capacity to prevent biofilm formation, penetrate or destabilize the established EPS matrix, and eradicate sessile and persister subpopulations. A peptide with a low planktonic MIC does not necessarily exhibit a correspondingly low MBEC, and this divergence is one of the principal reasons why in vitro antimicrobial promise frequently fails to translate into biofilm eradication in chronic wounds [7,17].

Whereas Section 2.3 characterized these biofilm processes from the perspective of microbial pathogenesis, the present section focuses specifically on the molecular mechanisms by which AMPs counteract each of them, cross-referencing that biology rather than restating it.

#### 4.3.1. Prevention of Adhesion

Initial microbial attachment represents a critical early step in biofilm formation [64,65]. Several AMPs inhibit bacterial adhesion by interfering with adhesin-mediated attachment, altering bacterial surface hydrophobicity, and disrupting interactions between microorganisms and extracellular matrix proteins [113]. Some peptides additionally reduce conditioning-film formation on biomaterial surfaces, thereby limiting subsequent microbial colonization [114]. Prevention of early attachment is particularly important in chronic wounds, where biofilms rapidly reform following debridement and antimicrobial treatment [91,97].

#### 4.3.2. EPS Destabilization and Matrix Penetration

The EPS matrix constitutes one of the major barriers limiting antimicrobial penetration into mature biofilms [60,61]. AMPs may destabilize EPS architecture through electrostatic interactions with negatively charged polysaccharides and extracellular DNA, thereby increasing matrix permeability and structural instability [60,66]. Certain peptides exhibit direct DNA-binding activity that weakens biofilm integrity and interferes with horizontal gene transfer [66]. Enhanced matrix penetration not only improves direct bacterial killing but may also potentiate the efficacy of co-administered antibiotics or antiseptics [17,19].

The efficiency with which an AMP reaches biofilm-embedded cells is governed not only by its intrinsic activity but by the biophysics of transport through the EPS matrix. Mature biofilms are interlaced with fluid-filled channels that permit convective and diffusive solute movement, yet the surrounding matrix is densely anionic, owing to acidic exopolysaccharides such as alginate, extracellular DNA, and negatively charged proteins [60,61,66]. Cationic AMPs are consequently subject to strong electrostatic retardation: the same positive net charge that drives selective binding to anionic bacterial membranes also promotes sequestration within the matrix, reducing the fraction of peptide that reaches deeper, metabolically dormant cells [60,66]. Peptide amphipathicity introduces a second constraint, since highly amphipathic sequences partition avidly into the first hydrophobic and polyanionic structures they encounter, further limiting penetration depth. The practical implication is a trade-off rarely made explicit in formulation studies: increasing cationic charge and amphipathicity enhances membrane activity but can impair diffusion through the EPS, so net charge, charge density, and hydrophobic moment should be optimized for matrix transport rather than for planktonic potency alone [17,99].

A complementary strategy to overcome matrix-limited penetration is the deliberate combination of AMPs with matrix-degrading enzymes. Glycoside hydrolases such as dispersin B, which depolymerizes the poly-β-1,6-N-acetylglucosamine (PNAG) that cements many staphylococcal and Gram-negative biofilms, together with DNases that cleave structural extracellular DNA, can mechanically open the EPS and expose embedded cells to subsequent peptide action [21,22,115]. Pre-treatment or co-delivery of such enzymes lowers the effective antibiofilm concentration of co-administered antimicrobials and is increasingly regarded as a rational adjunct to AMP-based therapy. Co-formulation within a single biomaterial platform (Section 6) is particularly attractive, as it allows sequential or simultaneous presentation of a matrix-disrupting enzyme and a membrane-active peptide at the wound interface, converting the EPS from a protective barrier into an accessible target.

#### 4.3.3. Quorum-Sensing Inhibition

Building directly on the quorum-sensing biology established in Section 2.3, several AMPs interfere with QS signaling pathways, including the Las, Rhl, and agr systems, thereby suppressing toxin production, EPS synthesis, bacterial motility, and virulence factor secretion [67,68,69].

In *Pseudomonas aeruginosa*, QS inhibition may reduce pyocyanin production, impair biofilm maturation, and attenuate inflammatory tissue injury [68,85]. Importantly, anti-QS strategies may reduce pathogenicity without exerting strong bactericidal selective pressure, potentially limiting resistance emergence [67].

#### 4.3.4. Persister-Cell Targeting

As detailed in Section 2.3, biofilms harbor metabolically dormant persister cells that survive conventional antibiotics precisely because antimicrobial efficacy typically depends on active bacterial metabolism. In contrast, membrane-targeting AMPs may retain activity against these dormant populations independently of metabolic state [70,71,108].

Certain peptides additionally disrupt ATP homeostasis, membrane integrity, and bacterial stress-response pathways, thereby enhancing eradication of persistent biofilm-associated microorganisms [70,71].

#### 4.3.5. Biofilm Dispersal

Some AMPs actively induce biofilm dispersal through matrix destabilization, signaling interference, or modulation of intracellular cyclic-di-GMP pathways [116]. Biofilm dispersal increases susceptibility of released microorganisms to host immune clearance and antimicrobial therapy [116,117]. However, uncontrolled dispersal may theoretically facilitate dissemination and recurrent infection, emphasizing the importance of combining dispersal-promoting peptides with effective antimicrobial or immune-targeting strategies [117,118].

#### 4.3.6. Immunomodulatory Antibiofilm Effects

Persistent inflammation surrounding chronic wound biofilms frequently causes collateral tissue destruction while failing to eradicate microbial communities [23,29,47]. AMPs may restore effective host defense by modulating macrophage polarization, neutrophil recruitment, cytokine production, and innate immune signaling [14,15,33]. Several peptides suppress excessive TNF-α and IL-1β production while simultaneously enhancing microbial clearance and tissue repair [14,15]. This dual antimicrobial-immunomodulatory activity fundamentally distinguishes AMPs from conventional antibiotics and may be particularly advantageous in chronic wounds characterized by dysfunctional inflammatory responses and impaired immune surveillance [46,59].

### 4.4. Regenerative and Immunomodulatory Effects

In addition to their antimicrobial activity, many AMPs directly participate in physiological wound healing and tissue regeneration [9,14,15]. This multifunctionality represents one of the major advantages of AMPs over conventional antibiotics and provides a strong rationale for their application in chronic wound therapy.

AMPs regulate macrophage polarization by promoting the transition from pro-inflammatory M1-like phenotypes toward reparative M2-like states [20,33,34]. This phenotypic shift contributes to inflammation resolution, reduced tissue injury, enhanced angiogenesis, and activation of tissue-repair pathways [33,34]. Several peptides additionally suppress excessive production of TNF-α, IL-1β, and IL-6 while stimulating anti-inflammatory cytokines and regenerative signaling cascades [14,15].

Many AMPs exhibit pro-angiogenic activity through stimulation of endothelial-cell migration, proliferation, and VEGF-associated signaling pathways [14,40]. Enhanced angiogenesis improves tissue oxygenation, nutrient delivery, immune-cell trafficking, and metabolic support within ischemic chronic wounds [40,46]. These effects may be particularly important in diabetic wounds characterized by severe vascular dysfunction and hypoxia [4,5,49].

Re-epithelialization is another critical AMP-regulated process. Several peptides stimulate keratinocyte proliferation and migration through activation of EGFR-related signaling pathways, cytoskeletal remodeling, and integrin-mediated interactions [14,36,37]. Accelerated keratinocyte migration contributes directly to wound closure and restoration of epithelial barrier integrity [37].

AMPs also influence fibroblast behavior and extracellular matrix remodeling [9,15]. Certain peptides promote fibroblast proliferation, collagen synthesis, granulation tissue formation, and ECM deposition while simultaneously modulating matrix metalloproteinase activity and scar remodeling [38,43]. These regenerative effects may improve tissue architecture while limiting chronic tissue degeneration and defective remodeling [42,43,44].

Importantly, AMPs additionally contribute to restoration of immunological homeostasis within chronic wounds by regulating innate immunity, host–microbiome interactions, and inflammatory signaling pathways [12,15]. Such multifunctional properties support their potential as integrated therapeutic agents capable of simultaneously targeting infection, inflammation, and impaired tissue regeneration.

**Figure 4:**
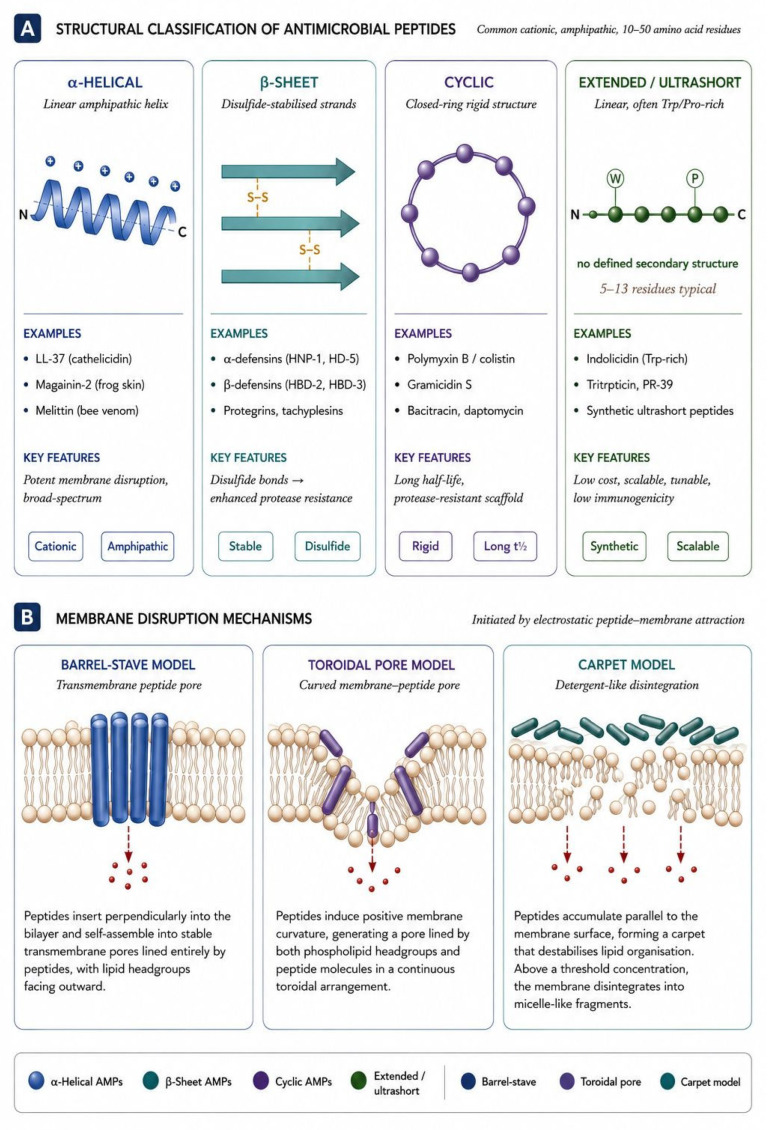
Antimicrobial peptides: structural classes and membrane disruption mechanisms. (**A**) Structural classification of antimicrobial peptides. The four structural classes of antimicrobial peptides (typically cationic, amphipathic, 10–50 residues): α-helical (e.g., LL-37, magainin-2), β-sheet with disulfide bonds (α- and β-defensins), cyclic closed-ring (polymyxin B, gramicidin S), and extended/ultrashort Trp/Pro-rich peptides (indolicidin), each with representative examples and key features. (**B**) Membrane disruption mechanisms. Three models of peptide-induced membrane disruption, initiated by electrostatic peptide–membrane attraction: the barrel-stave model (peptides insert to form transmembrane pores), the toroidal pore model (peptides bend the membrane into a curved pore lined by lipids and peptides), and the carpet model (peptides accumulate on the surface and disintegrate the membrane into micelle-like fragments). Abbreviations: AMP, antimicrobial peptide; HBD, human β-defensin; HD, human α-defensin; HNP, human neutrophil peptide; LL-37, human cathelicidin; PR-39, proline–arginine-rich peptide-39; t½, half-life; Trp, tryptophan; Pro, proline.

Figure 4A illustrates antimicrobial peptides (AMPs) as short cationic amphipathic molecules, typically consisting of 10–50 amino acid residues, classified into four principal structural families. α-Helical peptides (e.g., LL-37, magainin-2, melittin) adopt amphipathic helices upon membrane interaction and mediate potent broad-spectrum membrane disruption. β-sheet peptides (e.g., α/β-defensins, protegrins, tachyplesins) are stabilized by intramolecular disulfide bonds which confer enhanced structural stability and protease resistance. Cyclic peptides (e.g., polymyxin B, gramicidin S, daptomycin) possess a closed macrocyclic backbone associated with conformational rigidity and prolonged biological half-life. Extended and ultrashort peptides (e.g., indolicidin, tritrpticin, synthetic ultrashort peptides) lack a defined secondary structure and offer scalable synthesis, tunable physicochemical properties, and potentially reduced immunogenicity.

Figure 4B shows how AMP-mediated bacterial killing is initiated by electrostatic attraction between positively charged peptides and negatively charged microbial membranes, followed by membrane destabilization through the three classical mechanistic models (barrel-stave, toroidal-pore, and carpet) detailed in Section 4.2; as noted there, individual AMPs frequently act through partially overlapping mechanisms that depend on peptide concentration, membrane composition, ionic conditions, and the local microenvironment.

### 4.5. Translational Relevance of AMPs in Chronic Wounds

The multifunctional nature of AMPs positions them as highly attractive therapeutic candidates for chronic wound management [13,14,15,16,17,18,19]. Unlike conventional antibiotics, AMPs combine broad-spectrum antimicrobial activity with antibiofilm effects, immune modulation, anti-virulence properties, and regenerative support [14,15]. Their ability to disrupt extracellular polymeric matrices, interfere with quorum sensing, target persister populations, and simultaneously promote wound healing directly addresses several mechanisms underlying chronic wound persistence [17,18,19].

Nevertheless, major translational barriers continue to limit clinical implementation of free peptide therapies [16,17]. Chronic wounds contain highly proteolytic microenvironments capable of rapidly degrading peptides and reducing therapeutic efficacy [16,48]. Additional limitations include short biological half-life, poor wound-site retention, salt sensitivity, potential cytotoxicity, and manufacturing costs [17,19]. Furthermore, highly exudative and polymicrobial wound environments complicate sustained local peptide delivery and therapeutic stability [47,59].

These challenges have stimulated increasing interest in advanced biomaterial-based delivery systems capable of protecting AMPs from degradation, improving local retention, enabling controlled release, enhancing biofilm penetration, and simultaneously supporting tissue regeneration [18,19]. Consequently, integration of AMPs with hydrogels, nanofibers, nanoparticles, scaffolds, and multifunctional smart dressings has emerged as one of the most promising translational directions in chronic wound therapeutics.

## 5. Translational Barriers Limiting AMP-Based Therapy in Chronic Biofilm-Infected Wounds

Despite the remarkable antimicrobial, antibiofilm, immunomodulatory, and regenerative potential of antimicrobial peptides (AMPs), successful clinical translation remains limited [13,14,15,16,17,18,19]. Chronic wounds represent highly hostile pathological environments characterized by excessive protease activity, oxidative stress, hypoxia, polymicrobial biofilms, abnormal pH, ischemia, and persistent inflammation, all of which substantially compromise peptide stability and therapeutic efficacy [16,46,47,48,49,50,51,52,53,54,55,56,57,58,59]. Furthermore, translational development is complicated by toxicity concerns, pharmacokinetic limitations, manufacturing costs, lack of standardized experimental models, and regulatory uncertainty [17,18,119]. Consequently, although numerous AMPs demonstrate promising in vitro antibiofilm activity, relatively few have advanced successfully into late-stage clinical wound applications [17,119].

### 5.1. Biochemical Instability of AMPs in Chronic Wound Microenvironments

One of the principal barriers limiting AMP therapy is rapid biochemical degradation within chronic wound environments [16,17]. Chronic wound exudates contain elevated concentrations of host-derived and bacterial proteases, including matrix metalloproteinases (MMPs), neutrophil elastase, cathepsins, and microbial proteases capable of degrading therapeutic peptides before adequate antimicrobial activity can be achieved [16,43,48]. Persistent inflammation and excessive neutrophil activation further amplify proteolytic burden and oxidative stress, accelerating peptide fragmentation and functional inactivation [29,47,52,53].

Biofilm-associated microorganisms additionally contribute to peptide instability through secretion of extracellular proteases and matrix-associated enzymes [47,60,61,64,65,66,120,121]. *Pseudomonas aeruginosa*, for example, produces elastases and alkaline proteases capable of degrading host-defense peptides and impairing innate immune responses [68,85]. Moreover, EPS components may sequester cationic peptides through electrostatic interactions, thereby reducing effective antimicrobial concentrations within deeper biofilm layers [60,61,120].

Physicochemical conditions present in chronic wounds further complicate AMP stability. Elevated ionic strength, altered pH, oxidative stress, and wound-fluid composition may interfere with electrostatic peptide–membrane interactions and reduce antimicrobial activity [17,99,100,101,102,103,119]. Salt sensitivity is particularly relevant because many cationic peptides exhibit reduced efficacy in physiological ionic environments rich in sodium, calcium, and magnesium ions [102,103]. Similarly, oxidative stress may induce peptide oxidation and conformational instability, thereby impairing antimicrobial potency and regenerative activity [52,53].

Collectively, these factors substantially limit the therapeutic performance of free AMPs in chronic wound settings and strongly support the development of protective delivery systems capable of enhancing peptide stability and bioavailability [18,19,119].

### 5.2. Chemical and Structural Engineering Strategies to Overcome AMP Instability and Toxicity

The biochemical fragility described in Section 5.1 is increasingly regarded not as an intrinsic limitation but as a tractable design problem, and most contemporary translational AMP research now focuses on chemical and structural engineering rather than on the discovery of additional native sequences [17,108,109]. A recurring principle, however, is that no single modification simultaneously optimizes antimicrobial potency, proteolytic stability, and host–cell selectivity; each strategy typically improves one property while compromising another, and this trade-off—rather than insufficient potency alone—largely explains why relatively few engineered peptides have advanced to late-stage chronic wound applications [17,99].

#### 5.2.1. Stereochemical Modification: D-Amino Acids

Because host and bacterial proteases recognize L-stereochemistry, substitution of L- with D-residues is among the most direct routes to proteolytic stability. Full enantiomeric substitution of the wasp-venom peptide polybia-MPI produced an all-D analogue that resisted trypsin and chymotrypsin, retained or slightly improved antibacterial potency, and exhibited reduced hemolysis, indicating that its membrane-disruptive target is not chirality-dependent [122]. The same study, however, exposed the central trade-off: the partially substituted D-lysine analogue gained stability but lost antimicrobial activity through disruption of its α-helical fold [122]. Comparable protease resistance accompanied by partial activity loss was reported for the all-D enantiomer of polybia-CP [123]. The limitation becomes most apparent in vivo: among D- and unnatural-amino-acid derivatives of the cationic peptide Pep05, an extensively D-substituted analogue combined strong stability with only marginal in vivo activity and increased toxicity, whereas incorporation of a single N-terminal α-aminoisobutyric acid (Aib) residue conferred both plasma stability and improved in vivo efficacy [124]. These findings demonstrate that in vitro stability does not reliably predict therapeutic performance.

#### 5.2.2. Cyclization and Conformational Constraint

Constraining peptide conformation reduces the accessibility of proteolytic cleavage sites while pre-organizing the bioactive structure. Head-to-tail and side-chain cyclization increase conformational rigidity and biological half-life and underlie the proteolytic resilience of naturally constrained scaffolds such as the disulfide-stabilized defensins and cyclic peptides [105]. In a directly comparative study of arginine-rich peptides, both cyclization and D-enantiomerization of the R4F4 backbone markedly enhanced stability against trypsin and serum proteases while preserving, or even improving, antibacterial activity [125]. Related helix-constraining approaches, including hydrocarbon stapling, similarly improve protease resistance by stabilizing the active α-helix; however, excessive helix stabilization may reduce cell selectivity and increase hemolytic activity, so conformational constraint must be balanced against host–cell toxicity [99].

#### 5.2.3. Terminal Modification, Lipidation, and PEGylation

Terminal modifications such as N-acetylation and C-amidation shield peptide termini from exopeptidases and, in the case of amidation, increase net cationic charge [108,109]. Conjugation of a fatty-acyl chain (lipidation) augments hydrophobic partitioning into microbial membranes and can substantially enhance potency; within the R4F4 series, an N-terminal C16 palmitoyl lipopeptide retained strong antibacterial activity together with improved proteolytic stability [125]. PEGylation illustrates the central compromise of the field particularly clearly: covalent attachment of polyethylene glycol prolongs circulation, reduces renal clearance, and lowers immunogenicity and cytotoxicity, but frequently diminishes antimicrobial potency through steric interference with membrane insertion [17]. Nonetheless, judicious design can preserve activity, as N-terminal PEGylation of the proline-rich peptide Bac7(1–35) reduced renal clearance and broadened biodistribution while retaining both antibacterial activity and bacterial cell-penetration capacity, with cleavable ester linkers further enabling local release of the active peptide [126].

#### 5.2.4. Peptidomimetics: Peptoids and Foldamers

A more radical strategy replaces the proteolytically vulnerable peptide backbone altogether. Peptoids (oligomers of N-substituted glycines) relocate the side chain from the α-carbon to the backbone nitrogen, conferring intrinsic protease resistance while reproducing the cationic, amphipathic, helix-like architecture of natural AMPs; helical antimicrobial peptoids (“ampetoids”) have achieved low-micromolar, broad-spectrum activity comparable to cationic AMPs such as pexiganan, together with low mammalian-cell toxicity [127]. β-Peptides and other foldamers, including ultrashort cationic β-peptides, similarly recapitulate host-defense-peptide activity in non-natural, protease-stable scaffolds [106]. By decoupling antimicrobial function from L-peptide chemistry, these mimetics circumvent proteolysis entirely, at the cost of more complex synthesis and a comparatively immature regulatory and safety record.

#### 5.2.5. The Activity–Stability–Toxicity Triad and Implications for Delivery

Across these strategies, AMP engineering emerges as a constrained optimization rather than a monotonic improvement: gains in proteolytic stability are repeatedly offset by losses in activity or selectivity, and improvements measured in simplified in vitro assays do not reliably translate in vivo [124]. This realization provides a strong rationale for combining chemical engineering with biomaterial-based delivery (Section 6): the peptide is hardened against degradation at the molecular level, while the carrier localizes it, protects it from the hostile wound microenvironment, and governs its release. Chemical optimization and delivery engineering are therefore best regarded as complementary, rather than alternative, routes toward clinically viable AMP-based wound therapeutics.

### 5.3. Safety, Selectivity, Cytotoxicity and Hemolysis of AMPs

Although AMPs preferentially target negatively charged microbial membranes, host–cell toxicity remains a major translational concern [99,100,101,102,103]. Excessive hydrophobicity, amphipathicity, and membrane activity may compromise selectivity and promote unintended interactions with mammalian cell membranes, resulting in cytotoxicity, hemolysis, or tissue irritation [99,102]. Importantly, the same membrane-disruptive mechanisms responsible for potent antimicrobial activity may also damage keratinocytes, fibroblasts, endothelial cells, and erythrocytes at higher concentrations [17,99].

Balancing antimicrobial efficacy with biocompatibility therefore represents a major challenge in AMP engineering. Increased hydrophobicity often enhances membrane insertion and bacterial killing but may simultaneously increase hemolytic activity and host–cell toxicity [99,102]. Conversely, reducing hydrophobicity may improve safety but compromise antimicrobial potency and biofilm penetration [100,102].

Chronic wounds present additional safety challenges because damaged tissue barriers, persistent inflammation, ischemia, and altered immune responses may increase local susceptibility to cytotoxic effects [46,59]. Furthermore, repeated exposure to high peptide concentrations may potentially disrupt beneficial wound microbiota or alter physiological immune signaling pathways [12]. Consequently, careful optimization of peptide sequence, charge distribution, amphipathicity, and local delivery strategies is essential for maximizing therapeutic selectivity while minimizing host toxicity [99,100,101,102,103].

To move beyond qualitative statements of toxicity, AMP selectivity is best expressed quantitatively through the therapeutic (selectivity) index, conventionally defined as the ratio of the concentration causing 50% hemolysis (HC50), or a defined mammalian-cell cytotoxicity endpoint, to the antimicrobial MIC. A high index denotes a wide therapeutic window in which bacteria are killed at concentrations well below those harmful to host cells, whereas a low index signals that antimicrobial and cytotoxic concentrations overlap dangerously. A recurring and under-reported problem is that many of the most membrane-active peptides effective against biofilms exhibit narrow selectivity indices, exerting measurable cytotoxicity toward the very keratinocytes, fibroblasts, and endothelial cells required for re-epithelialization and granulation [17,99]. This concern is amplified in chronic wounds, where compromised tissue barriers and persistent inflammation may lower the threshold for local irritation and impaired healing [46,59]. Rigorous translational assessment should therefore report selectivity indices against clinically relevant skin cell types rather than antibacterial potency alone, and should recognize that confining the peptide within a delivery system that limits its free concentration at the tissue interface (Section 6) is frequently the most practical route to an acceptable therapeutic window [18,19,99].

### 5.4. Pharmacokinetic and Local Retention Limitations of AMPs

Poor pharmacokinetic performance represents another major limitation of AMP therapy [17,18,19,119]. Free peptides frequently exhibit short biological half-lives because of rapid enzymatic degradation, proteolytic cleavage, renal clearance, and limited tissue retention [17]. In chronic wounds, highly exudative microenvironments may additionally dilute therapeutic concentrations and rapidly remove peptides from the wound surface before sustained antibiofilm activity can be achieved [16,19].

Local retention is particularly important in mature biofilms, where prolonged exposure may be necessary to penetrate EPS barriers, disrupt quorum sensing, and eradicate persister populations [60,61,64,65,66,67,68,69,70,71]. However, many free peptides diffuse rapidly away from target tissues and fail to maintain therapeutic concentrations within deeper biofilm niches [18,19,119]. Moreover, chronic wound hypoxia, ischemia, necrotic tissue, and heterogeneous extracellular matrix architecture may further impair peptide distribution and penetration [46,47,48,49,50,51].

Systemic administration of AMPs also faces significant challenges because of serum instability, off-target interactions, rapid clearance, and potential systemic toxicity [17,99]. As a result, local controlled-release delivery systems are increasingly considered essential for successful AMP-based wound therapy [18,19,119]. Sustained-release biomaterials may improve peptide stability, prolong wound residence time, enhance local concentration, reduce dosing frequency, and minimize systemic exposure [18,19].

### 5.5. Manufacturing, Scalability and Formulation Challenges of AMP-Based Therapeutics

Large-scale AMP production remains expensive and technically demanding compared with many conventional small-molecule antibiotics [99,100]. In concrete terms, the active pharmaceutical ingredient of a conventional small-molecule antibiotic typically costs on the order of cents to a few dollars per gram at manufacturing scale, whereas a peptide active ingredient produced by solid-phase peptide synthesis is generally several-fold to more than an order of magnitude more expensive per gram of finished product; this premium rises steeply with peptide length—each additional residue beyond roughly thirty disproportionately increasing coupling, purification, and synthesis-failure costs—and with stability-enhancing modifications such as D-amino-acid substitution, cyclization, or PEGylation, while fully cGMP-compliant manufacture adds a further substantial increment. Peptide synthesis requires complex manufacturing processes, high-purity reagents, extensive purification steps, and stringent quality-control procedures that substantially increase production costs [17,100,128]. Longer and structurally complex peptides, particularly cyclic peptides or peptides containing multiple disulfide bonds, may present additional manufacturing difficulties and reduced scalability [104,105].

Batch-to-batch reproducibility also represents an important challenge because small variations in peptide folding, purity, aggregation state, or post-translational modification may significantly influence biological activity and toxicity profiles [17]. Furthermore, peptide stability during sterilization, storage, packaging, and incorporation into biomaterials remains insufficiently standardized [18,19]. In practical translational terms, several manufacturing constraints must be addressed explicitly. Peptide active pharmaceutical ingredients must be produced under good manufacturing practice (GMP) conditions at an acceptable cost of goods, which remains substantially higher than for small-molecule antibiotics owing to multi-step solid-phase synthesis, chromatographic purification, and stringent quality control. Equally important is the terminal sterilization of the finished dressing: commonly used methods, including gamma or electron-beam irradiation, ethylene oxide treatment, and steam autoclaving, can each oxidize, cross-link, or denature peptides and degrade the carrier matrix, and therefore must be qualified for the specific peptide–biomaterial combination rather than assumed to be compatible. Finally, defined storage and shelf-life conditions, such as lyophilization, protection from humidity and oxidation, and cold-chain handling, are required to preserve antimicrobial activity from manufacture to point of use. These cost, GMP, sterilization, and stability requirements are frequently underreported in preclinical studies, yet they are decisive for whether a promising AMP dressing can realistically reach the clinic.

Formulation complexity presents another barrier. Successful wound therapeutics must maintain peptide stability while enabling controlled release, biofilm penetration, moisture balance, oxygen permeability, and tissue compatibility simultaneously [18,19,128]. Interactions between peptides and biomaterial matrices may alter peptide conformation, release kinetics, or antimicrobial activity, necessitating extensive optimization and validation [18].

### 5.6. Regulatory and Clinical Trial Barriers for AMP-Based Wound Therapies

Regulatory approval pathways for AMP-based wound therapeutics remain poorly defined and may vary substantially depending on formulation design and intended clinical application [17,18]. AMP-loaded wound dressings may potentially be classified as drugs, medical devices, biologics, or combination products, each requiring distinct regulatory pathways and clinical evidence standards. This complexity creates uncertainty during translational development and may substantially delay commercialization.

A concrete example clarifies why this classification problem is consequential. Consider an alginate or hydrocolloid dressing loaded with an antimicrobial peptide: although marketed as a single product, it combines a device-like matrix, whose primary action is to maintain a moist healing environment, with a pharmacologically active peptide whose primary action is antimicrobial. Whether the product is regulated as a drug, a device, or a drug–device combination depends on which mode of action is deemed primary, and this determination diverges across jurisdictions. In the United States, the FDA assigns a combination product to a lead center based on its primary mode of action, so an AMP-led dressing would typically follow a drug-led pathway requiring full pharmaceutical evidence, whereas a matrix-led dressing with ancillary antimicrobial action might follow a device-led pathway. In the European Union, the same product would generally be evaluated under the Medical Device Regulation as a device incorporating an ancillary medicinal substance, triggering consultation with a medicines authority while retaining a device certification route. These divergent classifications impose substantially different requirements for clinical evidence, manufacturing controls, and post-market surveillance, so a development program optimized for one pathway may be poorly positioned for another. Early and explicit determination of the intended regulatory route is therefore as decisive to translational success as the underlying antimicrobial performance [17,18].

Clinical trial design in chronic wound research presents additional challenges [91,92,93,94]. Chronic wounds are highly heterogeneous with respect to etiology, vascular status, microbial composition, patient comorbidities, ischemia severity, glycemic control, and biofilm burden [4,5,46]. Consequently, standardization of inclusion criteria, wound classification systems, clinical endpoints, and microbiological assessments remains difficult [91,92,93,94].

Moreover, clinically meaningful endpoints extend beyond simple bacterial reduction and may include wound closure rates, recurrence prevention, pain reduction, quality of life, limb salvage, and reduction in systemic antibiotic use [91,92]. Large phase III trials evaluating AMP-based wound therapies remain scarce, and long-term safety data are still limited [17,119]. These regulatory and clinical uncertainties continue to slow translational progress despite promising preclinical findings.

### 5.7. Preclinical Model and Standardization Gaps in AMP Research

A major but frequently underrecognized limitation in AMP research is the poor translational relevance of many preclinical models [17,18,19]. Numerous studies evaluate antimicrobial activity using planktonic bacteria, mono-species biofilms, short incubation periods, and simplified in vitro conditions that fail to reproduce the complexity of human chronic wounds [60,61,64,65,66,67,68,69,70,71,129]. However, clinical chronic wounds typically contain mature polymicrobial biofilms embedded within protease-rich, hypoxic, ischemic, and inflammatory microenvironments [46,47,48,49,50,51,52,53,54,55,56,57,58,59,120,121,130].

Importantly, antimicrobial efficacy against planktonic bacteria does not necessarily predict activity against mature biofilms [60,61,64,65,66,67,68,69,70,71]. Biofilm-associated microorganisms exhibit profound metabolic heterogeneity, reduced growth rates, altered gene expression, quorum-sensing activation, and increased antimicrobial tolerance compared with planktonic counterparts [60,61,64,65,66,67,68,69,70,71]. Therefore, reliance on minimum inhibitory concentration (MIC) testing alone may substantially overestimate clinical therapeutic potential [129].

Animal models also present important translational limitations. Murine acute wound models are widely used because of their low cost and experimental convenience; however, rodent skin differs substantially from human skin in thickness, immune composition, contraction-based healing, hair density, and microbiome composition [35]. Furthermore, many animal studies fail to incorporate clinically relevant comorbidities such as diabetes, peripheral vascular disease, ischemia, aging, or recurrent polymicrobial infection [46,58,130].

Another major challenge is the lack of standardized biofilm quantification methodologies and reproducible wound models [91,92,93,94,129]. Studies frequently use different bacterial strains, incubation times, peptide concentrations, endpoints, and analytical techniques, making cross-study comparison difficult. Variability in biofilm maturity, matrix composition, and experimental conditions further complicates reproducibility and translational interpretation [129].

Importantly, relatively few studies evaluate AMP efficacy in mature polymicrobial biofilms derived from clinically relevant chronic wound isolates [64,72,73,74,75,120,121]. Likewise, large-animal chronic wound models capable of better replicating human wound physiology remain underutilized because of cost and logistical complexity [130]. These limitations collectively contribute to the persistent gap between promising laboratory findings and successful clinical translation.

### 5.8. Biomaterial-Enabled Strategies to Overcome AMP Translational Barriers

Collectively, these translational barriers demonstrate that biomaterials should not be considered passive AMP carriers but rather enabling therapeutic platforms [18,19,128]. Advanced biomaterial systems may protect peptides from proteolytic degradation, prolong local retention, improve controlled release, enhance biofilm penetration, reduce cytotoxicity, and support tissue regeneration simultaneously [18,19,119,128].

Consequently, integration of AMPs into hydrogels, electrospun nanofibers, nanoparticles, scaffolds, films, and multifunctional smart dressings has emerged as one of the most promising strategies for overcoming current translational limitations [18,19]. Such platforms may enable localized and sustained AMP delivery while simultaneously modulating chronic wound microenvironments, disrupting biofilms, and promoting regenerative healing processes [18,19,128]. These biomaterial-enabled approaches therefore represent a critical next step toward clinically effective AMP-based chronic wound therapeutics.

**Figure 5:**
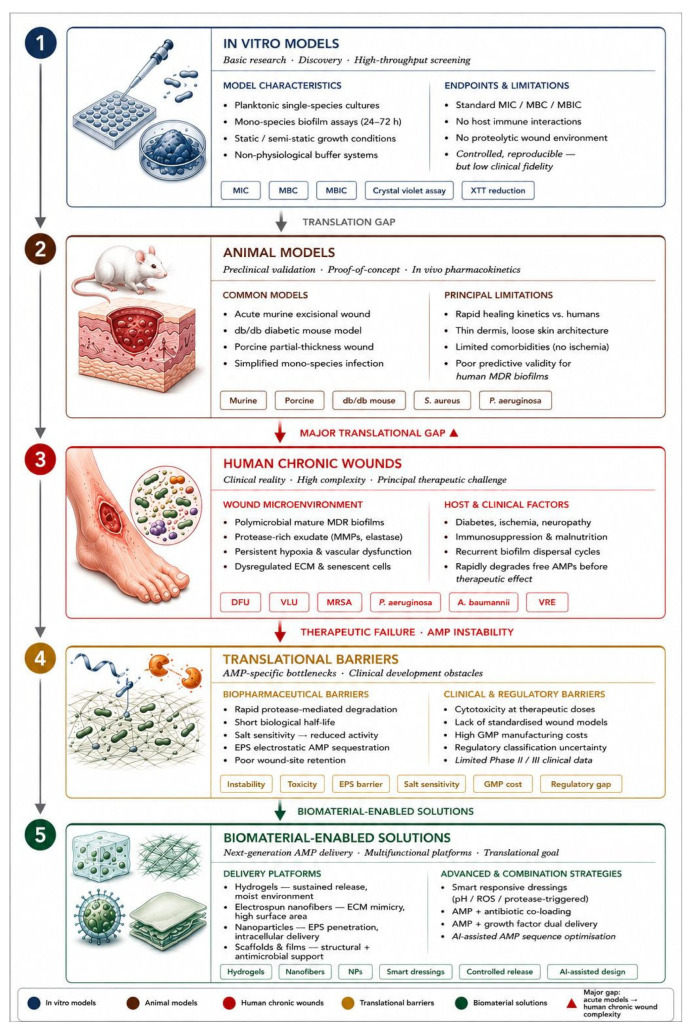
Bench-to-bedside translational gaps in AMP-based wound therapy. Abbreviations: AMP, antimicrobial peptide; DFU, diabetic foot ulcer; ECM, extracellular matrix; EPS, extracellular polymeric substance; GMP, good manufacturing practice; MBIC, minimum biofilm inhibitory concentration; MBC, minimum bactericidal concentration; MDR, multidrug-resistant; MIC, minimum inhibitory concentration; MMP, matrix metalloproteinase; MRSA, methicillin-resistant *Staphylococcus aureus*; NP, nanoparticle; ROS, reactive oxygen species; VLU, venous leg ulcer; VRE, vancomycin-resistant enterococci.

Figure 5 presents the five-stage translational pipeline for the development of AMP-based therapeutics from early in vitro discovery to advanced biomaterial-enabled clinical strategies for chronic wound management.

- Stage 1 covers conventional in vitro models for the AMP discovery and screening process, including planktonic bacterial cultures and mono-species biofilm assays, under highly controlled and reproducible laboratory conditions. While these systems enable standardized measurement of antimicrobial activity using minimum inhibitory concentration (MIC), minimum bactericidal concentration (MBC) and minimum biofilm inhibitory concentration (MBIC) assays, they do not mimic essential features of the chronic wound microenvironment, such as immune interactions, polymicrobial complexity, hypoxia and protease-rich exudates.
- Stage 2 describes frequently used preclinical animal models, including acute murine excisional wounds, diabetic db/db mice, and porcine wound models. While these systems provide proof-of-concept validation and preliminary pharmacokinetic data, they incompletely reproduce the pathophysiological complexity of human chronic wounds due to rapid healing kinetics, structural differences in skin architecture, simplified microbial infections, and limited representation of chronic comorbidities such as ischemia and neuropathy. The transition from animal studies to clinical chronic wound settings therefore represents a major translational gap.
- Stage 3 represents the clinical reality of human chronic wounds, characterized by polymicrobial mature multidrug-resistant (MDR) biofilms, persistent hypoxia, vascular dysfunction, dysregulated extracellular matrix (ECM) remodeling, excessive protease activity, senescent cell accumulation, and recurrent biofilm dispersal cycles. Host-related factors, including diabetes mellitus, ischemia, immunosuppression, malnutrition, and neuropathy, further contribute to impaired healing responses and rapid degradation of free AMPs before sustained therapeutic activity can be achieved.
- Stage 4 describes major translational challenges that are blocking the clinical application of AMP-based therapies. These challenges include rapid protease-mediated degradation, short biological half-life, salt sensitivity, EPS-mediated sequestration, poor wound-site retention and cytotoxicity at therapeutic concentrations. Additional barriers include the lack of standardized chronic wound models, high costs of good manufacturing practice (GMP)-compliant production, regulatory uncertainty and limited availability of large-scale Phase II and III clinical studies.
- Stage 5 addresses these translational limitations with next-generation biomaterial-enabled solutions, including hydrogels, electrospun nanofibers, nanoparticles, scaffolds, and smart responsive wound dressings that can protect AMPs from degradation, enable sustained local release, improve EPS penetration, and simultaneously support tissue regeneration. Advanced strategies include AMP–antibiotic co-delivery systems, dual AMP–growth factor platforms, and artificial intelligence-assisted AMP sequence optimization for improved antimicrobial potency, stability, and translational performance.

## 6. Biomaterial-Enabled AMP Delivery Systems for Chronic Biofilm-Infected Wounds

Advanced biomaterial platforms are increasingly recognized as essential enabling technologies for successful antimicrobial peptide (AMP)-based wound therapy rather than passive drug carriers alone [17,18,19]. Chronic wounds represent highly hostile pathological environments characterized by excessive protease activity, oxidative stress, hypoxia, abnormal pH, polymicrobial biofilms, ischemia, and persistent inflammation, all of which substantially impair the stability and efficacy of free AMPs [16,46,47,48,49,50,51,52,53,54,55,56,57,58,59]. Consequently, modern biomaterial systems are increasingly engineered not only to protect peptides from degradation, but also to improve local retention, enable controlled release, enhance biofilm penetration, modulate wound microenvironments, and simultaneously promote tissue regeneration [17,18,19,131,132,133,134,135].

Importantly, biomaterial-assisted AMP delivery directly addresses several translational barriers discussed in Section 5, including biochemical instability, rapid peptide clearance, cytotoxicity, insufficient wound-site retention, and poor reproducibility under clinically relevant wound conditions [17,18,19]. Current research increasingly focuses on multifunctional systems capable of combining antimicrobial, antibiofilm, immunomodulatory, angiogenic, antioxidative, and regenerative properties within a single therapeutic platform [131,132,133,134,135].

A decisive but frequently underspecified determinant of therapeutic success is the release kinetics of the peptide from its carrier. The rate at which an AMP is liberated is governed jointly by the physical architecture of the matrix, principally the polymer mesh (pore) size relative to the hydrodynamic radius of the peptide, and by the chemical affinity between peptide and carrier, including electrostatic, hydrophobic, and reversible covalent interactions. When the mesh size greatly exceeds peptide dimensions and affinity is low, release approximates rapid Fickian diffusion and tends toward an initial burst; tighter networks, higher charge complementarity, or cleavable tethers instead yield sustained, near-zero-order release [17,18,19,131,132,133]. From a therapeutic standpoint, the objective is to maintain the local peptide concentration within a defined window: high enough to exceed the MBEC against biofilm-embedded organisms, yet below the threshold for host–cell cytotoxicity. An uncontrolled burst risks transient cytotoxic peaks and premature depletion, whereas an overly retentive system may deliver persistently sub-inhibitory concentrations that fail to eradicate the biofilm and can impose selective pressure favoring tolerant or resistant subpopulations [10,12,17]. Rational matching of mesh size and binding affinity to the physicochemical profile of the peptide is therefore central to converting in vitro potency into durable in vivo efficacy.

### 6.1. AMP-Loaded Hydrogels as Biofilm-Modulating Wound Matrices

Hydrogels are among the most extensively investigated AMP delivery platforms because of their elevated water content, tissue-like mechanical properties, moisture-retention capacity, and ability to provide sustained local peptide release [18,19]. In chronic wound therapy, hydrogels additionally function as bioactive wound matrices capable of maintaining moist healing environments, absorbing exudate, supporting cell migration, and modulating inflammatory responses [18,107,131]. A consolidated comparison of the biomaterial platforms is discussed in this section and in Section 6.5, including their delivery advantages, biofilm-related functions, regenerative roles, and principal limitations; the text below therefore emphasizes mechanism and synthesis rather than re-listing each carrier’s properties.

In our assessment, the most informative hydrogel data come from systems that pair a defined peptide with an infected-diabetic in vivo model and report a quantitative bacterial-load reduction; reports limited to in vitro inhibition-zone data should be weighted accordingly.

#### 6.1.1. Natural Hydrogel Systems

Natural hydrogels, including chitosan, alginate, collagen, gelatin, hyaluronic acid, fibrin, and cellulose-derived systems, demonstrate high biocompatibility and structural similarity to extracellular matrix (ECM) components [18,108]. Chitosan-based hydrogels are particularly attractive because chitosan itself possesses intrinsic antimicrobial, hemostatic, mucoadhesive, and wound-healing properties [109]. Moreover, the cationic nature of chitosan may synergize with AMP activity by enhancing bacterial membrane interaction and destabilizing negatively charged biofilm matrices [109,110].

Alginate hydrogels are widely investigated for exudative chronic wounds because of their excellent fluid absorption capacity and ability to form soft gel matrices in the presence of calcium ions [111]. Collagen- and gelatin-based systems provide biomimetic environments that support fibroblast proliferation, angiogenesis, and keratinocyte migration while simultaneously enabling controlled peptide delivery [113]. Hyaluronic acid hydrogels additionally contribute to immune modulation and tissue remodeling because of their involvement in physiological wound healing pathways [114].

Importantly, natural hydrogels may protect AMPs from proteolytic degradation and prolong local retention within protease-rich chronic wound environments [18,19].

#### 6.1.2. Synthetic Hydrogel Systems

Synthetic hydrogels based on polyethylene glycol (PEG), polyvinyl alcohol (PVA), polyacrylamide, polyurethane, and related polymers offer superior tunability of mechanical properties, degradation rates, porosity, and release kinetics compared with many natural biomaterials [116]. These systems may be engineered to optimize peptide loading, diffusion, swelling behavior, and wound-site residence time while minimizing premature peptide release [18].

PEG-based hydrogels are particularly attractive because of their hydrophilicity, biocompatibility, and ability to reduce nonspecific protein adsorption [116]. PVA hydrogels demonstrate excellent flexibility and mechanical stability suitable for chronic wound dressings, while polyurethane systems may provide improved elasticity and oxygen permeability [117]. Synthetic matrices additionally facilitate incorporation of multifunctional components, including nanoparticles, oxygen-generating systems, ROS scavengers, and growth factors [118].

Recent studies have also demonstrated that synthetic antibiofilm hydrogels may combine AMP release with antioxidative and anti-inflammatory functions to improve healing outcomes in diabetic and infected wounds [132,133].

#### 6.1.3. Self-Assembling AMP Hydrogels

Self-assembling peptide hydrogels represent one of the most innovative strategies in AMP delivery because the peptide itself may simultaneously function as both structural biomaterial and therapeutic antimicrobial agent [119,130]. These systems spontaneously organize into nanofibrous three-dimensional architectures through noncovalent interactions, including hydrogen bonding, electrostatic attraction, π–π stacking, and hydrophobic interactions [119].

Self-assembling AMP hydrogels may exhibit intrinsic antibiofilm activity, injectability, shear-thinning behavior, and biomimetic ECM-like organization [130]. Importantly, nanoscale fibrous networks may enhance cell infiltration, angiogenesis, and tissue regeneration while enabling prolonged peptide retention within infected wound environments [119,130].

Recent supramolecular peptide nanocomposite hydrogels have demonstrated potent activity against polymicrobial biofilms while simultaneously accelerating diabetic wound healing and reducing inflammatory signaling [135]. Such multifunctional systems are increasingly viewed as next-generation wound therapeutics because they integrate structural support with antimicrobial and regenerative functionality.

#### 6.1.4. Stimuli-Responsive AMP Hydrogels

Stimuli-responsive hydrogels are increasingly engineered to achieve infection-triggered or wound-responsive AMP release [133,134]. Chronic wounds exhibit distinctive pathological characteristics, including acidic pH, elevated reactive oxygen species (ROS), excessive protease activity, bacterial enzymes, and inflammatory mediators, all of which may serve as biological triggers for controlled therapeutic activation [46,47,48,49,50,51,52,53,54,55,56,57,58,59].

pH-responsive systems may selectively release AMPs within acidic infected wound environments, thereby minimizing premature peptide loss and improving therapeutic specificity [133,134]. ROS-responsive hydrogels are particularly attractive for chronic wound applications because excessive oxidative stress represents a hallmark of persistent biofilm-associated inflammation [52,53]. These systems may simultaneously scavenge ROS and release antimicrobial agents in response to oxidative microenvironments [132,133].

Enzyme-responsive hydrogels capable of responding to bacterial proteases or matrix metalloproteinases (MMPs) additionally provide opportunities for targeted peptide delivery within infected tissues [134,136]. Such systems may improve peptide stability, reduce systemic exposure, and optimize localized therapeutic activity within chronic wound microenvironments.

#### 6.1.5. Injectable and Sprayable AMP Hydrogels

Injectable and sprayable hydrogels are gaining increasing clinical interest because they enable minimally invasive application to irregular wound geometries, burn wounds, deep tissue defects, and tunneling ulcers [129]. Injectable systems may conform closely to wound architecture while maintaining high local AMP concentrations and sustained release profiles [129,131].

Sprayable hydrogels additionally facilitate homogeneous wound coverage and may reduce tissue trauma during dressing changes [131]. Several recent systems combine injectable hydrogels with nanoparticles, oxygen-generating agents, stem-cell-derived exosomes, or photothermal materials to achieve multifunctional antimicrobial and regenerative effects [132,133,134].

Importantly, these adaptable systems are particularly promising for chronic wound management because they may improve peptide distribution throughout heterogeneous biofilm-containing wound beds while simultaneously supporting tissue repair and exudate management. In our assessment, the most informative hydrogel data come from systems that pair a defined peptide with an infected-diabetic in vivo model and report a quantitative bacterial-load reduction; reports limited to in vitro inhibition-zone data should be weighted accordingly.

### 6.2. Electrospun Nanofibers for Sustained AMP Delivery and ECM Mimicry

Electrospun nanofibers have emerged as highly promising AMP delivery platforms because of their structural similarity to native extracellular matrix architecture, high surface-area-to-volume ratio, tunable porosity, and ability to provide sustained therapeutic release [137,138,139,140]. Nanofibrous wound dressings may additionally support oxygen diffusion, exudate management, fibroblast migration, and keratinocyte proliferation while physically protecting wounds from external contamination [137,139].

Electrospinning additionally enables incorporation of AMPs into core–shell fibers, multilayer nanofibers, coaxial structures, and hybrid nanocomposites designed to optimize peptide stability and release kinetics [137,138]. AMP-loaded nanofibers may provide prolonged antimicrobial activity against mature biofilms while reducing systemic toxicity and maintaining localized therapeutic concentrations [137,138,139,140]. As a concrete example, Su et al. encapsulated the molecularly engineered cathelicidin derivative 17BIPHE2 in the core of pluronic F127/PCL core–shell nanofibers, achieving sustained release over four weeks; in a type II diabetic biofilm chronic-wound model these dressings reduced the burden of MRSA USA300 by roughly five orders of magnitude, and eliminated the biofilm completely when combined with debridement [141].

Recent studies have demonstrated that AMP-functionalized electrospun dressings exhibit potent activity against multidrug-resistant pathogens, including methicillin-resistant *Staphylococcus aureus* (MRSA), *Pseudomonas aeruginosa*, and colistin-resistant Gram-negative bacteria [140]. Importantly, electrospun systems may additionally serve as multifunctional regenerative scaffolds capable of simultaneously delivering antimicrobial peptides, growth factors, nanoparticles, antioxidants, or oxygen-generating compounds [137,138,139].

Across electrospun systems, sustained release and ECM mimicry are well established, but quantitative endpoints against mature biofilms remain the exception rather than the rule.

### 6.3. AMP-Functionalized Films, Membranes and Surface Coatings

Films, membranes, and AMP-functionalized coatings represent another important strategy for preventing microbial adhesion and biofilm formation at wound interfaces [136,142,143]. Surface-immobilized AMPs may generate contact-killing surfaces that disrupt bacterial membranes upon direct contact while minimizing uncontrolled peptide diffusion [136].

AMP-functionalized coatings are increasingly investigated for wound dressings, burn surfaces, catheters, implants, and medical-device interfaces because they may reduce microbial colonization and prevent biofilm establishment during early infection stages [136,143]. Enzyme-responsive coating systems capable of infection-triggered AMP release have additionally demonstrated promising antibiofilm activity against resistant wound pathogens [136].

Recent smart wound dressings integrating AMP-functionalized films with fluorescence imaging, aggregation-induced emission (AIE) systems, or biosensing capabilities may additionally enable simultaneous infection monitoring and antimicrobial therapy [143]. Such multifunctional systems represent an important emerging direction in precision wound therapeutics.

### 6.4. Porous Scaffolds, Cryogels and Sponges for Regenerative AMP Delivery

Porous scaffolds, cryogels, and sponge-like biomaterials provide three-dimensional regenerative microenvironments capable of supporting cell infiltration, vascularization, nutrient diffusion, and tissue integration [18,19]. Their highly porous architecture additionally enables efficient exudate absorption and oxygen exchange while facilitating sustained AMP release [18].

Cryogels are particularly attractive because of their interconnected macroporous structure, mechanical resilience, injectability, and ability to accommodate high therapeutic loading capacities [18]. Sponge-like biomaterials may additionally support granulation tissue formation and angiogenesis while simultaneously disrupting mature biofilms [18,19].

Several recent scaffold systems combine AMPs with nanomaterials, stem-cell-derived products, oxygen-generating compounds, or immunomodulatory agents to create multifunctional regenerative wound platforms [135,144]. These integrated systems are increasingly viewed as promising approaches for severe chronic wounds characterized by tissue loss, ischemia, and recurrent infection.

### 6.5. Nanotechnology-Enabled AMP Delivery Systems

Nanotechnology-based delivery systems represent one of the most rapidly evolving fields in AMP therapeutics because nanoscale carriers may substantially improve peptide stability, bioavailability, biofilm penetration, and controlled release [144,145,146,147]. Importantly, nanocarriers may additionally reduce peptide toxicity and enhance retention within infected wound environments [144]. Metallic–nanoparticle/AMP hybrids consistently report the largest log-reductions, yet their translational value depends on resolving the metal-accumulation and cytotoxicity concerns that the wound-healing data rarely address directly.

#### 6.5.1. Liposomes and Lipid Nanocarriers

Liposomes and lipid-based nanocarriers may encapsulate AMPs within phospholipid bilayers that protect peptides from enzymatic degradation while facilitating membrane fusion and biofilm penetration [144,145]. Such systems may additionally reduce cytotoxicity and improve therapeutic selectivity toward bacterial membranes [144].

#### 6.5.2. Polymeric Nanoparticles

Polymeric nanoparticles based on PLGA, chitosan, alginate, and related materials may provide tunable peptide release profiles, enhanced wound retention, and improved antibiofilm activity [144,145]. Chitosan nanoparticles are particularly attractive because of their intrinsic antimicrobial and mucoadhesive properties that may synergize with AMP-mediated bacterial killing [109,144].

#### 6.5.3. Metallic Nanoparticles

Metallic nanoparticles, including silver, gold, zinc oxide, and copper nanoparticles, are increasingly combined with AMPs to achieve synergistic antimicrobial activity [146,147]. Such systems may enhance membrane disruption, ROS generation, and biofilm destabilization while reducing the likelihood of resistance development [147].

#### 6.5.4. Nanoemulsions, Micelles and Dendrimers

Nanoemulsions and micellar systems may improve peptide solubility, penetration, and local delivery within mature biofilms [144]. Dendrimer-based systems additionally provide high loading capacity and multivalent peptide presentation capable of enhancing microbial membrane interaction and antibiofilm activity [145].

Importantly, several recent studies have demonstrated that nanotechnology-enabled AMP systems may synergize with antibiotics, antiseptics, photodynamic therapy, or anti-quorum-sensing compounds to improve eradication of resistant polymicrobial biofilms [145,146]. Notably, metallic–nanoparticle/AMP hybrids consistently report the largest log-reductions, yet their translational value depends on resolving the metal-accumulation and cytotoxicity concerns that the wound-healing data rarely address directly.

### 6.6. Smart and Responsive AMP Biomaterials

Smart biomaterials capable of dynamically responding to wound microenvironments represent one of the most promising directions in chronic wound therapeutics [133,134,136,142]. This subsection introduces them only briefly to complete the platform overview begun in this section; their stimulus-specific designs, representative systems, and translational evidence are treated in detail in Section 7, and are summarized here rather than restated to avoid redundancy. These systems may respond to pH changes, ROS accumulation, bacterial enzymes, glucose concentrations, inflammatory mediators, temperature, or external stimuli to achieve controlled therapeutic activation [133].

In diabetic wound therapy, glucose-responsive systems may adapt AMP release according to local metabolic conditions, while ROS-responsive systems may simultaneously reduce oxidative stress and bacterial burden [132,133]. Infection-triggered systems capable of responding to bacterial proteases or quorum-sensing molecules additionally offer opportunities for highly selective antimicrobial therapy [134,136].

Several recent smart platforms integrate AMP delivery with photothermal therapy, photodynamic therapy, electrical stimulation, biosensing technologies, or real-time infection monitoring [142,143]. Such multifunctional biomaterials may ultimately support personalized wound management strategies capable of simultaneously addressing infection, inflammation, oxidative stress, and impaired tissue regeneration.

### 6.7. Translational Perspectives and Design Principles for Next-Generation AMP Biomaterials

Collectively, current evidence suggests that biomaterials should not be viewed merely as passive AMP carriers but rather as multifunctional therapeutic platforms capable of actively modulating chronic wound microenvironments [17,18,19]. Advanced biomaterial systems may simultaneously improve peptide stability, prolong local retention, enhance biofilm penetration, reduce toxicity, regulate inflammation, and promote tissue regeneration [18,19,144,145,146].

Future translational development will likely focus on multifunctional and personalized systems integrating AMP therapy with responsive biomaterials, antibiofilm technologies, regenerative medicine approaches, and precision wound diagnostics [133,134,135,136,137,138,139,140,142,143,144,145,146,147]. Importantly, successful clinical translation will require improved standardization of biofilm models, clinically relevant chronic wound systems, scalable manufacturing strategies, and robust clinical validation [17,18,19].

Consequently, biomaterial-enabled AMP delivery systems represent one of the most promising strategies for overcoming current limitations in chronic biofilm-infected wound therapy and may ultimately facilitate development of next-generation integrated wound therapeutics capable of simultaneously targeting infection, biofilm persistence, inflammation, and tissue regeneration. The principal biomaterial platform classes and their AMP-delivery characteristics are summarized in Table 2.

**Figure 6:**
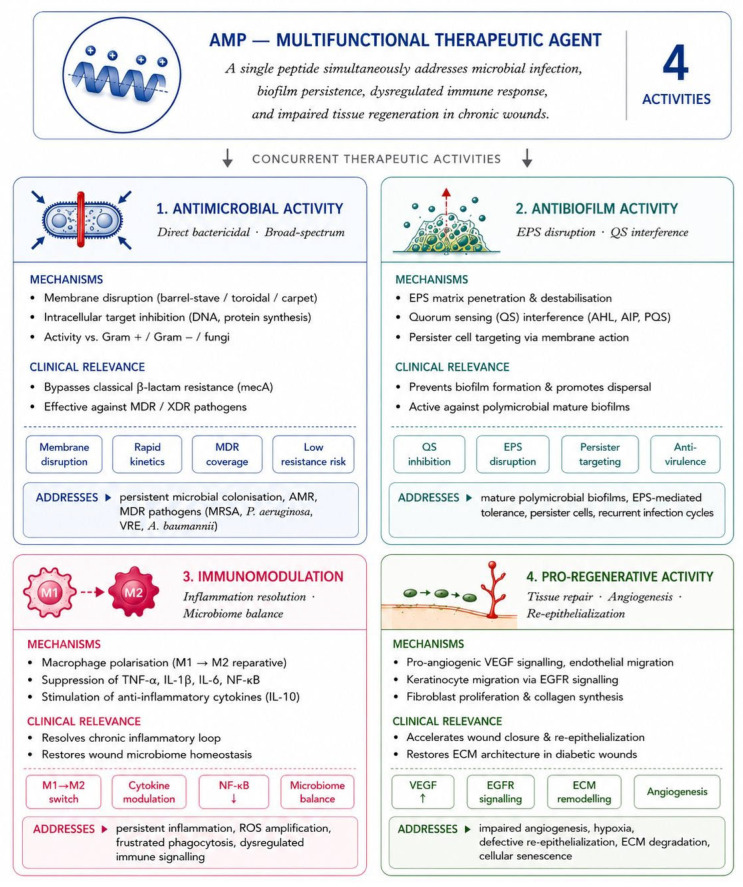
The intrinsic multifunctional repertoire of free antimicrobial peptides: four concurrent therapeutic activities addressing the core pathologies of chronic wounds. Abbreviations: AHL, acyl-homoserine lactone; AIP, autoinducing peptide; AMP, antimicrobial peptide; ECM, extracellular matrix; EGFR, epidermal growth factor receptor; EPS, extracellular polymeric substance; IL, interleukin; MDR, multidrug resistant; MRSA, methicillin resistant *Staphylococcus aureus*; NF-κB, nuclear factor kappa B; PQS, Pseudomonas quinolone signal; QS, quorum sensing; ROS, reactive oxygen species; TNF-α, tumor necrosis factor-α; VEGF, vascular endothelial growth factor; VRE, vancomycin-resistant enterococci; XDR, extensively drug resistant.

The upper central hub of Figure 6 highlights the concept of AMPs as multifunctional therapeutic agents capable of simultaneously targeting microbial infection, biofilm persistence, dysregulated inflammation and impaired tissue regeneration within a single molecular platform. Individual AMPs may exert complementary therapeutic activities that directly address several of the major pathological drivers responsible for chronic wound persistence.

The antimicrobial activity panel summarizes the direct bactericidal effects of AMPs through membrane disruption and intracellular target inhibition (detailed in Section 4.2), supporting broad-spectrum activity against Gram-positive, Gram-negative, and fungal pathogens, including MDR and XDR organisms, with a low propensity for classical resistance. The antibiofilm activity panel demonstrates AMP-induced destruction of mature polymicrobial biofilms by destabilization of EPS, interference of quorum sensing (QS) and targeting of metabolically dormant persister cells. By infiltrating and destabilizing the biofilm matrix, AMPs may reduce microbial tolerance and help prevent recurrent infection cycles characteristic of chronic wounds.

The immunomodulatory activity panel suggests that AMPs can modulate the host immune response by macrophage repolarization from pro-inflammatory M1 phenotypes to reparative M2 phenotypes, inhibition of pro-inflammatory cytokines and NF-κB signaling pathways, and stimulation of anti-inflammatory mediators such as interleukin-10 (IL-10), resulting in the resolution of the chronic inflammatory loop and restoring wound immune homeostasis.

The pro-regenerative activity panel depicts the regenerative properties of AMPs, including induction of angiogenesis by vascular endothelial growth factor (VEGF), epidermal growth factor receptor (EGFR)-dependent keratinocyte migration, fibroblast proliferation, collagen synthesis, extracellular matrix (ECM) remodeling, and acceleration of re-epithelialization. Collectively, these effects contribute to restoration of tissue architecture and wound closure.

Unlike traditional antibiotics, which typically act on isolated microbial pathways and lack tissue reparative or immunoregulatory activities, AMPs may integrate antimicrobial, antibiofilm, immunomodulatory and regenerative functions within a single peptide-based therapeutic platform.

**Figure 7:**
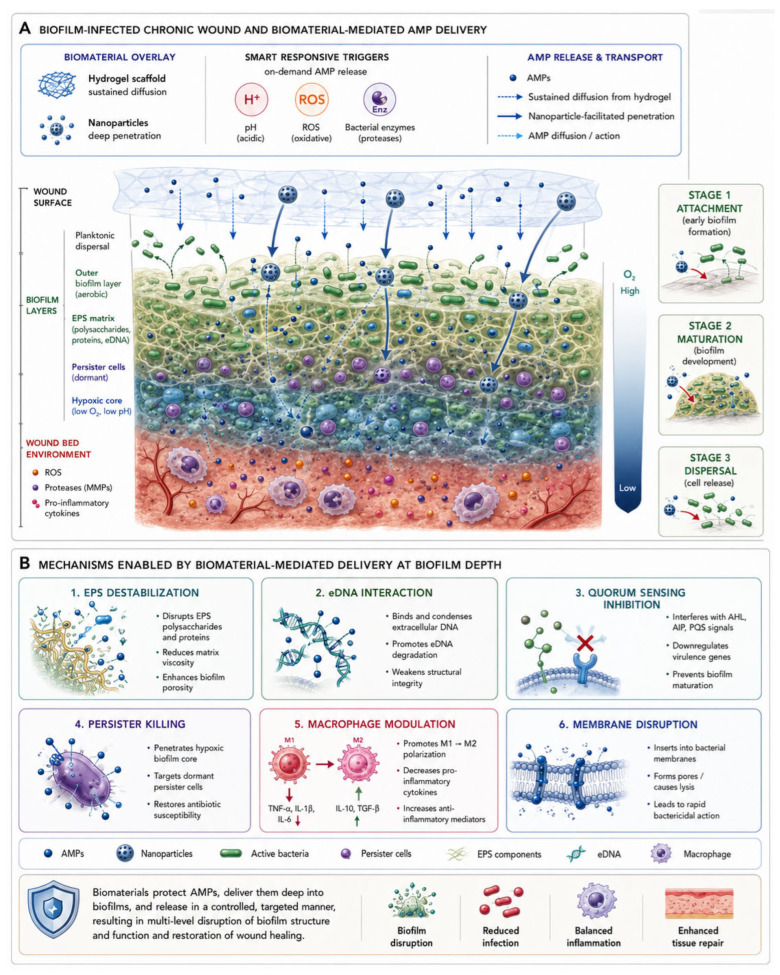
Biomaterial-mediated delivery and carrier-enabled potentiation of AMP activity within the chronic wound biofilm. (**A**) Biofilm-infected chronic wound and biomaterial-mediated AMP delivery. A cross-section of a biofilm-infected wound showing biomaterial delivery systems—hydrogel scaffolds for sustained diffusion and nanoparticles for deep penetration—that release antimicrobial peptides on demand via smart triggers (acidic pH, ROS, bacterial enzymes). AMPs travel through the stratified biofilm layers (aerobic outer layer, EPS matrix, dormant persister cells, hypoxic core) into the wound bed, paralleling the biofilm stages of attachment, maturation, and dispersal. (**B**) Mechanisms enabled by biomaterial-mediated delivery at biofilm depth. Six therapeutic mechanisms achieved once AMPs reach the biofilm: EPS destabilization, eDNA binding and degradation, quorum sensing inhibition, persister cell killing, macrophage modulation (M1→M2 polarization), and direct membrane disruption—together yielding biofilm disruption, reduced infection, balanced inflammation, and enhanced tissue repair. Abbreviations: AHL, acyl-homoserine lactone; AIP, autoinducing peptide; AMP, antimicrobial peptide; eDNA, extracellular DNA; EPS, extracellular polymeric substance; IL, interleukin; MMP, matrix metalloproteinase; PQS, Pseudomonas quinolone signal; QS, quorum sensing; ROS, reactive oxygen species; TGF-β, transforming growth factor-β; TNF-α, tumor necrosis factor-α.

Figure 7A illustrates a mechanistic cross-sectional representation of a chronic wound colonized by a mature polymicrobial biofilm and overlaid with AMP-loaded biomaterial systems. The upper wound surface contains a hydrogel-based biomaterial scaffold that enables sustained AMP diffusion and controlled local release. Nanoparticle-mediated carriers ensure deep penetration of antimicrobial peptides within the biofilm architecture.

Smart responsive biomaterials are activated by common chronic wound microenvironmental signals, including acidic pH, reactive oxygen species (ROS) and bacterial proteases, to enable on-demand AMP release.

The mature biofilm is depicted as a spatially heterogeneous structure composed of an aerobic outer layer; a dense EPS matrix containing polysaccharides, proteins, and extracellular DNA (eDNA); metabolically dormant persister cells; and a hypoxic acidic core associated with low oxygen tension and impaired antimicrobial susceptibility. Planktonic bacterial dispersal from the superficial biofilm layer contributes to recurrent wound colonization. The local wound bed microenvironment is characterized by chronic inflammation, increased ROS generation, matrix metalloproteinases (MMPs), pro-inflammatory cytokines, and tissue destruction. The diffusion pathways of AMP and nanoparticle-facilitated transport pathways show increased penetration through the EPS barrier and into deep biofilm compartments.

Figure 7B shows the major molecular mechanisms by which biomaterial-enabled AMP delivery can disrupt chronic wound biofilms. EPS destabilization increases matrix porosity and reduces biofilm structural integrity by disrupting extracellular polysaccharides and proteins. eDNA interaction promotes the condensation and degradation of extracellular DNA, further destabilizing matrix cohesion. Quorum sensing inhibition disrupts AHL-, AIP- and PQS-mediated bacterial communication pathways, thus reducing virulence signaling and biofilm maturation. Persister cell killing allows for AMP penetration into hypoxic biofilm regions and targets dormant bacterial subpopulations responsible for antibiotic tolerance and recurrent infection. Macrophage modulation leads to repolarization of pro-inflammatory M1 phenotypes to reparative M2 phenotypes, reducing TNF-α, IL-1β and IL-6 signaling and increasing anti-inflammatory mediators such as IL-10 and TGF-β. Membrane disruption represents the direct bactericidal activity of AMPs, whereby peptide insertion into bacterial membranes leads to pore formation and rapid membrane destabilization.

Collectively, biomaterials protect AMPs from premature degradation, enhance deep biofilm penetration, enable controlled and stimuli-responsive release, and help restore antimicrobial, immunological, and regenerative functions within the chronic wound microenvironment.

## 7. Smart and Responsive Biomaterial Systems for AMP-Based Chronic Wound Therapy

Smart AMP-enabled biomaterial systems have emerged as one of the most promising translational strategies for overcoming the multifactorial barriers limiting antimicrobial peptide (AMP)-based chronic wound therapy [17,18,19]. Unlike passive wound dressings, next-generation biomaterials are increasingly engineered as pathology-responsive therapeutic microenvironments capable of dynamically interacting with the biochemical and microbiological features of chronic wounds, including alkaline pH, excessive reactive oxygen species (ROS), protease-rich exudate, hypoxia, polymicrobial biofilms, and persistent inflammation [46,47,48,49,50,51,52,53,54,55,56,57,58,59]. These systems aim not only to deliver AMPs locally, but also to regulate peptide release kinetics, improve biofilm penetration, shield peptides from enzymatic degradation, reduce cytotoxicity, and simultaneously support tissue regeneration [17,18,19,148,149,150,151].

The systems described in Section 6 and those described here therefore occupy two complementary delivery regimes. Section 6 covers predominantly passive, constitutive platforms—hydrogels, electrospun nanofibers, films, and nanoparticles—that protect the peptide and release it in a sustained, environment-independent manner, whereas the present section covers active, pathology-responsive platforms that withhold the peptide until a wound-specific trigger (acidic pH, ROS, or protease activity) is encountered and then release it on demand. This passive-versus-active distinction, rather than the carrier material itself, is the organizing principle separating the two sections.

Importantly, the rationale for responsive biomaterials derives directly from chronic wound biology. Mature chronic wounds exhibit elevated pH, excessive oxidative stress, abundant matrix metalloproteinases (MMPs), bacterial proteases, ischemia, and dysregulated inflammatory signaling, all of which impair conventional wound therapies and accelerate AMP degradation [16,46,47,48,49,50,51,52,53,54,55,56,57,58,59]. Consequently, biomaterials capable of responding selectively to these pathological triggers may substantially improve therapeutic precision, local retention, and antibiofilm efficacy while minimizing systemic exposure and off-target toxicity [148,149,150,151].

From a translational standpoint, the principal platform classes are best compared along three axes: release control, biofilm penetration, and manufacturability. Sustained-release systems such as electrospun nanofibers and equilibrium-swollen hydrogels provide prolonged, predictable exposure and are technologically mature and scalable, but they release peptide regardless of microbial burden and may therefore expose healthy tissue to unnecessary concentrations and impose continuous selective pressure [140]. On-demand systems such as pH-, ROS-, or protease-responsive smart hydrogels instead concentrate release at the site and moment of infection, widening the therapeutic window at the cost of greater formulation complexity and trigger variability [148,149,150,151]. Among nanoscale carriers, liposomes offer high biocompatibility and the capacity to co-encapsulate hydrophilic peptides with adjuvants but suffer from limited storage stability and burst leakage, whereas polymeric nanoparticles afford tunable, often more sustained release and superior shelf stability at the expense of more demanding synthesis and potential excipient toxicity [144,145]; metallic–nanoparticle hybrids add intrinsic antimicrobial and ROS-amplifying activity but raise nanotoxicity and regulatory concerns [147]. No single platform is optimal across all axes, and the appropriate choice is dictated by wound type, microbial burden, required duration of action, and regulatory pathway.

These platforms convincingly demonstrate trigger selectivity in vitro; what remains under-reported is whether the triggered dose reliably exceeds the minimum biofilm-eradication concentration in vivo.

### 7.1. Stimuli-Responsive AMP Delivery Systems

Stimuli-responsive biomaterials are designed to undergo physicochemical or structural changes in response to wound-specific pathological triggers, thereby enabling controlled AMP release at infected or inflamed sites [133,134,148]. Such systems represent a major advance over conventional passive dressings because they directly integrate chronic wound pathophysiology into therapeutic design.

#### 7.1.1. pH-Responsive AMP Systems

Chronic infected wounds frequently exhibit alkaline pH values because of bacterial metabolism, proteolytic activity, tissue necrosis, and persistent inflammation [47,55]. This altered pH environment has stimulated development of pH-responsive AMP hydrogels and nanomaterials capable of releasing antimicrobial agents selectively within infected wound beds [133,148].

Recent peptide-based pH-sensitive hydrogels have demonstrated promising antibiofilm and regenerative properties in diabetic wound models. Fan et al. developed a peptide-based pH-responsive hydrogel capable of sustained AMP release within drug-resistant biofilm-infected diabetic wounds, resulting in enhanced bacterial eradication, reduced inflammation, and accelerated wound closure [131]. Mei et al. used a pH-responsive antibacterial and antioxidant peptide hydrogel to achieve rapid hemostasis and accelerated repair of infected wounds [149].

pH-responsive systems additionally improve peptide stability by reducing premature release and protecting AMPs from protease-rich exudates [17,148]. Importantly, these biomaterials may also function as diagnostic-responsive platforms capable of adapting therapeutic release according to infection severity and wound status.

#### 7.1.2. ROS-Responsive AMP Systems

Excessive ROS accumulation is a major pathological feature of chronic wounds and contributes to tissue injury, senescence, inflammatory amplification, and impaired healing [52,53]. ROS-responsive biomaterials therefore represent an attractive strategy for simultaneously modulating oxidative stress and controlling AMP delivery.

Several recent multifunctional hydrogels integrate antioxidative and antibiofilm properties into responsive wound platforms. Pranantyo et al. developed hydrogel dressings with intrinsic antibiofilm and antioxidative dual functionality that accelerated infected diabetic wound healing through ROS modulation, bacterial suppression, and improved tissue repair [132]. ROS-cleavable linkers and oxidation-sensitive polymeric networks may further enable selective AMP release within highly inflamed wound environments characterized by oxidative imbalance [150].

Importantly, ROS-responsive systems may simultaneously protect peptides from oxidative degradation while reducing local oxidative tissue damage and promoting regenerative signaling [52,53,149]. This dual-function therapeutic approach is particularly relevant in diabetic wounds, where persistent oxidative stress represents a central driver of chronicity [46,52].

#### 7.1.3. Enzyme-Responsive AMP Systems

Protease-rich wound exudates constitute one of the major barriers limiting free AMP stability and therapeutic efficacy [16,48]. Consequently, enzyme-responsive biomaterials capable of selective activation in the presence of bacterial proteases or wound-associated MMPs have emerged as highly sophisticated therapeutic platforms.

Böhner et al. recently developed bio-inspired biomaterial coatings enabling enzyme-responsive release of AMPs under pathological conditions [151]. Such systems exploit elevated protease activity within infected wounds to trigger localized peptide liberation specifically in regions of high bacterial burden or inflammatory activity. Matrix-cleavable peptide linkers may additionally permit selective therapeutic activation while minimizing premature peptide loss during storage or non-infected wound phases [151].

Enzyme-responsive systems are particularly attractive because they directly integrate one of the defining pathological hallmarks of chronic wounds—proteolytic dysregulation—into biomaterial functionality [16,48,151].

#### 7.1.4. Multi-Responsive and Adaptive AMP Platforms

Because chronic wounds exhibit simultaneous abnormalities involving pH, ROS, proteases, temperature, hypoxia, and inflammation, increasing attention has shifted toward multi-responsive biomaterials capable of integrating multiple pathological signals simultaneously [150,152].

Composite dual-responsive hydrogels have demonstrated promising performance through coordinated modulation of antimicrobial release, mechanical properties, moisture retention, and biofilm penetration [150,152]. Guerrero et al. designed a dual pH/enzyme-responsive hydrogel that releases the cationic peptide MP-L [I5R8] on demand, suppressing biofilms of drug-resistant wound bacteria in a preclinical infected-wound model [134]. Zhang et al. reported a pH/ROS dual-responsive peptide hydrogel that released its AMP selectively within the acidic, oxidative infected microenvironment while scavenging ROS and modulating the immune microenvironment to improve chronic-wound closure [148]. An ultrasound-responsive hydrogel carrying self-assembled human β-defensin (HBD)-peptide nanoparticles achieved deeper (~400 µm) wound penetration upon ultrasonic activation, drove complete wound closure in diabetic mice, and raised the angiogenic markers VEGF-A, CD31, and α-SMA [153]. Likewise, smart antibiofilm hydrogels engineered with synthetic AMPs increasingly combine responsive release with regenerative and immunomodulatory functions [154].

These adaptive systems may ultimately enable precision wound therapeutics capable of dynamically responding to evolving wound conditions throughout different stages of chronic wound progression. These platforms convincingly demonstrate trigger selectivity in vitro; what remains under-reported is whether the triggered dose reliably exceeds the minimum biofilm-eradication concentration in vivo.

### 7.2. Hybrid AMP Nanoplatforms

Nanotechnology-based AMP delivery systems have emerged as major translational tools for improving peptide stability, biofilm penetration, antimicrobial potency, and controlled release [144,145,155]. Unlike free peptides, AMP-loaded nanoplatforms may protect cargo from enzymatic degradation, improve retention within wound tissues, and enhance interaction with bacterial membranes and EPS matrices [17,144,155].

#### 7.2.1. AMP–Metal Nanoparticle Systems

Hybrid systems combining AMPs with metallic nanoparticles exhibit synergistic antimicrobial and antibiofilm activity through membrane disruption, ROS amplification, metal-ion release, and EPS destabilization [155,156]. Silver nanoparticles remain among the most extensively investigated antimicrobial nanomaterials because of their broad-spectrum activity and biofilm-disruptive properties [94,155]. Gold nanoparticle–AMP conjugates additionally demonstrate enhanced stability, targeted delivery, and prolonged antimicrobial activity while reducing peptide degradation [156]. Zinc oxide and copper nanoparticles may further contribute antimicrobial and pro-regenerative effects through ROS-mediated bacterial killing and modulation of inflammatory signaling [155].

Importantly, metal nanoparticle hybrids may improve AMP penetration into mature biofilms while simultaneously lowering the peptide concentrations required for effective microbial eradication [155,156].

#### 7.2.2. AMP–Polymeric Nanoparticle Systems

Polymeric nanoparticles, including chitosan, PLGA, dendrimers, micelles, and lipid–polymer hybrids, are increasingly employed to optimize AMP pharmacokinetics and wound-site retention [144,145,157]. These systems may enhance controlled release, improve peptide solubility, reduce cytotoxicity, and facilitate penetration into polymicrobial biofilms [144,157].

Recent translational reviews emphasize that advanced nanocarriers combined with molecular peptide modifications represent key strategies for overcoming clinical delivery barriers associated with AMP therapy [157]. Similarly, AMP-integrated nanoparticle systems capable of synergistic combination therapy with antibiotics, peptidomimetics, photodynamic agents, or immunomodulators may further improve chronic wound outcomes [158].

#### 7.2.3. Self-Assembled AMP Nanostructures

Self-assembling antimicrobial peptides represent a particularly innovative area of biomaterial engineering because peptides themselves function simultaneously as therapeutic agents and structural biomaterials [159]. These systems spontaneously organize into nanofibers, nanogels, supramolecular assemblies, or peptide amphiphile structures capable of sustained antimicrobial activity and biofilm disruption [159].

Self-assembled nanostructured AMPs exhibit tunable physicochemical properties, improved stability, and enhanced multifunctionality while reducing the need for additional carrier materials [159]. Furthermore, supramolecular peptide hydrogels capable of disrupting polymicrobial biofilms and accelerating infected wound healing have demonstrated substantial promise in recent experimental studies [135].

### 7.3. Regenerative Multifunctional AMP Systems

An important evolution in AMP biomaterials involves integration of regenerative therapeutics with antimicrobial and antibiofilm functionality. Chronic wounds require simultaneous control of infection, inflammation, vascular dysfunction, oxidative stress, and impaired tissue repair [46,47,48,49,50,51,52,53,54,55,56,57,58,59]. Consequently, multifunctional biomaterials increasingly combine AMPs with regenerative mediators such as growth factors, exosomes, stem-cell secretomes, angiogenic agents, or antioxidative components [14,15,149].

AMP–growth factor co-delivery systems may synergistically promote bacterial eradication while enhancing angiogenesis, fibroblast activation, collagen deposition, and re-epithelialization [14,15]. Likewise, AMP–exosome or AMP–secretome platforms are attracting growing interest because extracellular vesicles may provide additional immunomodulatory and regenerative signaling while supporting wound healing [14,15].

Recent multifunctional composite wound dressings integrating AMP-modified fluorescence-responsive systems have additionally demonstrated simultaneous antimicrobial, regenerative, and diagnostic capabilities [160]. Such integrated systems represent an important step toward precision wound therapeutics capable of dynamically adapting to wound progression and infection status.

### 7.4. Electroactive and Conductive AMP Biomaterials

Electroactive wound dressings represent another emerging frontier in chronic wound therapeutics. Endogenous bioelectric fields play important roles in keratinocyte migration, fibroblast behavior, angiogenesis, and tissue repair [37,40]. Conductive biomaterials may therefore enhance regenerative healing while simultaneously supporting AMP delivery.

Conductive hydrogels, electroactive nanofibers, and electrically responsive scaffolds have demonstrated promising effects on bacterial eradication, cell migration, and wound closure [17,19]. These systems may additionally improve controlled peptide release and facilitate biofilm penetration through electrostatic interactions with microbial membranes and EPS matrices [155].

Although still relatively early in development, electroactive AMP platforms may become particularly relevant for ischemic diabetic wounds characterized by severe vascular dysfunction, impaired electrical signaling, and persistent biofilm-associated inflammation.

### 7.5. Smart Biosensing and Theranostic AMP Dressings

One of the most advanced developments in chronic wound therapeutics involves integration of sensing and therapeutic functions within a single platform. Smart biosensing dressings are increasingly engineered to detect infection-associated biomarkers, including pH changes, bacterial toxins, proteases, ROS levels, inflammatory mediators, and biofilm metabolites [150,160].

Theranostic AMP systems combine real-time wound monitoring with responsive antimicrobial release, thereby enabling adaptive treatment according to evolving wound conditions [160]. Fluorescence-responsive dressings, electrochemical sensors, and colorimetric infection indicators may provide clinicians with immediate information regarding wound status while simultaneously activating local AMP release [160].

Importantly, these technologies align closely with emerging concepts of precision wound medicine and individualized chronic wound management. Future systems may incorporate wireless monitoring, AI-assisted wound analysis, and closed-loop therapeutic regulation capable of continuously adapting AMP release according to wound microenvironmental changes.

### 7.6. Remaining Translational Challenges of Smart AMP Biomaterials

Despite major advances, smart AMP-responsive biomaterials continue to face substantial translational barriers, including manufacturing complexity, scalability, sterilization stability, regulatory uncertainty, reproducibility issues, and cost [17,157]. In addition, many experimental studies still rely on simplified mono-species biofilms and acute wound models that inadequately reproduce the complexity of human chronic wounds [129,130].

Moreover, excessive formulation complexity may complicate regulatory approval and large-scale clinical implementation. Balancing responsiveness, peptide stability, biocompatibility, mechanical integrity, and manufacturability therefore remains a major engineering challenge [17,18,19,157].

Nevertheless, pathology-responsive AMP biomaterials represent one of the most promising directions for next-generation chronic wound therapy because they directly integrate antimicrobial action, biofilm disruption, immune modulation, and regenerative support into unified therapeutic platforms. Representative pathology-responsive systems are inventoried in Table 3.

**Figure 8:**
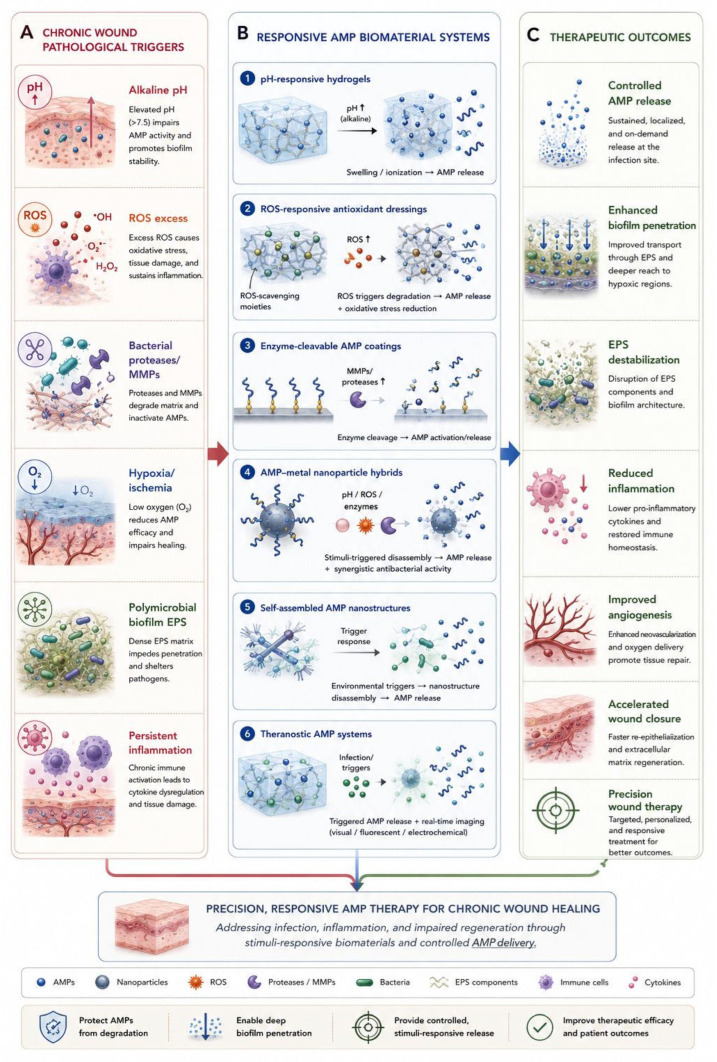
Smart AMP biomaterials responsive to pathology for chronic wound therapy. This figure presents the conceptual trigger–release–effect logic shared by these systems and is intended to complement, rather than duplicate, the referenced inventory of specific platforms and citations provided in Table 3. (**A**) Chronic wound pathological triggers. Six features of the chronic wound microenvironment that impair healing and AMP activity: alkaline pH (>7.5), excess ROS, bacterial proteases/MMPs, hypoxia/ischemia, dense polymicrobial biofilm EPS, and persistent inflammation. (**B**) Responsive AMP biomaterial systems. Six stimuli-responsive delivery platforms that exploit these triggers for on-demand AMP release: pH-responsive hydrogels, ROS-responsive antioxidant dressings, enzyme-cleavable coatings, AMP–metal nanoparticle hybrids, self-assembled AMP nanostructures, and theranostic systems combining release with real-time imaging. (**C**) Therapeutic outcomes. The resulting benefits: controlled AMP release, enhanced biofilm penetration, EPS destabilization, reduced inflammation, improved angiogenesis, accelerated wound closure, and precision wound therapy—together delivering responsive AMP treatment that addresses infection, inflammation, and impaired regeneration. Abbreviations: AMP, antimicrobial peptide; EPS, extracellular polymeric substance; MMP, matrix metalloproteinase; QS, quorum sensing; ROS, reactive oxygen species.

Figure 8 illustrates the mechanistic integration of stimuli responsive antimicrobial peptide (AMP)-enabled biomaterials within the hostile microenvironment of chronic wounds. Panel A highlights the main pathological triggers characteristic of non-healing wounds: alkaline pH, elevated ROS, bacterial proteases/matrix metalloproteinases (MMPs), hypoxia/ischemia, polymicrobial EPS-rich biofilms and chronic inflammation. These pathological conditions collectively compromise the stability of endogenous AMPs, sustain microbial persistence and perpetuate inflammatory tissue damage.

Advanced responsive AMP biomaterial systems that aim to exploit these pathological stimuli for controlled therapeutic activation and delivery are presented in Panel B. pH-responsive hydrogels undergo swelling and AMP release due to ionization under alkaline wound conditions. ROS-responsive antioxidative dressings combine AMP delivery and oxidative stress scavenging. Enzyme-cleavable AMP coatings release active peptides upon degradation of linkers by bacterial proteases/MMPs. AMP–metal nanoparticle hybrids provide synergistic antimicrobial activity, enhanced biofilm penetration and stimulus-induced payload disassembly. Self-assembled AMP nanostructures undergo dynamic disassembly in response to pathological wound conditions, allowing localized peptide release. Theranostic AMP systems combine responsive AMP delivery with concurrent infection sensing and real-time wound monitoring via fluorescent, visual or electrochemical outputs.

Panel C summarizes the resulting therapeutic effects, including sustained and localized AMP release, improved penetration into mature polymicrobial biofilms, EPS destabilization, reduced inflammation, enhanced angiogenesis, accelerated wound closure, and precision wound therapy. Overall, pathology-responsive AMP biomaterials address major translational barriers associated with free AMP delivery by protecting peptides from degradation, improving retention within the wound microenvironment, and enabling spatiotemporally controlled therapeutic activation.

## 8. Translational Evidence and Clinical Progress of AMP-Enabled Antibiofilm Systems

Despite the rapidly expanding development of antimicrobial peptide (AMP)-enabled biomaterials, successful clinical translation ultimately requires evidence that extends beyond antimicrobial potency alone. For chronic biofilm-infected wounds, clinically meaningful performance should include activity against planktonic and biofilm-associated microorganisms, cytocompatibility, regenerative support, stability in wound-like environments, and reproducibility in models that reflect chronic wound complexity [17,18,19,119,130]. Current evidence suggests that AMP-loaded hydrogels, nanofibers, nanoparticles, coatings, and smart dressings can improve peptide stability, local retention, antibiofilm activity, and tissue repair compared with free AMPs [131,132,133,134,135,136,137,138,139,140,142,143,144,145,146,147,148,149,150,151,152,154,155,156,157,158,159,160]. However, the translational pathway remains limited by simplified in vitro assays, insufficiently mature or polymicrobial biofilm models, incomplete safety profiling, and the scarcity of advanced preclinical and clinical studies [119,129,130].

### 8.1. In Vitro Evidence for AMP-Based Antibiofilm Systems

#### 8.1.1. Planktonic Antimicrobial Activity

Early evaluation of AMP-based wound therapeutics usually begins with planktonic antimicrobial susceptibility assays, including minimum inhibitory concentration (MIC), minimum bactericidal concentration (MBC), time-kill kinetics, membrane-permeabilization assays, and bacterial viability testing [100,101,102,103,108,109]. These assays provide useful initial information regarding peptide potency against clinically relevant wound pathogens, including methicillin-resistant *Staphylococcus aureus* (MRSA), *Pseudomonas aeruginosa*, *Enterococcus faecalis*, and *Acinetobacter baumannii* [78,79,80,81,82,83,84,85,86,87,88,89,90].

AMPs are particularly attractive in this context because many act through rapid membrane disruption, intracellular targeting, metabolic collapse, or ROS-associated bacterial injury rather than through classical antibiotic targets [100,107,108,109,110]. This mechanism partially explains why several AMPs retain activity against multidrug-resistant pathogens that are no longer susceptible to conventional antibiotics [13,14,15,17]. Biomaterial-assisted delivery can further enhance this activity by maintaining local peptide concentrations, reducing degradation, and improving contact with microbial membranes [131,132,133,134,135,137,138,139,140,144,145,146,147].

Nevertheless, planktonic testing alone has limited predictive value for chronic wound therapy. Biofilm-associated bacteria differ substantially from planktonic cells in growth rate, metabolic state, gene expression, stress adaptation, and antimicrobial tolerance [60,61,64,65,66,67,68,69,70,71]. Therefore, MIC/MBC results should be interpreted as preliminary screening data rather than direct indicators of antibiofilm or clinical efficacy [119,129].

#### 8.1.2. Antibiofilm Efficacy

Antibiofilm testing is more relevant for chronic wound applications because mature biofilms represent one of the principal causes of persistent infection and impaired healing [6,7,47,60,61,64,65]. AMP-enabled systems may inhibit early adhesion, prevent microcolony formation, destabilize EPS, interfere with quorum sensing, disrupt bacterial membranes, and target metabolically tolerant subpopulations [60,61,64,65,66,67,68,69,70,71,111,113,114,116,117,118].

Importantly, antibiofilm assays should distinguish between prevention of biofilm formation and eradication of mature biofilms. Many AMP systems show strong activity against early-stage or immature biofilms, but mature biofilms are substantially more difficult to eradicate because of dense EPS architecture, oxygen and nutrient gradients, persister-cell enrichment, and altered microbial metabolism [60,61,64,65,66,67,68,69,70,71]. Consequently, studies using short-incubation mono-species biofilms may overestimate translational relevance compared with models using mature polymicrobial biofilms derived from clinically relevant wound pathogens [72,73,74,75,76,129].

Recent biomaterial studies increasingly address these limitations. Mukherjee et al. developed antimicrobial supramolecular nanocomposite hydrogels in which silver and gold nanoparticles are grown in situ around short self-assembling peptides; these achieved a ≥1.5-log_10_ reduction in microbial viability against mono- and polymicrobial biofilms, outperforming a last-resort antibiotic and a commercial silver ointment, and accelerated healing of infected wounds in vivo by lowering bacterial burden [135]. Metwally et al. loaded the synthetic antimicrobial peptide EM86 into gamma-irradiated sodium-alginate/poly(vinyl alcohol) electrospun nanofibers, which provided sustained local activity against multidrug-resistant *P. aeruginosa* wound infections in vivo [140]. In a complementary primary study, a methylcellulose hydrogel loaded with the designed peptide D-Bac8c^2,5^Leu produced a 2–3-log_10_ reduction in viable load across mono- and polymicrobial *S. aureus* and *P. aeruginosa* biofilms in both static and flow models, retained low cytotoxicity toward human cells, and disrupted pre-formed biofilms [161]. Similarly, smart AMP hydrogels and nanomaterial-based systems have been designed to enhance biofilm penetration, prolong AMP exposure, and improve local therapeutic concentrations [131,132,133,134,135,137,138,139,140,144,145,146,147,148,149,150,151,152,154,155,156,157,158,159,160]. In our assessment, the few systems reporting standardized log_10_ CFU reductions, such as the ≥1.5-log reduction achieved by the nanocomposite hydrogel [135], show meaningful but generally sub-eradication activity, which reinforces the view that delivery and dosing, rather than peptide discovery, are now the rate-limiting steps.

However, standardization remains a major limitation. As detailed in Section 5.7, biofilm studies differ widely in strain selection, incubation time, matrix maturity, and endpoints, which limits cross-study comparison [119,129,130].

The few studies reporting standardized log10 CFU reductions (for example ≥ 1.5-log for the nanocomposite hydrogel [135] and 2–3-log for the D-Bac8c hydrogel [161]) indicate meaningful but generally sub-eradication activity, underscoring why delivery and dosing, rather than peptide discovery, are now rate-limiting.

#### 8.1.3. Cytocompatibility and Regenerative Activity

For wound therapy, antimicrobial efficacy must be balanced with host–cell compatibility and regenerative benefit. In vitro evaluation should therefore include keratinocyte viability and migration, fibroblast proliferation, endothelial-cell activity, macrophage polarization, hemocompatibility, and inflammatory cytokine modulation [14,15,20,33,34]. This is particularly important because excessive AMP hydrophobicity or membrane activity may increase hemolysis, keratinocyte toxicity, fibroblast injury, or irritation at higher concentrations [99,100,101,102,103].

AMP-enabled biomaterials may improve safety by reducing burst exposure, enabling sustained release, and limiting direct toxicity to host cells [18,19,131,132,133,134,135]. Some systems also provide intrinsic regenerative activity. Hydrogels may preserve wound moisture, support cell migration, and absorb exudate; electrospun nanofibers may mimic extracellular matrix architecture; and smart dressings may integrate antioxidative, anti-inflammatory, or biosensing functions [131,132,133,134,135,136,137,138,139,140,142,143,144,145,146,147,148,149,150,151,152,154,155,156,157,158,159,160]. These properties are important because chronic wounds require simultaneous control of infection, inflammation, oxidative stress, and impaired tissue repair [46,47,48,49,50,51,52,53,54,55,56,57,58,59].

### 8.2. In Vivo and Preclinical Wound Models

#### 8.2.1. Acute Infected Wound Models

Murine excisional and burn wound models remain widely used for initial in vivo evaluation of AMP-enabled systems because they are accessible, cost-effective, and experimentally reproducible [35,130]. These models commonly involve inoculation with MRSA, *P. aeruginosa*, or other wound pathogens, followed by topical application of AMP-loaded hydrogels, nanofibers, nanoparticles, or composite dressings [131,132,133,134,135,137,138,139,140].

Such studies are useful for assessing bacterial reduction, wound closure rate, inflammatory response, histological repair, collagen deposition, and preliminary safety. However, acute murine wounds do not fully reproduce human chronic wound biology. Rodent wounds heal largely through contraction, whereas human wounds depend more strongly on granulation tissue formation and re-epithelialization [35,130]. In addition, many acute models lack diabetes, ischemia, vascular dysfunction, senescence, recurrent infection, and mature polymicrobial biofilms [46,47,48,49,50,51,52,53,54,55,56,57,58,59,130].

#### 8.2.2. Diabetic and Ischemic Wound Models

Diabetic and ischemic models are more translationally relevant for chronic wound research because they reproduce delayed closure, impaired angiogenesis, oxidative stress, inflammation, and defective immune responses [46,47,48,49,50,51,52,53,54,55,56,57,58,59]. Common models include streptozotocin-induced diabetes, genetically diabetic db/db mice, ischemic flap models, and infected diabetic wound models [130,162].

Several AMP-enabled systems have shown promising activity in diabetic wound settings. Fan et al. constructed a peptide-based pH-sensitive antibacterial hydrogel that released its cationic peptide selectively under the acidic conditions of the infected wound bed, eradicated drug-resistant bacteria in vitro and in vivo, and significantly accelerated closure of biofilm-infected diabetic wounds while retaining good biocompatibility [131]. Antioxidative and antibiofilm hydrogel dressings have accelerated infected diabetic wound repair [132]. Da Silva et al. delivered human β-defensin-2 from calcium-cross-linked alginate hydrogels with a nanometric porous structure in a streptozotocin-diabetic mouse model bearing MRSA-infected wounds; treatment increased the numbers of Ki67-positive (proliferating) and CD31-positive (endothelial) cells and exerted antimicrobial, anti-inflammatory, and pro-angiogenic effects, supporting an early progression toward the resolution phase of healing [162,163]. In a related design, a carrier-free dual-functional hydrogel co-assembled from the antimicrobial peptide Jelleine-1 and the membrane-permeable cyclic-AMP analogue 8Br-cAMP cleared bacterial burden and accelerated closure of MRSA-infected diabetic wounds, illustrating how the peptide can act simultaneously as the antimicrobial agent and the structural matrix [164].

These models are especially valuable because they allow simultaneous evaluation of antimicrobial activity and regenerative outcomes, including re-epithelialization, angiogenesis, collagen organization, inflammatory-cell infiltration, and bacterial burden. However, even diabetic mouse models often fail to reproduce the full chronicity, vascular insufficiency, neuropathy, and polymicrobial ecology of human diabetic foot ulcers [4,5,130,162].

#### 8.2.3. Polymicrobial and Mature Biofilm Models

Polymicrobial and mature biofilm models are essential for assessing AMP-enabled systems intended for chronic wounds. Human chronic wounds frequently contain complex microbial communities involving *S. aureus*, *P. aeruginosa*, enterococci, anaerobes, and fungi, embedded within EPS and host-derived matrix components [72,73,74,75,76]. These communities display interspecies cooperation, metabolic heterogeneity, quorum-sensing interactions, and enhanced antimicrobial tolerance [60,61,64,65,66,67,68,69,70,71,72,73].

Compared with mono-species models, polymicrobial systems better reproduce clinical biofilm behavior and may reveal therapeutic limitations that are not apparent in simplified assays. For example, *P. aeruginosa* and *S. aureus* interactions can alter virulence, antibiotic susceptibility, respiratory metabolism, and small-colony variant formation [72,73]. Transcriptomic approaches have further shown that wound biofilms undergo dynamic molecular adaptation, reinforcing the need for biofilm models that capture microbial physiology rather than biomass alone [129].

Therefore, future AMP studies should include clinically derived isolates, mature biofilm incubation periods, recurrent inoculation models, and wound-like matrices. Assays should measure not only biomass reduction but also viable bacterial burden, EPS disruption, persister survival, inflammatory modulation, and recurrence after treatment withdrawal.

#### 8.2.4. Large-Animal and Translational Models

Large-animal models, particularly porcine wound models, are increasingly regarded as highly valuable translational systems because porcine skin more closely resembles human skin in epidermal thickness, dermal architecture, collagen organization, hair density, vascularization, and healing physiology [130,165]. Porcine models also allow evaluation of dressing adherence, exudate management, wound coverage, mechanical durability, and repeated clinical-style dressing changes.

However, large-animal models remain underused because they require specialized facilities, higher costs, greater ethical oversight, and more complex wound-care protocols [165]. Recent reviews emphasize that improving chronic wound models requires inclusion of diabetes, ischemia, infection, biofilm formation, impaired angiogenesis, and delayed re-epithelialization rather than reliance on simple acute wounds [130,162,165,166]. For AMP-enabled biomaterials, such models may be particularly important because local retention, release kinetics, degradation, toxicity, and dressing performance are strongly influenced by wound size, exudate production, tissue depth, and host physiology.

### 8.3. Clinical Translation of AMP-Based Wound Therapeutics

Clinical translation of AMP-based wound therapeutics remains limited despite substantial preclinical progress [17,119]. The best-known clinical-stage AMP for wound infection is pexiganan, a synthetic magainin analogue evaluated as a topical antimicrobial for mildly infected diabetic foot ulcers [167,168]. In a randomized, controlled, double-blind multicenter trial, topical pexiganan was compared with systemic antibiotic therapy for mild diabetic foot ulcer infection, demonstrating the feasibility of topical AMP therapy but also highlighting the challenges of regulatory approval and clinical positioning [167]. Importantly, pexiganan has not received regulatory approval as a wound therapeutic. The U.S. Food and Drug Administration declined approval in 1999 after two phase III trials showed no superiority over existing oral therapy, and the more recent OneStep-1 and OneStep-2 phase III trials (completed in 2016) likewise failed to meet their primary efficacy endpoints. Its clinical-stage status should therefore not be interpreted as evidence of demonstrated efficacy or regulatory approval, and earlier favourable reports must be read in light of these later negative trials [169].

LL-37-based therapy has also been investigated clinically for hard-to-heal venous leg ulcers. In a first-in-man randomized, placebo-controlled trial in 34 patients with hard-to-heal venous leg ulcers, topical LL-37 at 0.5 and 1.6 mg/mL (applied twice weekly) raised the healing-rate constant approximately six- and three-fold over placebo (*p* = 0.003 at 0.5 mg/mL), whereas the highest dose (3.2 mg/mL) conferred no benefit, indicating a narrow therapeutic window [170]. The subsequent larger phase IIb HEAL LL-37 trial (148 patients), however, did not demonstrate significant superiority over compression therapy [171]. As with pexiganan, this progression from an encouraging early-phase signal to a non-confirmatory larger trial tempers expectations for free-peptide topical therapy and reinforces the rationale for the delivery-enabled formulations developed in Section 6 and Section 7. However, clinical development remains complicated by peptide stability, dose optimization, proteolytic degradation, formulation requirements, and patient heterogeneity [170,172,173].

Other topical AMPs, such as omiganan, have been clinically evaluated in dermatological conditions and skin microbiome modulation, although not necessarily as chronic wound biofilm therapies [174,175]. These studies remain informative because they highlight the broader clinical issues affecting topical peptide development, including local tolerability, microbiome effects, endpoint selection, and formulation performance [174,175].

Overall, relatively few AMP-based wound products have reached advanced clinical stages. Barriers include peptide instability, manufacturing cost, short half-life, uncertain reimbursement, lack of standardized wound endpoints, trial heterogeneity, and regulatory complexity [17,119]. Moreover, chronic wound trials must account for vascular status, glycemic control, wound duration, offloading, debridement practices, microbial burden, biofilm maturity, and concomitant therapies [91,92,93,94]. Consequently, future clinical trials should evaluate AMP-enabled systems using clinically meaningful endpoints such as complete wound closure, time to closure, infection recurrence, antibiotic-sparing effect, limb salvage, pain reduction, safety, and quality of life.

### 8.4. Comparative Translational Assessment of AMP Biomaterial Systems

The growing diversity of AMP-enabled wound biomaterials highlights the need for comparative translational assessment. No single platform currently addresses all barriers associated with chronic biofilm-infected wounds. Hydrogels offer moisture retention, local AMP delivery, and wound-bed conformability, but may suffer from weak mechanical properties or burst release [18,131,132,133,134,135]. Electrospun nanofibers provide extracellular matrix mimicry and sustained release, but scale-up and sterilization may be challenging [137,138,139,140]. Nanoparticles improve peptide stability and biofilm penetration, but may raise concerns regarding toxicity, biodistribution, and regulatory classification [144,145,146,147]. Smart systems offer pathology-responsive release and theranostic potential but increase formulation complexity and regulatory uncertainty [148,149,150,151,152,154,155,156,157,158,159,160].

A useful translational comparison should therefore integrate antimicrobial efficacy, antibiofilm activity, regenerative effect, model relevance, clinical stage, and primary bottleneck. This approach prevents overinterpretation of promising in vitro results and helps identify which systems are most likely to progress toward clinical application. Representative systems, their mechanism-resolved properties, and the primary in vivo and clinical evidence are compiled in Table 4, Table 5 and Table 6, respectively.

To enable direct comparison across heterogeneous studies, Table 5 resolves representative AMP systems by bacterial target, peptide class, trigger mechanism, specific antibiofilm mode of action, and reported quantitative endpoint. Notably, standardized biofilm metrics such as log10 CFU reduction or the minimum biofilm eradication concentration are reported for only a minority of systems, which limits quantitative cross-study comparison and reinforces the need for the standardized antibiofilm endpoints discussed in Section 8.1.2.

Beyond comparing individual systems, the experimental models that generate this evidence differ substantially in physiological fidelity and translational weight; their relative strengths and limitations are summarized in Table 7.

Taken together, the comparative data in Table 4 and Table 5 reveal three consistent patterns. First, quantitative antibiofilm endpoints (log10 CFU reduction or minimum biofilm-eradication concentration) are reported for only a minority of systems, so apparent differences between platforms partly reflect reporting heterogeneity rather than true efficacy gaps. Second, the systems with the most complete evidence chains—a defined peptide, an infected-diabetic in vivo model, and a measured bacterial-load reduction—are concentrated among self-assembling and nanoparticle-hybrid hydrogels, whereas films and porous scaffolds remain largely characterized in vitro. Third, no platform yet couples demonstrated mature-polymicrobial-biofilm eradication with validated tissue regeneration in a single, clinically representative model; in our view this, rather than a shortage of candidate peptides, defines the field’s principal evidence gap.

## 9. Future Perspectives: Artificial Intelligence, Precision Biomaterials and Next-Generation AMP Therapeutics

The future development of antimicrobial peptide (AMP)-enabled wound therapeutics will likely depend on the convergence of computational peptide engineering, pathology-responsive biomaterials, precision wound diagnostics, and clinically relevant translational models. Although AMPs possess broad antimicrobial, antibiofilm, immunomodulatory, and regenerative potential, peptide discovery alone is insufficient to overcome the biological complexity of chronic biofilm-infected wounds [13,14,15,16,17,18,19]. Future strategies must therefore move beyond empirical screening toward integrated systems capable of optimizing peptide sequence, delivery platform, wound microenvironment responsiveness, and patient-specific therapeutic requirements simultaneously.

### 9.1. Artificial Intelligence-Assisted AMP Discovery and Optimization

Artificial intelligence (AI) is increasingly transforming AMP development from empirical peptide screening toward data-driven rational therapeutic design. Machine learning (including classical supervised classifiers such as random forests and support-vector machines), deep learning, protein language models, graph neural networks, variational autoencoders, generative adversarial networks, diffusion models, and transformer-based architectures are now being used to identify novel AMP candidates, predict antimicrobial activity, estimate toxicity, optimize physicochemical properties, and prioritize candidates for experimental validation [175,176,177,178,179,180]. These approaches are particularly valuable because AMP sequence space is vast, while conventional trial-and-error screening remains time-consuming, expensive, and poorly suited for simultaneous optimization of antimicrobial potency, cytocompatibility, protease resistance, and antibiofilm activity.

A growing body of original studies, rather than reviews alone, now documents concrete AI applications in AMP sequence discovery, activity prediction, and optimization, and it is useful to separate these validated uses from the still-speculative role of AI in peptide–carrier co-design. The clearest validated application is large-scale discovery: machine-learning mining of global microbiome and metagenomic datasets has expanded the known AMP space by orders of magnitude, with the AMPSphere resource cataloguing on the order of one million prokaryotic candidate peptides, a subset of which were chemically synthesized and shown to inhibit clinically relevant pathogens [175]. Beyond discovery, sequence-based classifiers and quantitative structure–activity relationship (QSAR) models can prioritize candidates before synthesis; in a directly wound-relevant example, Haney et al. built three-dimensional QSAR models from a SPOT-synthesized library of IDR-1018 variants, screened 100,000 virtual sequences for antibiofilm activity with approximately 85% predictive accuracy, and identified peptide 3002, which showed an eight-fold increase in antibiofilm potency against methicillin-resistant *Staphylococcus aureus* (MRSA) in vitro and reduced abscess size in a chronic MRSA mouse model [181].

Generative models move beyond prioritization to propose entirely new sequences. Das et al. coupled a deep generative autoencoder (controlled latent attribute space sampling, CLaSS) with deep-learning classifiers and high-throughput molecular-dynamics features, synthesizing only twenty of roughly ninety thousand generated peptides to obtain two minimalist AMPs potent against Gram-positive and Gram-negative pathogens, including multidrug-resistant *Klebsiella pneumoniae*, with low hemolysis and low in vivo toxicity in mice [182]. Pandi et al. combined variational-autoencoder design and convolutional or recurrent minimum-inhibitory-concentration regressors with a cell-free protein-synthesis screen, testing 500 of approximately 500,000 generated sequences and recovering 30 functional AMPs, six with broad-spectrum activity against multidrug-resistant pathogens, no detectable resistance induction, and minimal human-cell toxicity [183]. Related peptide-language and generative approaches have likewise produced de novo AMPs with validated activity against drug-resistant bacteria [176]. Genetic-algorithm optimization of natural templates is a further validated route: Porto et al. computationally evolved a guava-derived peptide to yield guavanin 2, an arginine-rich α-helical AMP with potent activity against Gram-negative wound-relevant pathogens such as *Acinetobacter baumannii* [184].

Critically for translation, several of these pipelines now optimize safety alongside potency. Capecchi et al. trained recurrent neural networks on paired activity and hemolysis data from the DBAASP database and, by synthesizing 28 sequences each at least five mutations from the training set, obtained eight new non-hemolytic AMPs active against *Pseudomonas aeruginosa*, *A. baumannii*, and MRSA—the three pathogens most relevant to chronic wound infection [185]. Together with the in vitro hemolysis and in vivo toxicity screening embedded in the CLaSS and cell-free pipelines [182,183], these results indicate that in silico hemolytic- and cytotoxicity-prediction is now sufficiently mature to deprioritize unsafe candidates before synthesis. These validated capabilities—database mining, activity and antibiofilm prediction, generative de novo design, template optimization, and toxicity filtering—are summarized in Table 8.

For chronic wound applications, AI-guided AMP development should prioritize properties that are often underrepresented in classical antimicrobial prediction pipelines. These include stability in protease-rich wound exudate, activity under physiological salt concentrations, low hemolytic potential, reduced keratinocyte and fibroblast toxicity, activity against mature polymicrobial biofilms, compatibility with hydrogels or nanofiber matrices, and preservation of immunomodulatory or regenerative properties [16,17,18,19,99,100,101,102,103]. Machine learning models trained only on planktonic MIC datasets may therefore be insufficient for chronic wound translation. Future predictive platforms should incorporate antibiofilm endpoints, wound-fluid stability, peptide–EPS interactions, and host–cell compatibility data.

By contrast, the application of AI to the delivery problem central to this review remains an emerging and largely unvalidated direction rather than an established capability. In principle, predictive models could estimate the affinity of a candidate peptide for a given carrier chemistry or biomaterial surface—for example, the strength of electrostatic and hydrophobic adsorption to a particular hydrogel polymer or nanoparticle coating—and thereby forecast loading efficiency and release behavior before any material is synthesized; integrating such affinity prediction with toxicity, antibiofilm, and wound-fluid-stability endpoints would in turn allow peptide and carrier to be co-designed computationally. We emphasize, however, that to our knowledge no published study has yet experimentally validated machine-learning prediction of peptide–carrier affinity, loading, or release for wound AMP systems, and these statements therefore describe a prospective opportunity rather than demonstrated evidence. The validated contribution of AI to formulation at present is narrower and indirect: in silico hemolytic- and mammalian-cell-toxicity classifiers reduce downstream attrition by filtering unsafe sequences before they enter formulation [182,183,185]. Positioned realistically, artificial intelligence is best regarded today as a connective tool spanning sequence design and translational risk assessment, with peptide–carrier co-design a clearly delineated future goal.

Several methodological limitations temper these advances and are particularly acute for wound applications. Public AMP datasets are markedly imbalanced, containing far more active than experimentally confirmed inactive sequences, so models frequently learn from scarce and unreliable negative data [178,179]. Activity labels are themselves inconsistent, because reported MIC values are aggregated across species, media, inocula, and laboratories, and the binary active/inactive thresholds used to train classifiers differ between databases. High sequence similarity between training and test peptides creates a substantial risk of train–test leakage, which inflates reported accuracy and overstates generalization to genuinely novel chemistries. Most models are additionally trained and benchmarked on planktonic MIC data, whereas validation under wound-like conditions—protease-rich exudate, physiological or elevated salt, acidic pH, host cells, and mature polymicrobial biofilms—remains rare, with the antibiofilm and in vivo confirmations described above [181,182] still the exception rather than the rule. A realistic appraisal of AI-guided AMP design therefore requires standardized, condition-resolved datasets, explicit similarity-aware data splits, and prospective experimental confirmation under chronic-wound-relevant conditions.

Molecular docking, molecular dynamics simulations, and membrane-interaction modeling may also assist AMP optimization by clarifying peptide orientation, bilayer insertion, pore formation, aggregation behavior, and interactions with extracellular DNA or polysaccharide-rich biofilm matrices [107,108,109,110,111,177]. Such structure–function analyses could support rational modification strategies, including cyclization, D-amino acid substitution, stapling, lipidation, terminal modification, and incorporation of non-natural amino acids, to improve peptide stability and selectivity [99,105,108,109].

### 9.2. Precision Biomaterials and Pathology-Responsive Wound Systems

Next-generation AMP therapy will likely depend not only on better peptide sequences, but also on precision biomaterials designed around chronic wound pathophysiology. Chronic wounds contain dynamic pathological triggers, including alkaline pH, excessive ROS, protease-rich exudate, hypoxia, biofilm EPS, ischemia, and persistent inflammation [46,47,48,49,50,51,52,53,54,55,56,57,58,59]. Smart biomaterials capable of responding to these triggers may improve therapeutic precision by releasing AMPs selectively in infected or inflamed regions while reducing premature peptide degradation and host–cell toxicity [131,132,133,134,135,136,137,138,139,140,142,143,144,145,146,147,148,149,150,151,152,154,155,156,157,158,159,160].

Future biomaterial systems should be designed as active therapeutic microenvironments rather than passive dressings. pH-responsive hydrogels may exploit infection-associated wound alkalinity, ROS-responsive systems may combine antimicrobial release with oxidative-stress modulation, and enzyme-responsive coatings may use bacterial proteases or MMP-rich exudates as triggers for localized peptide liberation [133,134,136,148,149,160]. Similarly, electroconductive hydrogels and biosensor-integrated dressings may support electrical stimulation, real-time monitoring, and adaptive therapy in ischemic diabetic wounds [150,151,156,157,158,159].

A particularly promising direction is the integration of AI-designed AMPs with biomaterials selected or optimized through computational approaches. In the future, peptide sequence design, hydrogel composition, nanoparticle charge, release kinetics, degradation profile, and wound-specific trigger responsiveness could be co-optimized using machine learning. Such platforms may enable personalized AMP dressings tailored to microbial composition, wound pH, protease burden, exudate level, vascular status, and inflammatory profile.

### 9.3. Personalized and Microbiome-Informed AMP Wound Therapy

Current chronic wound therapy often treats infection as a generalized microbial burden, yet wound microbiomes differ substantially between patients, wound types, anatomical sites, and stages of chronicity [74,75,76]. Future AMP-based strategies should increasingly consider microbiome-informed personalization. Rather than aiming for broad microbial eradication alone, next-generation systems may seek to suppress pathogenic biofilms while preserving or restoring beneficial wound microbiota [12].

This shift is especially relevant because excessive or repeated antimicrobial exposure may disrupt microbial ecology and impair healing [12,92]. Precision AMP therapy could eventually combine wound microbiome sequencing, resistance profiling, biofilm phenotyping, and AI-guided peptide selection to match specific AMP combinations with patient-specific microbial communities. Such approaches may be particularly useful for polymicrobial diabetic foot ulcers, where *S. aureus*, *P. aeruginosa*, enterococci, anaerobes, and fungi may coexist within complex biofilm ecosystems [72,73,74,75,76].

Multi-AMP systems may also become important. Rational peptide cocktails could combine membrane-disruptive AMPs, anti-quorum-sensing peptides, EPS-destabilizing peptides, immunomodulatory peptides, and pro-regenerative peptides within a single biomaterial platform. This modular approach could reduce reliance on a single mechanism of action and improve efficacy against heterogeneous biofilm populations.

### 9.4. Combination Strategies Beyond Conventional Antibiotics

Future AMP-enabled wound therapeutics will likely involve rational combination systems rather than monotherapy. AMP–antibiotic combinations may enhance bacterial killing, reduce required antibiotic doses, improve biofilm penetration, and restore susceptibility in resistant organisms [116,128]. Similarly, AMP–nanoparticle hybrids may combine membrane disruption with metal-ion release, ROS generation, and enhanced biofilm destabilization [144,145,146,147]. AMP integration with photodynamic therapy, photothermal therapy, oxygen-generating systems, exosomes, growth factors, or conductive biomaterials may further support simultaneous infection control and tissue repair [25,50,131,132,133,134,135,136,137,138,139,140,142,143,144,145,146,147,148,149,150,151,152,154,155,156,157,158,159,160].

Phage therapy and CRISPR-based antimicrobial approaches also represent emerging complementary strategies. Bacteriophages may provide pathogen-specific biofilm targeting, while CRISPR-based systems may theoretically eliminate resistance genes or selectively suppress pathogenic strains. However, these approaches remain early in the wound-care context and face challenges involving delivery, immune neutralization, bacterial escape, regulatory classification, and biosafety. Their most realistic near-term role may be as components of multimodal local therapies combined with AMPs, antibiofilm biomaterials, and conventional wound bed preparation.

### 9.5. Human-Relevant Models, Organoids and Standardized Antibiofilm Endpoints

A major priority for future AMP translation is the development of standardized and clinically predictive experimental models. As discussed in Section 5.7, the planktonic and mono-species assays on which many studies still rely poorly reproduce chronic wound complexity; future evaluation should instead prioritize mature polymicrobial biofilms, clinically derived isolates, and wound-like matrices that incorporate diabetic or ischemic tissue contexts and recurrent infection [72,73,74,75,76,119,120,121,128,129,130].

Human skin equivalents, ex vivo human skin, organotypic wound models, microfluidic wound-on-chip systems, and 3D bioprinted infected skin models may help bridge the gap between simplified in vitro assays and animal studies. These platforms could enable simultaneous evaluation of antimicrobial efficacy, keratinocyte migration, fibroblast activation, angiogenesis, macrophage polarization, biofilm persistence, and cytotoxicity. Although these models cannot fully replace in vivo systems, they may improve mechanistic understanding and reduce overreliance on acute rodent wounds.

Large-animal models, especially porcine wound systems, remain important for evaluating dressing adherence, exudate management, release kinetics, wound coverage, tissue integration, and repeated application protocols [165,186]. However, cost, ethical requirements, and infrastructure needs limit widespread use. A tiered evaluation framework may therefore be most appropriate: initial computational and in vitro screening, followed by mature polymicrobial biofilm models, human skin equivalents, diabetic or ischemic rodent models, and selected large-animal validation before clinical trials.

### 9.6. Regulatory, Manufacturing and Clinical Implementation Priorities

Clinical translation of AMP-enabled wound therapeutics will require coordinated progress in manufacturing, regulation, trial design, and reimbursement. Peptide synthesis must become scalable, reproducible, cost-effective, and compatible with good manufacturing practice requirements [17,119]. For complex biomaterial systems, additional challenges include sterilization, storage stability, release reproducibility, dressing mechanics, batch-to-batch consistency, and compatibility between peptide and carrier [18,19,128].

Regulatory classification will remain a major challenge because AMP-based dressings may fall between drugs, devices, biologics, and combination products. Smart systems containing peptides, nanoparticles, biosensors, conductive components, or exosome-like vesicles may face even more complex regulatory pathways [17,18,19,152,154,155,156,157,158,159,160]. Future development should therefore include early regulatory planning, standardized quality-control metrics, and clinically meaningful endpoints.

Clinical trials should move beyond bacterial reduction alone and include wound closure, time to closure, recurrence prevention, antibiotic-sparing effect, limb salvage, pain reduction, quality of life, and long-term safety [91,92,93,94,162,163,165,166,167,168,170,172,173,174,186]. In addition, trial protocols should carefully control for debridement, offloading, vascular status, glycemic control, wound duration, infection severity, and concomitant therapies. Without such standardization, promising AMP technologies may fail because of trial heterogeneity rather than true therapeutic inefficacy.

### 9.7. Concluding Perspective

Future success of AMP-enabled chronic wound therapeutics will likely depend not on AMP discovery alone, but on the integration of computational peptide engineering, smart biomaterial delivery, microbiome-informed personalization, clinically relevant models, and regulatory-ready translational pipelines. AI can accelerate identification and optimization of peptide candidates, but these candidates must be evaluated under wound-relevant conditions and delivered through biomaterials capable of overcoming proteolysis, biofilm barriers, exudate washout, inflammation, and impaired tissue regeneration.

The most promising next-generation systems will therefore be multifunctional, locally retained, pathology-responsive, and clinically practical. Ideally, they should combine antimicrobial and antibiofilm activity with immune modulation, oxidative-stress control, angiogenic support, and real-time monitoring. Such integrated platforms may ultimately shift chronic wound therapy from repeated nonspecific antimicrobial suppression toward precision regenerative infection control. The corresponding priority research directions are summarized in Table 9.

## 10. Conclusions

Antimicrobial peptides (AMPs) are emerging as multifunctional therapeutic agents capable of simultaneously addressing infection control, biofilm disruption, immune modulation, and tissue regeneration in chronic wounds. Their ability to target multidrug-resistant pathogens, interfere with quorum sensing, destabilize extracellular polymeric matrices, and promote angiogenesis and re-epithelialization positions them as highly attractive alternatives to conventional antibiotics in biofilm-associated wound infections. 

At the same time, biomaterial-based delivery systems have evolved from passive carriers into active therapeutic platforms that enhance peptide stability, prolong local retention, improve controlled release, and modulate the hostile chronic wound microenvironment. Hydrogels, electrospun nanofibers, nanoparticles, smart dressings, and stimuli-responsive systems collectively represent a major step toward integrated regenerative wound therapeutics capable of combining antimicrobial and tissue-repair functions within a single platform. 

Nevertheless, persistent biofilm eradication remains the central unmet clinical challenge. Mature polymicrobial biofilms exhibit profound antimicrobial tolerance, metabolic heterogeneity, immune evasion, and rapid recurrence after treatment, severely limiting the efficacy of conventional therapies. While AMPs and AMP-enabled biomaterials show considerable promise in overcoming these barriers, successful clinical translation will depend less on discovering additional peptides and more on addressing delivery, stability, manufacturing scalability, reproducibility, regulatory complexity, and clinically relevant validation models.

Ultimately, AMP-enabled biomaterials are shifting chronic wound care from passive infection management toward active, responsive, and regenerative therapy. Future progress will require multidisciplinary integration of biomaterials science, microbiology, immunology, artificial intelligence-assisted peptide engineering, and translational clinical research to transform these promising technologies into standardized and clinically effective wound therapeutics.

## Figures and Tables

**Table 1:** Major chronic wound pathogens, biofilm-associated adaptations, and implications for AMP-based therapeutics.

Pathogen	Predominant Wound Types	Major Virulence and Persistence Mechanisms	Biofilm-Associated Adaptations	Major Resistance Mechanisms	Key Challenges for AMP Therapy	Promising AMP/Biomaterial Strategies	Ref.
Methicillin-resistant *Staphylococcus aureus* (MRSA)	Diabetic foot ulcers, surgical wounds, pressure ulcers	Adhesins (FnBPs, clumping factors), cytotoxins, immune evasion, intracellular persistence, SCV formation	Dense EPS, intracellular reservoirs, metabolically heterogeneous populations	*mecA*-mediated β-lactam resistance, persister-cell formation, biofilm tolerance	Electrostatic peptide sequestration by EPS, intracellular persistence, protease-mediated AMP degradation	Membrane-disruptive AMPs, AMP hydrogels, protease-resistant peptide analogues, nanoparticle-assisted intracellular delivery	[78,79,80,81,82]
*Pseudomonas aeruginosa*	Burn wounds, diabetic ulcers, chronic exudative wounds	Quorum sensing, pyocyanin, phenazines, elastases, siderophores, metabolic plasticity	Thick EPS, anaerobic niches, polymicrobial dominance, hypoxia adaptation	Efflux pumps, metabolic dormancy, persisters, adaptive resistance	High EPS density, ROS-rich microenvironment, protease secretion, rapid adaptive mutation	Anti-QS peptides, ROS-responsive hydrogels, EPS-penetrating nanocarriers, multifunctional AMP dressings	[68,69,83,84,85]
*Enterococcus faecalis*	Diabetic foot ulcers, healthcare-associated wounds	Gelatinase, cytolysin, aggregation substances, persistence under nutrient stress	Stable polymicrobial biofilms, oxidative-stress tolerance	Intrinsic tolerance, VRE phenotype, horizontal gene transfer	High tolerance in multispecies biofilms, persistence under harsh wound conditions	Combination AMP-antibiotic therapy, synergistic peptide cocktails, sustained-release biomaterials	[76,86,87]
*Acinetobacter baumannii*	Burn wounds, military trauma, ICU-associated wounds	Environmental persistence, desiccation tolerance, surface colonization	Highly resilient surface-associated biofilms, prolonged abiotic survival	MDR/XDR phenotypes, carbapenemases, efflux systems	Extreme multidrug resistance, biofilm-associated persistence, hospital transmission	AMP-loaded nanocarriers, metallic nanoparticle hybrids, contact-killing coatings, electrospun antimicrobial scaffolds	[77,88,89,90]

Abbreviations: AMP, antimicrobial peptide; EPS, extracellular polymeric substance; MDR, multidrug resistant; SCV, small-colony variant; VRE, vancomycin-resistant enterococci; XDR, extensively drug resistant; QS, quorum sensing.

**Table 2:** Biomaterial platforms for AMP delivery in chronic wound therapy.

Biomaterial Platform	Representative Materials/Designs	Main AMP-Delivery Advantages	Biofilm-Related Functions	Regenerative/Wound-Healing Functions	Main Limitations	Ref.
Hydrogels	Chitosan, alginate, gelatin, collagen, hyaluronic acid, PEG, PVA hydrogels	High water content, moisture retention, local AMP retention, controlled release, conformability to wound bed	Protect AMPs from proteases, improve local concentration, support EPS penetration, reduce biofilm reformation after debridement	Maintain moist healing environment, absorb exudate, support keratinocyte/fibroblast migration, reduce inflammation	Weak mechanical strength, possible burst release, variable degradation, sterilization/storage issues	[18,19,131,132,133,134,135]
Self-assembling peptide hydrogels	Short peptide amphiphiles, supramolecular peptide nanofibers, AMP-based gelators	Peptide acts as both therapeutic and scaffold; high local peptide density; injectable and shear-thinning behavior	Intrinsic antibiofilm activity, matrix penetration, sustained AMP presentation, activity against polymicrobial biofilms	ECM-like nanofibrous architecture, cell infiltration, angiogenesis support, tissue remodeling	Cost, formulation complexity, peptide degradation, need for scalable manufacturing	[119,130,135]
Stimuli-responsive AMP hydrogels	pH-, ROS-, enzyme-, temperature-, glucose-responsive systems	On-demand AMP release triggered by infected wound conditions	Infection-selective AMP release, improved antibiofilm specificity, reduced premature peptide loss	May combine antimicrobial action with ROS scavenging, inflammation modulation, and diabetic wound microenvironment regulation	Complex formulation, difficult standardization, trigger variability between patients	[131,132,133,134,136,142]
Injectable/sprayable AMP hydrogels	Shear-thinning hydrogels, thermogels, sprayable polymer–peptide systems	Easy application to irregular wounds, burns, tunnels, cavities; homogeneous wound coverage	Improved distribution across heterogeneous biofilm-containing wound beds	Minimally invasive application, reduced dressing-change trauma, improved tissue contact	Retention may be reduced in highly exudative wounds; mechanical stability may be limited	[129,131,132,133,134]
Electrospun nanofibers	PCL, PLGA, PVA, chitosan, gelatin, alginate/PVA blends; coaxial/core–shell fibers	ECM mimicry, high surface area, tunable porosity, sustained AMP release	Prolonged antibiofilm exposure, prevention of recolonization, possible multilayer release against MDR pathogens	Support oxygen diffusion, fibroblast migration, keratinocyte proliferation, granulation tissue formation	Manufacturing complexity, solvent residues, scale-up challenges, peptide denaturation risk during processing	[137,138,139,140]
Films, membranes and AMP coatings	AMP-immobilized dressings, covalent coatings, contact-killing surfaces, enzyme-responsive coatings	Localized AMP presentation, reduced systemic exposure, anti-adhesive wound interfaces	Prevent bacterial adhesion, inhibit early biofilm formation, contact-mediated killing	Protect wound surface, reduce contamination, may integrate biosensing/visual infection monitoring	Immobilized AMPs may lose activity; limited penetration into established biofilms	[136,142,143]
Porous scaffolds, cryogels and sponges	Macroporous cryogels, collagen/chitosan sponges, composite scaffolds	High loading capacity, exudate absorption, sustained local AMP delivery	Provide prolonged AMP exposure and physical disruption of biofilm niches	Support cell infiltration, angiogenesis, granulation tissue, tissue defect filling	Bulkier materials, variable degradation, possible poor conformity to superficial wounds	[18,19,135,144]
Liposomes and lipid nanocarriers	Phospholipid vesicles, solid lipid nanoparticles, nanostructured lipid carriers	Protect AMPs from degradation, improve membrane interaction, reduce toxicity	Enhance penetration into biofilms and bacterial membranes; may improve AMP delivery to deeper layers	Biocompatible local delivery; may reduce irritation and improve residence time	Stability issues, leakage, oxidation, storage limitations	[119,144,145]
Polymeric nanoparticles	PLGA, chitosan, alginate, PEGylated nanoparticles	Controlled release, improved peptide stability, tunable size/charge	Better diffusion through EPS, enhanced local concentration, possible intracellular delivery	May combine AMP delivery with anti-inflammatory or regenerative cargos	Complex formulation, aggregation, regulatory challenges	[119,128,144,145]
Metallic nanoparticle–AMP hybrids	Silver, gold, zinc oxide, copper nanoparticles conjugated or co-loaded with AMPs	Synergistic antimicrobial action, reduced AMP dose, enhanced biofilm disruption	ROS generation, membrane damage, EPS destabilization, activity against MDR biofilms	Some systems may promote angiogenesis or reduce infection-associated inflammation	Cytotoxicity risk, metal accumulation, oxidative tissue damage, dose control needed	[144,145,146,147]
Nanoemulsions, micelles and dendrimers	Amphiphilic micelles, peptide-loaded nanoemulsions, dendrimer-peptide systems	Improve AMP solubility, multivalent peptide display, enhanced penetration	Biofilm penetration, membrane disruption, improved antibiofilm potency	Potential co-delivery of anti-inflammatory or regenerative molecules	Formulation instability, surfactant toxicity, complex safety profiling	[144,145,146]
Smart multifunctional AMP dressings	AMP + biosensor, AMP + photothermal/photodynamic agent, AMP + ROS/pH/enzyme-responsive system	Combines delivery, responsiveness, infection monitoring, and wound repair support	Triggered antibiofilm release, QS inhibition, EPS disruption, real-time infection response	Supports precision wound therapy, inflammation modulation, angiogenesis, wound closure	High complexity, regulatory uncertainty, manufacturing/scalability barriers	[133,134,135,136,137,138,139,140,142,143,144,145,146,147]

Abbreviations: AMP, antimicrobial peptide; ECM, extracellular matrix; EPS, extracellular polymeric substance; MDR, multidrug resistant; PEG, polyethylene glycol; PVA, polyvinyl alcohol; PCL, polycaprolactone; PLGA, poly(lactic-co-glycolic acid); ROS, reactive oxygen species; QS, quorum sensing.

**Table 3:** Pathology-responsive AMP biomaterial systems for chronic wound therapy. This table provides the referenced inventory of specific pathology-responsive platforms; their shared trigger–release–effect mechanism is depicted conceptually in Figure 8.

Trigger or System Class	Responsive AMP Biomaterial Strategy	AMP Delivery Mechanism	Therapeutic Advantage	Representative Platforms	References
Stimulus-responsive systems (triggered, on-demand AMP release)					
Alkaline/infection-associated pH	pH-responsive hydrogel swelling or degradation	Triggered AMP release in infected wound microenvironment	Infection-selective delivery, reduced premature release	pH-sensitive peptide hydrogels	[148,150,154]
ROS excess	ROS-responsive polymers or antioxidative hydrogels	Oxidation-sensitive release and ROS scavenging	Reduced oxidative damage plus antibiofilm activity	Antioxidative hydrogel dressings	[149,150]
Protease-rich exudate/MMP activity	Enzyme-cleavable coatings or linkers	Protease-triggered AMP liberation	Localized activation in infected/proteolytic wound regions	Enzyme-responsive AMP coatings	[151]
Multifunctional integration systems (constitutive, non-triggered AMP presentation)					
Mature biofilm EPS	Nanocarrier-mediated penetration	Sustained AMP exposure within deeper biofilm layers	Improved antibiofilm efficacy and peptide retention	AMP nanomaterials, polymeric nanoparticles	[155,157,158]
MDR microbial burden	AMP–metal nanoparticle synergy	Combined membrane disruption, ion release, ROS generation	Lower required peptide dose, enhanced biofilm killing	Gold, silver, copper, zinc oxide nanoparticle hybrids	[155,156]
Polymicrobial biofilms	Supramolecular/self-assembled peptide systems	High-density local AMP presentation	Biofilm disruption plus scaffold-like regenerative support	Self-assembled AMP nanostructures and hydrogels	[135,159]
Need for wound monitoring	Theranostic AMP dressings	Sensor-guided therapy or visual infection monitoring	Diagnosis and therapy in one platform	AIE visual AMP dressings, fluorescent systems	[160]

**Table 4:** Representative AMP-enabled antibiofilm biomaterial systems evaluated in chronic wound models.

AMP/System	Biomaterial Carrier	Target Pathogen/Biofilm	Antibiofilm Effect	Regenerative Effect	Model/Evidence Level	Translational Stage	Primary Translational Bottleneck	References
Pexiganan (22-aa α-helical magainin-2 analogue)	Topical cream	Mild diabetic foot infection pathogens	Broad antibacterial activity; limited mature biofilm evidence. MIC50/90 16/32 µg/mL against diabetic-foot pathogens; pivotal Phase 3 (OneStep) showed no superiority over vehicle, and pexiganan has not received FDA approval following the negative OneStep-1 and OneStep-2 trials [167,169].	Indirect improvement via infection reduction	Multicenter randomized clinical trial	Clinical-stage investigational	Regulatory positioning and endpoint complexity	[167,168]
LL-37 (37-aa human cathelicidin; α-helical)	Topical peptide formulation	Chronic ulcer-associated microbial burden	Moderate antimicrobial and immunomodulatory activity	Enhanced healing of venous leg ulcers	Randomized clinical trials	Clinical-stage investigational	Proteolytic instability and dose optimization	[170,172,173]
Human β-defensin-2 (human β-defensin; β-sheet, disulfide-stabilized)	Alginate-based hydrogel	MRSA-infected diabetic wounds	Sustained antimicrobial peptide release and bacterial reduction	Accelerated diabetic wound closure	Diabetic murine wound model	Preclinical	Lack of mature polymicrobial biofilm validation	[163]
MP-L [I5R8]	Dual-stimuli-responsive hydrogel	Drug-resistant wound-associated bacteria	Controlled AMP release and biofilm suppression	Improved tissue repair and inflammatory modulation	Preclinical infected wound model	Preclinical	Formulation complexity and scalability	[134]
Short synthetic peptide systems (short self-assembling peptide amphiphiles)	Supramolecular nanocomposite hydrogel	Polymicrobial biofilms	Mature biofilm disruption and EPS destabilization	Accelerated infected wound healing	In vitro + preclinical wound models	Preclinical	Manufacturing reproducibility and long-term stability	[135]
EM86	Alginate/PVA electrospun nanofibers	MDR *Pseudomonas aeruginosa*	Sustained local antimicrobial activity	Enhanced wound repair in infected mice	BALB/c infected wound model	Preclinical	Limited translational validation in chronic wounds	[140]
AMP nanomaterials	Polymeric nanoparticles and nanostructures	MDR Gram-negative and biofilm-forming bacteria	Improved biofilm penetration and peptide stability	Platform-dependent regenerative support	In vitro and preclinical evidence	Preclinical	Toxicity and regulatory uncertainty	[144,145]
Self-assembled AMPs	Nanostructured peptide assemblies	Polymicrobial mature biofilms	Sustained antimicrobial activity and membrane disruption	Biomimetic ECM-like regenerative properties	Experimental biomaterial studies	Preclinical	Structural stability and scale-up	[146]
AMP–gold nanoparticle hybrids	Metallic nanoparticle conjugates	Resistant Gram-positive and Gram-negative biofilms	Synergistic membrane disruption and ROS amplification	Potential pro-regenerative effects	In vitro and preclinical studies	Preclinical	Nanotoxicity and manufacturing consistency	[147]
Enzyme-responsive AMP coatings	Bio-inspired surface coatings	Biofilm-forming wound pathogens	Infection-triggered AMP release and contact killing	Improved local tissue compatibility	Experimental coating systems	Preclinical	Sterilization and regulatory classification	[136]
Smart AMP-engineered hydrogels	Responsive hydrogel systems	Chronic wound biofilms	Triggered AMP release and antibiofilm activity	Anti-inflammatory and regenerative activity	Preclinical wound-healing studies	Preclinical	Complexity of multi-responsive design	[142]
AMP-modified AIE dressing	Fluorescent composite wound dressing	Infected wound biofilms	Antimicrobial activity with infection visualization	Accelerated infected wound healing	Preclinical wound model	Preclinical	Validation of sensing accuracy and clinical usability	[143]
pH/ROS-responsive AMP systems	Smart multifunctional hydrogels	Chronic infected wound microenvironments	Pathology-triggered antimicrobial release	Immune microenvironment modulation and antioxidative activity	Preclinical wound studies	Preclinical	Trigger variability and release reproducibility	[148,149]
Conductive AMP biomaterials	Electroconductive hydrogels	Infected chronic wounds	Antibacterial activity enhanced by electrical stimulation	Improved angiogenesis and wound closure	Experimental wound-healing models	Preclinical	Electrical safety and integration complexity	[150,151]
Exosome-assisted AMP systems	Exosome-loaded hydrogels and vesicle systems	Infected diabetic wounds	Enhanced local delivery and inflammatory modulation	Strong regenerative and angiogenic effects	Diabetic wound models	Early preclinical	Standardization and regulatory classification	[152,154,155]
Smart biosensing AMP dressings	Theranostic wearable dressings	Infection-associated wound biomarkers	Responsive antimicrobial activation and infection sensing	Personalized wound monitoring	Experimental biosensing systems	Early translational	Sensor calibration and clinical validation	[156,157,158,159]
Bacterial enzyme-responsive hydrogels	Triggered-release hydrogel systems	Infected wound biofilms	Protease-triggered antibiotic/AMP release	Improved infection-responsive therapy	Advanced experimental wound systems	Preclinical	Translational reproducibility and cost	[160]

Abbreviations: AIE, aggregation-induced emission; AMP, antimicrobial peptide; ECM, extracellular matrix; EPS, extracellular polymeric substance; MDR, multidrug-resistant; MRSA, methicillin-resistant *Staphylococcus aureus*; PVA, polyvinyl alcohol; ROS, reactive oxygen species. AMP class/type is indicated where a defined peptide was evaluated; quantitative antibiofilm efficacy (e.g., log10 CFU reduction) is reported inconsistently across the cited studies, reflecting the lack of standardized biofilm endpoints discussed in Section 5.7 and Section 8.1.2 and limiting direct cross-study comparison.

**Table 5:** Mechanism- and target-resolved comparison of representative AMP-enabled antibiofilm systems with a defined peptide. This table complements the descriptive inventory in Table 4 by resolving each system by bacterial target, peptide class, trigger mechanism, specific antibiofilm mode of action, and reported quantitative efficacy.

AMP/System (Ref.)	AMP Class/Structural Type	Bacterial Target (Gram ±)	Carrier & Trigger Mechanism	Specific Antibiofilm Mechanism	Reported Efficacy (Quantitative Endpoint)
Pexiganan/MSI-78 [167,168,169]	Magainin-2 analogue; cationic α-helical, 22 aa	Broad-spectrum Gram-positive and Gram-negative	Topical cream; no responsive trigger	Membrane disruption; limited evidence against mature biofilm	Planktonic MIC50/90 16/32 µg/mL; pivotal Phase 3 (OneStep) showed no superiority over vehicle; biofilm log10 CFU reduction NR
LL-37 [170,172,173]	Human cathelicidin; cationic α-helical, 37 aa	Broad-spectrum Gram ± wound flora	Topical peptide formulation; no responsive trigger	Membrane disruption, immunomodulation, quorum-sensing interference	Improved venous-ulcer healing in RCTs; biofilm log10 CFU reduction NR
Human β-defensin-2 [163]	β-defensin; β-sheet, disulfide-stabilized	MRSA (Gram-positive)	Alginate hydrogel; sustained (non-triggered) release	Sustained peptide release with bacterial-load reduction	Accelerated closure in diabetic murine wound; log10 CFU reduction NR
MP-L [I5R8] [134]	Designed cationic peptide analogue	Drug-resistant wound-associated bacteria (Gram ±)	Dual-stimuli (pH/enzyme)-responsive hydrogel	Triggered, on-demand release with biofilm suppression	Preclinical infected-wound model; quantitative endpoint NR
EM86 [140]	Synthetic cationic AMP	MDR *Pseudomonas aeruginosa* (Gram-negative)	Alginate/PVA electrospun nanofibers; no trigger	Sustained local antimicrobial activity	Enhanced repair in BALB/c infected model; log10 CFU reduction NR
Self-assembling peptide systems [135]	Short self-assembling peptide amphiphiles	Polymicrobial biofilms (Gram ±)	Supramolecular nanocomposite hydrogel; constitutive	Mature-biofilm disruption and EPS destabilization	Preclinical (in vitro + wound model); quantitative endpoint NR
pH/ROS-responsive AMP hydrogel [148,149]	Cationic peptide (sequence not specified in source)	Drug-resistant biofilm in diabetic wound (Gram ±)	Peptide hydrogel; pH- (and ROS-) triggered release	Pathology-triggered release; biofilm eradication with anti-inflammatory action	Enhanced eradication and closure (qualitative); log10 CFU reduction NR
AMP–gold nanoparticle hybrids [147,155,156]	AMP conjugated to metallic (Au) nanoparticles	Resistant Gram ± biofilms	Metallic nanoparticle conjugate; no trigger	Synergistic membrane disruption + ROS amplification; lowers effective peptide concentration	Improved biofilm penetration; reduced effective concentration (value NR)

Abbreviations: AMP, antimicrobial peptide; EPS, extracellular polymeric substance; MDR, multidrug-resistant; MIC, minimum inhibitory concentration; MRSA, methicillin-resistant *Staphylococcus aureus*; NR, not reported; PVA, polyvinyl alcohol; RCT, randomized controlled trial; ROS, reactive oxygen species. Where a primary study did not report a standardized quantitative biofilm endpoint (e.g., log10 CFU reduction or minimum biofilm eradication concentration), the entry is marked NR; this heterogeneity reflects the absence of standardized biofilm endpoints discussed in Section 5.7 and Section 8.1.2 and limits direct cross-study comparison.

**Table 6:** Primary in vivo and clinical studies of AMP-enabled wound systems with quantitative outcomes. This table consolidates original studies (with reported magnitudes) underlying Section 6, Section 7 and Section 8, complementing the platform inventory in Table 4 and the mechanism-resolved comparison in Table 5. NR, not reported; MDR, multidrug-resistant; MRSA, methicillin-resistant *Staphylococcus aureus*; RCT, randomized controlled trial; PVA, polyvinyl alcohol.

Peptide	Carrier/System	Model/Organism	Antimicrobial Outcome	Regenerative Outcome	Evidence Level	Ref.
Self-assembling peptide + Ag/Au nanoparticles	Supramolecular nanocomposite hydrogel	Mono- & polymicrobial biofilms; infected wound in vivo	≥1.5-log10 viability reduction; outperformed a last-resort antibiotic and an Ag ointment	Accelerated infected-wound healing	Preclinical in vivo	[135]
Cationic peptide (pH-sensitive)	pH-sensitive hydrogel	Drug-resistant biofilm-infected diabetic wound (mouse)	Eradication of drug-resistant bacteria in vitro and in vivo	Reduced inflammation; accelerated closure	Preclinical in vivo	[131]
EM86	γ-irradiated alginate/PVA electrospun nanofibers	MDR *P. aeruginosa*; BALB/c infected wound	Sustained local antimicrobial activity (log10 NR)	Enhanced infected-wound repair	Preclinical in vivo	[140]
Human β-defensin-2	Ca-cross-linked alginate hydrogel	MRSA-infected streptozotocin-diabetic mouse	Bacterial-load reduction (log10 NR)	Increased Ki67+/CD31+ cells; pro-angiogenic, anti-inflammatory	Preclinical in vivo	[162]
D-Bac8c2,5Leu	Methylcellulose hydrogel	Mono- & polymicrobial *S. aureus* + *P. aeruginosa* biofilms (static & flow)	2–3-log10 reduction; disrupts pre-formed biofilm	Low human-cell cytotoxicity (regeneration NR)	In vitro	[161]
Jelleine-1 + 8Br-cAMP	Carrier-free self-assembled hydrogel	MRSA-infected diabetic wound (mouse)	Bacterial clearance	Accelerated wound closure	Preclinical in vivo	[164]
HBD peptide	Ultrasound-responsive hydrogel	Diabetic mouse wound (biofilm)	Antibiofilm; ~400 µm penetration on activation	Complete closure; increasedVEGF-A, CD31, α-SMA	Preclinical in vivo	[153]
17BIPHE2 (engineered LL-37)	F127/PCL core–shell nanofibers	MRSA USA300; type II diabetic biofilm wound	~5-log CFU reduction; full eradication with debridement	Supports chronic-wound closure	Preclinical in vivo	[141]
LL-37	Topical peptide formulation	Hard-to-heal venous leg ulcers (patients)	Clinical endpoint; standardized log10 NR	~6-/3-fold healing-rate at 0.5/1.6 mg/mL; negative larger phase IIb	Clinical (RCT)	[170,171]

**Table 7:** Comparative analysis of the principal experimental models used to evaluate AMP-enabled antibiofilm wound systems, summarizing what each model captures together with its main advantages, limitations, and translational relevance. CDC, Centers for Disease Control and Prevention (biofilm reactor); CLSI, Clinical and Laboratory Standards Institute; EUCAST, European Committee on Antimicrobial Susceptibility Testing; EPS, extracellular polymeric substance; MBC, minimum bactericidal concentration; MBEC, minimum biofilm-eradication concentration; MBIC, minimum biofilm-inhibitory concentration; MIC, minimum inhibitory concentration; STZ, streptozotocin.

Translational Relevance	Key Limitations	Key Advantages	What It Captures	Experimental Model
Low for chronic wounds; useful only as an initial screen	Ignores biofilm tolerance, EPS barrier, and persisters; overestimates in vivo potency	Standardized (CLSI/EUCAST); simple, inexpensive, high-throughput	Activity against free-floating, actively dividing cells	Planktonic susceptibility (broth/agar MIC, MBC)
Moderate; the standard antibiofilm screen	Usually mono-species with short maturation; no shear, flow, or host milieu	Reproducible and quantitative; moderate throughput; captures biofilm tolerance	Surface-attached biofilm formation and eradication (MBIC/MBEC)	Static biofilm assays (microtiter; Calgary/MBEC device)
Higher; closer to in vivo biofilm physiology	Technically demanding; low throughput; specialized equipment	More physiologic architecture, EPS maturity, and gradients	Mature biofilm under shear and nutrient/oxygen gradients	Flow-based biofilm systems (flow cell, drip-flow, CDC reactor)
Moderate to high; bridges in vitro and in vivo	Limited tissue-viability window; no systemic immunity or perfusion; donor variability	Real host matrix and microanatomy; no live-animal variables	AMP activity within an authentic tissue matrix	Ex vivo/explant models (human or porcine skin)
Moderate; standard preclinical efficacy stage	Rodent skin heals largely by contraction; small wounds; cost and ethical constraints	Whole-organism healing, immunity, and in vivo toxicity readouts	Infection and healing in a live host	Small-animal wound models (mouse, rat)
High; closest preclinical surrogate for chronic wounds	Expensive; low throughput; specialized facilities; ethical and logistic burden	Porcine skin closely resembles human; diabetic models reproduce chronicity	Human-like skin and impaired, chronic-type healing	Large-animal and diabetic models (porcine; db/db or STZ-diabetic)
Definitive	Costly and slow; heterogeneous populations; regulatory and endpoint complexity	Definitive evidence under clinical conditions	Real-world efficacy, safety, and healing endpoints	Human/clinical studies

**Table 8:** Representative original studies applying artificial intelligence to antimicrobial-peptide discovery, activity and antibiofilm prediction, generative design, template optimization, and toxicity filtering, with their wet-laboratory validation and principal limitations. AMP, antimicrobial peptide; CLaSS, controlled latent attribute space sampling; DBAASP, Database of Antimicrobial Activity and Structure of Peptides; MD, molecular dynamics; MDR, multidrug-resistant; MIC, minimum inhibitory concentration; MRSA, methicillin-resistant *Staphylococcus aureus*; ML, machine learning; QSAR, quantitative structure–activity relationship; RNN, recurrent neural network; VAE, variational autoencoder.

Computational Method	Dataset/Task	Generated or Prioritized Candidates	Wet-Lab Validation	Relevance to Biofilms/Wounds	Principal Limitations	Ref.
ML mining of metagenomes (AMPSphere)	Global microbiome AMP discovery	~10^6^ candidate peptides; subset synthesized	Subset chemically synthesized, active vs. clinical pathogens	Broad discovery; not wound- or biofilm-specific	Vast majority untested; standard-assay activity only	[175]
Peptide-language/generative model	De novo design vs. multidrug-resistant bacteria	Generative candidate AMPs	In vitro and in vivo activity vs. drug-resistant strains	MDR pathogens; not biofilm-resolved	Not validated under wound or biofilm conditions	[176]
Genetic-algorithm template optimization	Optimize guava peptide Pg-AMP1	Guavanin series (lead, guavanin 2)	Synthesis; potent vs. Gram-negative bacteria	*A. baumannii* (wound-relevant pathogen)	Narrow spectrum; single natural template	[184]
3D-QSAR antibiofilm model	Antibiofilm activity of IDR-1018 variants	100,000 virtual sequences screened (~85% accuracy)	In vitro MRSA biofilm + in vivo mouse abscess	Directly antibiofilm and wound-relevant (MRSA)	Single scaffold; MRSA-focused	[181]
Deep generative autoencoder (CLaSS) + MD + classifiers	De novo non-toxic broad-spectrum AMPs	~90,000 generated; 20 synthesized	2 potent AMPs; in vitro + in vivo (mice); hemolysis/toxicity	MDR *K. pneumoniae*; toxicity-aware	Planktonic endpoints; no biofilm or wound model	[182]
RNN trained on activity + hemolysis (DBAASP)	Design of non-hemolytic AMPs	28 synthesized (≥5 mutations from training)	8 non-hemolytic AMPs vs. *P. aeruginosa*, *A. baumannii*, MRSA	Wound pathogens; hemolysis-aware design	Planktonic MIC; no biofilm or in vivo data	[185]
VAE design + MIC regressors + cell-free screen	High-throughput de novo AMP development	~500,000 generated; 500 screened	30 functional AMPs; 6 broad-spectrum; toxicity tested	MDR pathogens; rapid validation	No wound or biofilm validation	[183]

**Table 9:** Priority Research Directions for Next-Generation AMP Wound Therapeutics.

Priority Area	Current Limitation	Recommended Future Direction	Representative References
AI-guided AMP design	Most predictive models rely predominantly on planktonic MIC datasets and insufficiently evaluate antibiofilm efficacy, cytotoxicity, protease stability, or wound-microenvironment performance	Develop multimodal AI platforms integrating antibiofilm datasets, wound-fluid proteolysis, immunomodulatory activity, toxicity prediction, and biomaterial compatibility	[175,176,177,178,179,180]
Biofilm testing systems	Heavy dependence on immature mono-species biofilms with limited clinical relevance	Standardize mature polymicrobial biofilm models using clinically derived isolates and chronic wound-like matrices	[72,73,74,75,76,95,97,111,120,121,128,129,130]
Translational wound models	Acute murine wounds inadequately reproduce chronic diabetic or ischemic wound pathology	Increase use of diabetic, ischemic, recurrent-infection, humanized, and porcine wound models	[35,130,165,172,186]
AMP stability and delivery	Rapid degradation by wound proteases, burst release, and insufficient local retention reduce therapeutic efficacy	Develop protease-resistant peptides and pathology-responsive delivery systems with controlled release kinetics	[16,17,48,119,131,132,133,134,135,136,148,149]
Smart biomaterial integration	Conventional dressings remain passive and lack adaptive responsiveness to wound microenvironment changes	Engineer multifunctional hydrogels and nanofibers responsive to pH, ROS, bacterial enzymes, electrical stimuli, or inflammatory biomarkers	[131,132,133,134,135,136,137,138,139,140,142,143,144,145,146,147,148,149,150,151,152,154,155,156,157,158,159,160,162,163]
Personalized wound therapy	Current approaches insufficiently account for wound microbiome heterogeneity and patient-specific pathology	Integrate microbiome sequencing, biosensors, AI-guided peptide selection, and precision biomaterials	[12,74,75,76,156,157,158,159]

## Data Availability

No new data were created or analyzed in this study. Data sharing is not applicable to this article.
